# Comparison of efficacy and safety of non-oral therapeutic interventions for zoster-associated pain: a systematic review and network meta-analysis

**DOI:** 10.3389/fneur.2026.1711536

**Published:** 2026-01-27

**Authors:** Yuchen Hao, Xiange Liu, Xinyi Ma, Tao Sun

**Affiliations:** 1 Department of Pain Management, Shandong Provincial Hospital, Cheeloo College of Medicine, Shandong University, Jinan, Shandong, China; 2 Departments of Pain Management, Shandong Provincial Hospital Affiliated to Shandong First Medical University, Jinan, Shandong, China

**Keywords:** chemical selective neurolysis, combination therapy, dorsal root ganglion destruction, minimally invasive neuromodulation, network meta-analysis, non-oral therapeutic interventions, zoster-associated pain

## Abstract

**Background:**

Zoster-associated pain (ZAP) encompasses acute, subacute, and postherpetic neuralgia stages. It often results in persistent sensory abnormalities and substantial impairment of quality of life. Although oral pharmacotherapy remains the first-line and foundational approach, its effectiveness may be limited in some patients; accordingly, non-oral interventions are investigated as complementary or escalated strategies. However, high-quality evidence investigating the relative efficacy and safety of these interventions remains scarce.

**Objective:**

This study intended to systematically evaluate the efficacy and safety of a variety of non-oral therapeutic interventions for ZAP, thereby providing evidence to inform clinical decision-making.

**Methods:**

Web of Science, Cochrane Library, Embase, and PubMed were searched to identify eligible randomized controlled trials (RCTs). Data from the included studies were extracted, and the risk of bias was examined via the Cochrane Risk of Bias Tool 2.0. A Bayesian network meta-analysis (NMA) was carried out to compare different interventions, and surface under the cumulative ranking curve (SUCRA) probabilities were utilized to rank relative treatment effects. STATA 18 and R version 4.4.2 were employed to conduct statistical analyses.

**Results:**

Fifty-three RCTs involving 4,973 patients were included. The NMA showed that chemical selective neurolysis provided the greatest pain relief compared with other treatments (standardized mean difference [SMD]: 4.34; 95% credible interval [CrI]: 2.18 to 6.49). The analysis showed no statistically significant increase in the incidence of adverse events (AEs) (risk ratio: 32.05; 95% CrI: 0.57 to 3,326.64), though the extremely wide CrI indicated substantial uncertainty in this risk estimate. There were no serious complications. Minimally invasive central nervous system neuromodulation combined with topical and peripheral chemical interventions demonstrated the most favorable overall benefits in both pain relief (SMD = 3.41, 95% CrI: 1.08 to 5.73) and sleep improvement (SMD = 3.71, 95% CrI: 1.86 to 5.77). This was followed by minimally invasive peripheral nerve modulation combined with systemic pharmacological analgesia. Regarding safety, no statistically significant differences in AE incidence were found among interventions. However, SUCRA rankings suggested that oral medication and minimally invasive central nervous system neuromodulation had the most favorable safety profiles.

**Conclusion:**

Combination therapies utilizing minimally invasive neuromodulation show favorable potential in managing ZAP. While chemical selective neurolysis may benefit refractory cases, its use necessitates careful ethical and safety evaluation. Due to the low overall certainty of the evidence, these findings should be interpreted with caution, underscoring the critical need for rigorous future research to confirm long-term outcomes.

**Systematic review registration:**

https://www.crd.york.ac.uk/PROSPERO, identifier CRD420251059913.

## 1 Introduction

Herpes zoster is caused by the reactivation of the latent varicella-zoster virus. The condition is characterized by inflammation of the dorsal root ganglion (DRG), which results in a unilateral, dermatomal rash accompanied by zoster-associated pain (ZAP) (1). Based on disease progression, ZAP is classified into three clinical stages: (i) acute ZAP, defined as pain persisting for ≤ 30 days after onset, (ii) subacute ZAP, persisting after vesicle healing but resolving within 3 months, and (iii) postherpetic neuralgia (PHN) (2). PHN is the most prevalent complication of Herpes zoster. PHN is marked by mechanical allodynia, thermal hyperalgesia, and spontaneous electric shock–like pain. Approximately 22% of patients experience pain lasting more than one month, which subsequently progresses to PHN. PHN can result in depression, weight loss, sleep disturbances, chronic fatigue, elevated risks of cardiovascular disease and suicide, and substantial healthcare and economic burdens (3).

Current therapeutic strategies for ZAP include conventional pharmacological treatments, various interventional therapies, and complementary and alternative therapies, such as mindfulness meditation (4). Oral pharmacotherapy is the cornerstone of the first-line management for ZAP. Frequently prescribed medications include tricyclic antidepressants, anticonvulsants (e.g., pregabalin, gabapentin), and opioids (e.g., tramadol) (4, 5). However, the use of tricyclic antidepressants is often limited due to adverse effects such as somnolence, constipation, and dry mouth. These drugs have gradually been replaced for older patients because their pronounced sedative and anticholinergic effects increase the risk of falls (6). Lidocaine patches are generally safe and well-tolerated; they can be combined with systemic agents to achieve additive effects (7). Consequently, non-oral interventions are recommended when conventional oral therapies are inadequate, when pain impairs physical function or quality of life, or when adverse effects limit tolerance. These interventions include nerve block, minimally invasive central/peripheral nervous system neuromodulation, targeted peripheral/superficial electrical neuromodulation, physical therapy and energy medicine, medical oxidant therapy, biological therapy, and chemical selective neurolysis (e.g., doxorubicin-mediated DRG destruction) (8). Among these interventions, pulsed radiofrequency—a type of minimally invasive peripheral nerve modulation—is a safe and effective treatment for cervical, lumbar, and sacral ZAP. It reduces pain intensity and improves quality of life in patients who do not respond to standard treatment. Compared with nerve blocks, pulsed radiofrequency provides longer-lasting modulation of pain sensitization. Unlike radiofrequency thermocoagulation, pulsed radiofrequency avoids tissue destruction and lowers the risk of dysesthesia. However, its long-term efficacy in refractory cases is inferior to that of minimally invasive central nervous system neuromodulation (9). A 2025 meta-analysis by Liu et al. confirmed that minimally invasive central nervous system neuromodulation (particularly short-term spinal cord stimulation [SCS]) yields significant efficacy and favorable safety in PHN treatment (10). Other non-pharmacological and non-interventional therapies, such as superficial electrical neuromodulation and cognitive behavioral therapy, have not been sufficiently studied for their efficacy in treating neuropathic pain. Thus, their role in patient management remains uncertain (11).

Recent systematic reviews and meta-analyses have examined the therapeutic effects of various interventions for ZAP. However, these interventions have been limited in scope, and comparisons across different neuromodulation techniques, energy-based physical therapies, drug infusion treatments, immunotherapies, combination therapies, as well as complementary and alternative medicine remain lacking (12–16). Network meta-analysis (NMA) is an extension of traditional meta-analysis that can integrate direct and indirect evidence across multiple interventions. This provides a systematic framework for assessing complex treatment effects and offers comprehensive guidance for clinical decision-making or policy development (17). Therefore, this study conducted a systematic review of articles examining various interventions for ZAP and employed NMA to quantify the relative efficacy and safety of different strategies. The aim was to address the clinical challenge of suboptimal responses to conventional oral therapies in individuals with ZAP by providing an evidence-based framework for individualized treatment decisions.

## 2 Methods

This study was carried out following the Preferred Reporting Items for Systematic Reviews and Meta-Analyses (PRISMA) guidelines, including the extension for NMA (PRISMA checklist) (18). The study protocol was prospectively registered in the International Prospective Register of Systematic Reviews (CRD420251059913).

### 2.1 Search strategy

Embase, Web of Science, the Cochrane Library, and PubMed were comprehensively searched from inception to May 23, 2025. Studies were restricted to those written in English. The search strategy was designed by combining medical subject headings and free-text terms. The keywords used included herpes zoster, neuralgia, and pain. In addition, reference lists of relevant studies and gray literature were manually reviewed to identify eligible articles. Supplementary file 1 presents the full search strategy.

### 2.2 Inclusion and exclusion criteria

Inclusion criteria encompassed: (i) participants: adults (≥ 18 years) diagnosed with ZAP; (ii) interventions and comparators: the intervention of interest was any procedure or therapy aimed at pain relief, categorized according to its primary mechanism of action into one of the following: nerve blocks, neuromodulation therapies (including non-invasive and invasive techniques), energy-based and physical therapies, biological therapies, drug and chemical therapies (encompassing both localized and systemic administration), conventional and alternative medicine, or combination therapies. Permissible comparators included standard pharmacotherapy (e.g., first-line oral agents such as pregabalin or gabapentin), placebo, sham procedures, or any intervention differing from the investigated treatment; (iii) outcomes: the primary outcome was pain intensity measured by the Numerical Rating Scale (NRS), Visual Analog Scale (VAS), Zoster Brief Pain Inventory (BPI), or Short-Form McGill Pain Questionnaire version 2. Secondary outcomes included sleep quality assessed by the Insomnia Severity Index (ISI), NRS-Sleep, Sleep Quality Scale, Sleep Disturbance Scores, Quality of Sleep, or Sleep Impairment Scale, as well as adverse events (AEs). If outcome definitions differed, original results were classified according to prespecified standards.

Exclusion criteria included: (i) animal or cell studies, case reports, letters, editorials, study protocols, reviews, and conference abstracts; (ii) studies with missing or erroneous data; (iii) duplicate publications; (iv) studies without accessible full texts; (v) studies with overlapping participant populations.

### 2.3 Data extraction

All selected articles were imported into EndNote 21. Two reviewers (Hao Yuchen and Liu Xiange) independently screened titles and abstracts according to the eligibility criteria, followed by full-text screening. Disagreements were addressed through discussion or by consulting a third reviewer (Sun Tao). Data were independently extracted by two reviewers via a predefined electronic form. Collected information encompassed: country, year of publication, study design, the first author, intervention and comparator details, sample size, follow-up duration, baseline characteristics of participants, and outcome measures.

### 2.4 Quality assessment

Two reviewers independently appraised the quality of eligible studies via the Cochrane Risk of Bias tool for randomized trials (RoB 2) (19). The assessment covered five domains: bias arising from the randomization process, bias due to deviations from intended interventions, bias due to missing outcome data, bias in measurement of the outcome, and bias in selection of the reported result. Each domain was evaluated according to the RoB 2 algorithm and categorized as “low risk,” “some concerns,” or “high risk.” The overall risk of bias for each study was determined by the highest risk level across all domains: “high risk” if any domain was rated as high risk, “some concerns” if no domain was rated as high risk but at least one domain raised some concerns, and “low risk” if all domains were rated as low risk. Disagreements were resolved through consensus or adjudication by a third reviewer.

### 2.5 Statistical analysis

For continuous outcomes (e.g., pain relief, sleep quality), the effect size was expressed as the standardized mean difference (SMD), corrected for small sample sizes using Hedges’ g. To optimize clinical homogeneity and reflect intermediate- to long-term efficacy, the analysis preferentially utilized the mean change from baseline to the primary follow-up endpoint (predominantly at 6 months) and its corresponding standard deviation. This approach synthesized data reported using various scales, including the VAS, NRS, BPI, and ISI. For dichotomous outcomes (e.g., AEs), the risk ratio (RR) served as the effect measure. All effect estimates were presented with their 95% credible intervals (CrI). A Bayesian random-effects NMA model was fitted for each outcome using the gemtc package in R to account for between-study heterogeneity. Vague prior distributions were specified: normal (mean 0, variance 10,000) for treatment effects and uniform (0, 5) for the between-study standard deviation (*τ*). For multi-arm trials, we split them into independent two-arm comparisons against a common control group and included them in the pairwise network. Models were estimated using a Markov chain Monte Carlo (MCMC) simulation (20). Four chains were run, each with 10,000 burn-in iterations, followed by 50,000 sampling iterations. The thinning interval was set to 10, and the dispersal factor was set to 2.5. Convergence was assessed by confirming that the potential scale reduction factor (PSRF) was below 1.05 for all key parameters and by visually inspecting trace plots. The core NMA assumptions were evaluated. Transitivity was assessed by comparing the distribution of potential effect modifiers (e.g., disease stage, mean patient age, proportion of female patients, baseline pain score, follow-up duration, lesion site, sample size, and year of publication) across treatment comparisons (17). Heterogeneity within pairwise comparisons was quantified using the I^2^ statistic. Inconsistency was evaluated locally via the node-splitting method and globally via the design-by-treatment interaction model (21). To explore heterogeneity sources, a Bayesian meta-regression was conducted with the aforementioned covariates. A covariate’s impact was considered statistically significant if the 95% CrI of its regression coefficient excluded 0. The relative ranking of interventions was summarized using the surface under the cumulative ranking curve (SUCRA). Publication bias was evaluated through a composite approach that employed a contoured-enhanced funnel plot to assess visual symmetry and an Egger’s test to measure quantitative asymmetry (a *p*-value < 0.05 suggested potential publication bias or heterogeneity). The Confidence in Network Meta-Analysis (CINeMA) framework was utilized to grade the certainty of evidence across six domains: risk of bias, reporting bias, heterogeneity, imprecision, indirectness, and incoherence. Each domain was rated as having high, moderate, low, or very low certainty. STATA (version 18) and R (version 4.4.2) were used for all statistical analyses.

## 3 Results

### 3.1 Literature search and selection process

**Figure 1:**
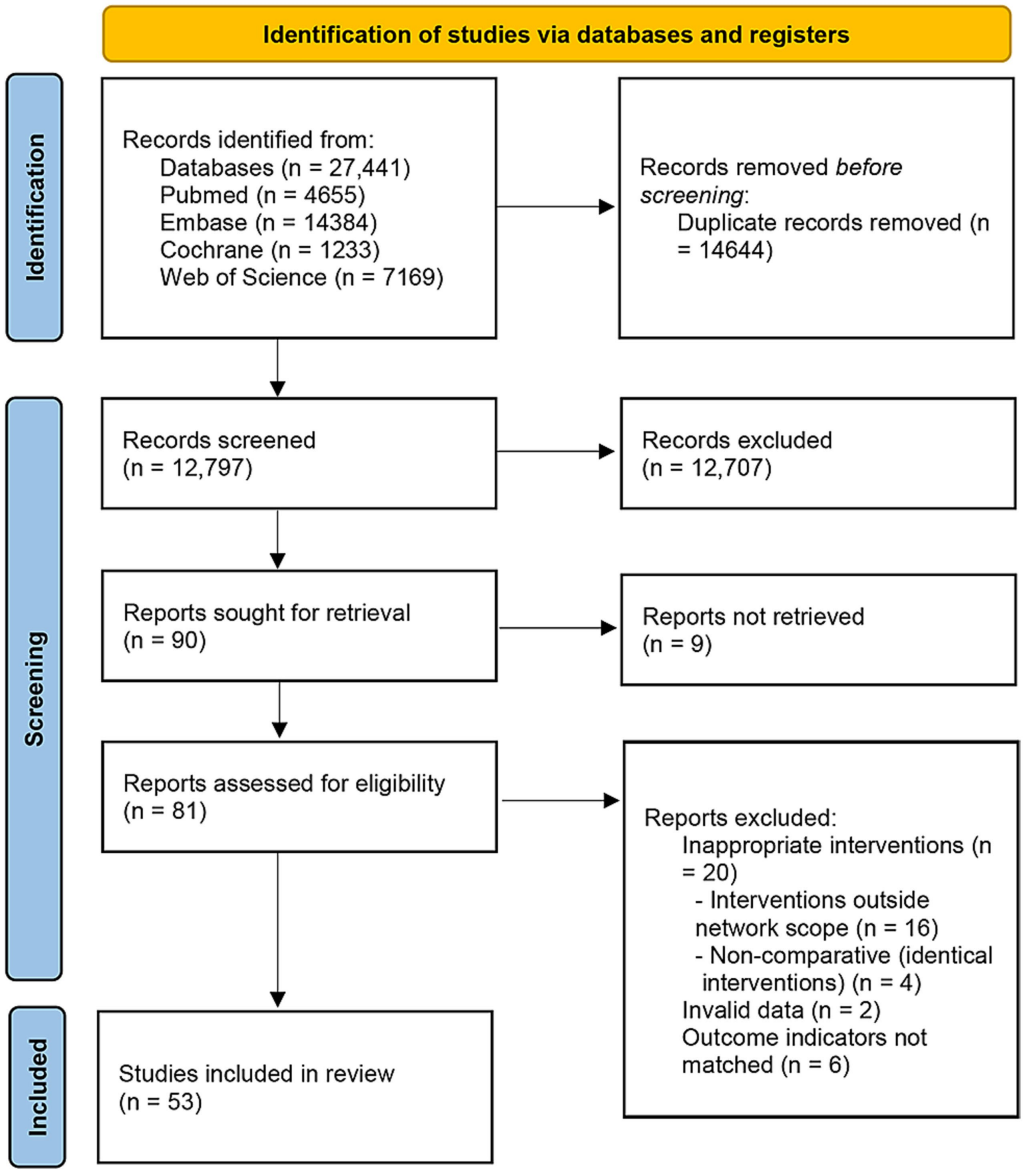
Study selection flowchart.

A total of 27,441 records were obtained from the databases, of which 14,644 were duplicate publications and were removed. After screening the titles and abstracts, an additional 12,707 studies were removed. The remaining studies were assessed in full text based on the inclusion and exclusion criteria, and more studies were removed. Ultimately, 53 studies were included in the NMA (22–74). Figure 1 illustrates the detailed screening process.

### 3.2 Characteristics of included studies and quality assessment

The 53 included studies were conducted across eight countries (China, Greece, the Netherlands, Egypt, South Korea, the USA, India, and Denmark) and enrolled 4,973 participants, with 52.09% being female. Participants’ ages ranged from 35 to 89 years old. The eligible studies evaluated multiple interventions targeting ZAP. To mitigate potential bias arising from heterogeneous mechanisms of action and multicomponent combination therapies, the interventions were systematically classified according to their primary mechanism of action before the NMA. The classification scheme is detailed below. 1. Neuromodulation therapies: These were subdivided based on the target site and technical invasiveness: (i) Minimally invasive peripheral nerve modulation: Low-invasiveness techniques targeting peripheral nerves (e.g., pulsed radiofrequency therapy). (ii) Minimally invasive central nervous system neuromodulation: Moderately invasive techniques targeting the spinal cord (e.g., short-term spinal cord stimulation). (iii) Targeted peripheral nerve electrical stimulation: Precise stimulation of specific peripheral nerves (e.g., short-term supraorbital nerve stimulation). (iv) Superficial electrical neuromodulation: Non-invasive transcutaneous electrical stimulation techniques (e.g., transcutaneous electrical nerve stimulation). (v) Non-invasive central nervous system neuromodulation: Non-invasive techniques targeting the brain (e.g., repetitive transcranial magnetic stimulation). 2. Pharmacological and chemical therapies: This category was differentiated by the mechanism of action and route of administration: (i) Topical and peripheral chemical interventions: Locally administered agents (e.g., Botulinum Toxin A subcutaneous injection, lidocaine intradermal injection, steroid/local anesthetic compound preparation). (ii) Systemic pharmacological analgesia: Systemically administered drugs (e.g., Hydromorphone intravenous patient-controlled analgesia). (iii) Chemical selective neurolysis: Neurodestructive procedures (e.g., dorsal root ganglion destruction by Adriamycin). 3. Energy and physical therapies: These interventions were classified according to the mechanism of action of exogenous physical energy: (i) Physical therapy and energy medicine: Modalities utilizing physical energy (e.g., extracorporeal shock wave therapy, pulsed electromagnetic field therapy, light-emitting diode therapy). (ii) Medical oxidant therapy: Approaches based on redox reactions (e.g., ozone autohemotherapy). 4. Biological therapy: This modality aimed to achieve therapeutic effects by modulating intrinsic biological processes, such as immune responses or tissue repair (e.g., BCG polysaccharide and nucleic acid injection, platelet-rich plasma injection, autologous fat grafting). 5. Nerve block: Regional nerve blockade techniques (e.g., stellate ganglion block, erector spinae plane block, paravertebral block). 6. Conventional and alternative medicine: (i) Standard pharmacotherapy: First-line oral medications (e.g., pregabalin, gabapentin). (ii) Complementary and alternative medicine: Mind–body interventions (e.g., mindfulness meditation). 7. Sham control: Placebo interventions simulating authentic treatment procedures. 8. Combination therapy: Original combination formats were preserved (e.g., pulsed radiofrequency + nerve block, spinal cord stimulation + lidocaine patch). Table 1 summarizes the baseline characteristics of the included studies.

**Table 1:** Baseline characteristics of the included studies (serves to assess the transitivity assumption of the network meta-analysis by demonstrating the distributional balance of potential effect modifiers across the different intervention groups).

Authors	Year	Study design	Area	Intervention groups	Description of interventions	Sample size	Female, *n* (%)	Ages	Follow-up duration	Disease stage	Lesion site	Baseline pain scores	Outcomes
Y. Li et al. (25)	2025	RCT	China	Minimally invasive central nervous system neuromodulation + Topical and peripheral chemical interventions	Short-term spinal cord stimulation + Lidocaine patch	49	25 (51%)	63.84 ± 10.20	3 M	Postherpetic neuralgia	Truncal	7.20 ± 1.40	VAS, PSQI
				Minimally invasive central nervous system neuromodulation	Short-term spinal cord stimulation + Placebo patch	48	25 (52%)	63.70 ± 3.10	24 M			7.10 ± 1.30	
Y. Li et al. (26)	2025	RCT	China	Minimally invasive central nervous system neuromodulation	Short-term Spinal Cord Stimulation	70	46 (66%)	63.00 ± 4.30	6 M	Postherpetic neuralgia	Cervical, Truncal	7.35 ± 0.90	VAS, PSQI, SAS, SDS
				Minimally invasive peripheral nerve modulation	Bipolar pulsed radiofrequency	70	44 (63%)	61.33 ± 7.96	2 M			7.40 ± 0.90	
C. Wu et al. (24)	2025	RCT	China	Targeted peripheral nerve electrical stimulation	Short-term supraorbital nerve stimulation	31	15 (48%)	59.00 ± 7.60	0.5 M	Acute herpes zoster pain	Cranial	7.26 ± 1.03	VAS, Adverse events
				Minimally invasive peripheral nerve modulation	Supraorbital pulsed radiofrequency	34	14 (41%)	/	6 M			7.00 ± 1.07	
Y. Wu et al. (23)	2025	RCT	China	Biological therapy	BCG polysaccharide and nucleic acid injection	49	25 (51%)	/	6 M	Postherpetic neuralgia	/	6.89 ± 1.82	VAS, Adverse events
				Standard treatment	Standard oral therapy	49	26 (53%)	56.80 ± 3.10	2 M			7.03 ± 1.68	
C. Zheng et al. (22)	2025	RCT	China	Standard treatment	Standard oral therapy	40	22 (55%)	56.20 ± 3.70	1 M	Postherpetic neuralgia	Cranial	4.36 ± 2.32	VAS, Adverse events
				Nerve block	Stellate ganglion block	52	26 (50%)	53.14 ± 4.64	6 M			4.00 ± 1.52	
				Medical oxidant therapy	Ozone autohemotherapy	53	28 (53%)	54.50 ± 4.85	1 M			4.00 ± 1.52	
				Medical oxidant therapy + Nerve block	Ozone autohemotherapy + Stellate ganglion block	45	21 (47%)	73.14 ± 6.64	6 M			4.35 ± 2.30	
Z. Zhou et al. (27)	2024	RCT	China	Biological therapy	Platelet-rich plasma injection	40	17 (43%)	71.18 ± 7.30	6 M	Acute herpes zoster pain	Cervical, Truncal	5.80 ± 0.60	NRS
				Standard treatment	Standard oral therapy	40	19 (48%)	73.20 ± 10.50	3 M			5.90 ± 0.40	
R. Wang et al. (28)	2024	RCT	China	Minimally invasive peripheral nerve modulation + Medical oxidant therapy	Pulsed radiofrequency + Dorsal root ganglion ozone injection	81	47 (58%)	77.50 ± 8.20	2 M	Acute herpes zoster pain	/	6.06 ± 0.64	NRS
				Minimally invasive peripheral nerve modulation	Pulsed radiofrequency	83	47 (57%)	66.82 ± 7.78	1.5 M			6.10 ± 0.63	
A. Patil et al. (29)	2024	RCT	India	Nerve block	Erector spinae plane block	20	13 (65%)	66.75 ± 10.98	12 M	Acute herpes zoster pain	Truncal	7.70 ± 1.12	NRS, Adverse events
				Nerve block	Paravertebral block	20	9 (45%)	67.21 ± 9.37	6 M			8.00 ± 1.17	
Y. Liu et al. (30)	2024	RCT	China	Standard treatment	Standard oral therapy	25	11 (44%)	60.60 ± 2.20	6 M			7.50 ± 1.19	VAS, PSQI, Adverse events
				Targeted peripheral nerve electrical stimulation	Short-term supraorbital nerve stimulation	25	12 (48%)	59.60 ± 3.20	2 M	Acute herpes zoster pain, Subacute herpes zoster pain	Cranial	7.69 ± 0.46	
S. Lin et al. (31)	2024	RCT	China	Nerve block	Supraorbital nerve block	30	12 (40%)	69.20 ± 11.80	6 M			7.41 ± 0.41	VAS, PSQI, Adverse events
				Minimally invasive peripheral nerve modulation	Pulsed radiofrequency	30	14 (47%)	68.40 ± 12.30	3 M	Subacute herpes zoster pain	Cranial, Cervical	7.10 ± 1.60	
Z.-W. Zhang et al. (32)	2023	RCT	China	Sham treatment	Sham treatment	30	16 (53%)	65.47 ± 4.49	6 M			6.90 ± 1.80	VAS, PSQI, Adverse events
				Minimally invasive peripheral nerve modulation + Nerve block	Pulsed radiofrequency + Spinal nerve block	30	17 (57%)	67.42 ± 5.93	6 M	Acute herpes zoster pain, Subacute herpes zoster pain	Truncal	7.32 ± 2.31	
				Physically-ablative neuromodulation + Nerve block	Low-temperature plasma ablation of the DRG + Spinal nerve block	30	12 (40%)	65.97 ± 2.97	3 M			7.32 ± 2.31	
H. Sun et al. (33)	2023	RCT	China	Nerve block	Spinal nerve block	82	32 (39%)	66.02 ± 2.97	3 M			7.11 ± 2.98	VAS
				Physical therapy and energy medicine	Extracorporeal shock wave treatment	82	28 (34%)	63.84 ± 10.20	3 M	Postherpetic neuralgia	Cranial, Cervical, Truncal	7.17 ± 0.49	
M. Sollie et al. (34)	2023	RCT	Denmark	Standard treatment	Standard oral therapy	23	/	63.70 ± 3.10	3 M			7.22 ± 0.47	NRS
				Biological therapy	Autologous fat grafting	23	/	63.00 ± 4.30	1 M	Postherpetic neuralgia	/	7.40 ± 0.40	
S. Lin et al. (35)	2023	RCT	China	Sham treatment	Sham treatment	30	12 (40%)	61.33 ± 7.96	3 M			7.30 ± 0.40	VAS, PSQI
				Minimally invasive peripheral nerve modulation	Bipolar high-voltage pulsed radiofrequency	30	14 (47%)	59.00 ± 7.60	6 M	Acute herpes zoster pain	Cranial, Cervical, Upper Limbs	7.10 ± 1.60	
W. Zhang et al. (36)	2022	RCT	China	Sham treatment	Sham treatment	32	17 (53%)	/	6 M			6.90 ± 1.80	VAS, PSQI, adverse events
				Minimally invasive peripheral nerve modulation + Systemic pharmacological analgesia	Pulsed radiofrequency + Intravenous lidocaine infusion	32	14 (44%)	/	1 M	Subacute herpes zoster pain	/	7.46 ± 1.07	
C. Wang et al. (37)	2022	RCT	China	Minimally invasive peripheral nerve modulation	Pulsed radiofrequency	12	/	56.80 ± 3.10	6 M			7.21 ± 1.15	VAS
				Nerve block	Stellate ganglion block	12	/	56.20 ± 3.70	2 M	Postherpetic neuralgia	/	7.80 ± 1.30		
				Physical therapy and energy medicine	Extracorporeal shock wave therapy	12	/	53.14 ± 4.64	1 M						7.90 ± 0.90	
L. Sheng et al. (38)	2022	RCT	China	Physical therapy and energy medicine + Nerve block	Extracorporeal shock wave therapy + Stellate ganglion block	38	19 (50%)	54.50 ± 4.85	12 M					7.80 ± 1.00	VAS, Adverse events
				Minimally invasive peripheral nerve modulation	Pulsed radiofrequency	29	14 (48%)	73.14 ± 6.64	/	Postherpetic neuralgia	Cervical, Truncal	6.66 ± 1.81			
X. Li et al. (39)	2022	RCT	China	Minimally invasive central nervous system neuromodulation	Short-term spinal cord stimulation	20	9 (45%)	71.18 ± 7.30	/					7.21 ± 1.78	VAS, SIS
				Minimally invasive central nervous system neuromodulation	Short-term spinal cord stimulation	20	10 (50%)	73.20 ± 10.50	/	Postherpetic neuralgia	Cervical, Truncal, Upper Limbs, Lower Limbs	8.11 ± 0.24			
M. Ji et al. (40)	2022	RCT	China	Minimally invasive peripheral nerve modulation	Pulsed radiofrequency	36	16 (44%)	77.50 ± 8.20	/					8.29 ± 0.96	VAS, ISI
				Minimally invasive peripheral nerve modulation + Nerve block	Pulsed radiofrequency + Methylene blue paravertebral nerve block	36	18 (50%)	66.82 ± 7.78	3 M	Postherpetic neuralgia	/	6.50 ± 0.81			
M. M. Eid et al. (41)	2022	RCT	Egypt	Minimally invasive peripheral nerve modulation	Pulsed radiofrequency	28	20 (71%)	66.75 ± 10.98	6 M					6.50 ± 0.74	VAS
				Superficial electrical neuromodulation	Transcutaneous electrical nerve stimulation	28	18 (64%)	67.21 ± 9.37	6 M	Postherpetic neuralgia	/	8.71 ± 0.93			
L. Chen et al. (42)	2022	RCT	China	Physical therapy and energy medicine	Pulsed electromagnetic field therapy	50	24 (48%)	60.60 ± 2.20	6 M					8.60 ± 0.78	NRS, SQS, Adverse events
				Minimally invasive peripheral nerve modulation	Pulsed radiofrequency	50	24 (48%)	59.60 ± 3.20	2 M	Postherpetic neuralgia	/	6.00 ± 1.01			
L. Chen et al. (43)	2022	RCT	China	Topical and peripheral chemical interventions	Botulinum Toxin A subcutaneous injection	34	14 (41%)	69.20 ± 11.80	2 M					6.12 ± 1.10	NRS, PSQI, Adverse events
				Physical therapy and energy medicine	Extracorporeal shockwave therapy	35	20 (57%)	68.40 ± 12.30	6 M	Postherpetic neuralgia	Multiple sites	7.18 ± 1.35			
E. H. Abdelwahab et al. (44)	2022	RCT	Egypt	Standard treatment	Standard oral therapy	30	16 (53%)	65.47 ± 4.49						7.62 ± 1.35	NRS
				Nerve block	Erector spinae plane block	30	17 (57%)	67.42 ± 5.93		Postherpetic neuralgia	Truncal	7.00 ± 1.56			
C. F. Wan et al. (45)	2021	RCT	China	Nerve block	Thoracic paravertebral block	46	26 (57%)	65.97 ± 2.97	6 M					7.36 ± 2.44	NRS, Adverse events
				Standard treatment	Standard oral therapy	45	24 (53%)	66.02 ± 2.97	1 M					7.00 ± 1.56		
A. K. Saxena et al. (46)	2021	RCT	India	Minimally invasive peripheral nerve modulation	Pulsed radiofrequency	20	/	63.84 ± 10.20	1 M	Acute herpes zoster pain, Subacute herpes zoster pain	Cervical, Truncal	7.48 ± 2.91	VAS, NRS-Sleep
				Minimally invasive central nervous system neuromodulation	Short-term spinal cord stimulation	20	/	63.70 ± 3.10	6 M			7.39 ± 2.73	
J. Liu et al. (47)	2021	RCT	China	Minimally invasive peripheral nerve modulation	Pulsed radiofrequency	80	36 (45%)	63.00 ± 4.30	6 M	Postherpetic neuralgia	Truncal	6.80 ± 1.50	VAS, PSQI
				Standard treatment	Standard oral therapy	80	38 (48%)	61.33 ± 7.96	12 M			5.90 ± 1.10	
Y. Huang et al. (48)	2021	RCT	China	Minimally invasive central nervous system neuromodulation	Short-term spinal cord stimulation	96	50 (52%)	59.00 ± 7.60	6 M	Postherpetic neuralgia	Cervical, Truncal, Upper Limbs, Lower Limbs	7.39 ± 0.88	NRS, PSQI, Adverse events
				Nerve block	Nerve block	97	46 (47%)	/	3 M			7.48 ± 0.82	
M. A. El-Sayed et al. (49)	2021	RCT	Egypt	Systemic pharmacological analgesia	Hydromorphone intravenous patient-controlled analgesia	20	/	/	3 M	Postherpetic neuralgia	Cranial, Cervical, Truncal, Lower Limbs	6.90 ± 1.50	VAS
				Sham treatment	Sham treatment	20	/	56.80 ± 3.10	3 M			6.50 ± 1.60	
X. Dong et al. (50)	2021	RCT	China	Nerve block	Erector spinae plane block	48	20 (42%)	56.20 ± 3.70	1 M	Acute herpes zoster pain	Truncal	8.89 ± 1.13	VAS, Adverse events
				Standard treatment	Standard oral therapy	48	22 (46%)	53.14 ± 4.64	1 M			9.05 ± 1.52	
Z.-H. Xiong et al. (51)	2020	RCT	China	Nerve block	Continuous epidural block	39	20 (51%)	54.50 ± 4.85	3 M	Postherpetic neuralgia	Truncal, Upper Limbs, Lower Limbs	9.00 ± 0.85	NRS, Sleep disturbance scores
				Standard treatment	Standard oral therapy	39	22 (56%)	73.14 ± 6.64	3 M			9.00 ± 0.73	
B. Liu et al. (52)	2020	RCT	China	Minimally invasive peripheral nerve modulation	Pulsed radiofrequency	31	18 (58%)	71.18 ± 7.30	6 M	Postherpetic neuralgia	Truncal	7.50 ± 1.40	NRS, Adverse events
				Standard treatment	Standard oral therapy	32	14 (44%)	73.20 ± 10.50	6 M			7.60 ± 1.20	
S. Zheng et al. (53)	2019	RCT	China	Minimally invasive central nervous system neuromodulation	Short-term spinal cord stimulation	70	41 (59%)	77.50 ± 8.20	6 M	Subacute herpes zoster pain, Postherpetic neuralgia	Cervical, Truncal, Upper Limbs, Lower Limbs	8.13 ± 1.07	ZBPI
				Minimally invasive peripheral nerve modulation	Pulsed radiofrequency	70	39 (56%)	66.82 ± 7.78	6 M			7.73 ± 1.31	
P. Zhao et al. (54)	2019	RCT	China	Nerve block	Cervical nerve root block	43	20 (47%)	66.75 ± 10.98	1 M	Acute herpes zoster pain	Upper Limbs	7.37 ± 1.76	VAS, QS
				Sham treatment	Sham treatment	44	21 (48%)	67.21 ± 9.37	1 M			7.40 ± 1.75	
C. Wang et al. (55)	2019	RCT	China	Nerve block	Paraspinal nerve block	46	21 (46%)	60.60 ± 2.20	6 M	Subacute herpes zoster pain	/	7.61 ± 0.53	VAS
				Standard treatment	Standard oral therapy	47	20 (43%)	59.60 ± 3.20	6 M			7.59 ± 0.75	
Q. Pei et al. (56)	2019	RCT	China	Minimally invasive peripheral nerve modulation	Pulsed radiofrequency	20	11 (55%)	69.20 ± 11.80	2 M	Acute herpes zoster pain, Subacute herpes zoster pain	Cranial	7.32 ± 2.33	VAS, SQ
				Sham treatment	Sham treatment	20	9 (45%)	68.40 ± 12.30	2 M			7.31 ± 2.39	
J. Zhang et al. (57)	2018	RCT	China	Non-invasive central nervous system neuromodulation	5-Hz Repetitive transcranial magnetic stimulation	25	15 (60%)	65.47 ± 4.49	1 M	Postherpetic neuralgia	/	6.90 ± 1.10	VAS, SIS, Adverse events
				Non-invasive central nervous system neuromodulation	10-Hz Repetitive transcranial magnetic stimulation	25	13 (52%)	67.42 ± 5.93	1 M			6.30 ± 1.70	
M. Y. Makharita et al. (58)	2018	RCT	Egypt	Sham treatment	Sham treatment	21	9 (43%)	65.97 ± 2.97	12 M			6.80 ± 1.60	VAS, Adverse events
				Nerve block	Epidural block of lidocaine	22	10 (45%)	66.02 ± 2.97	12 M	Postherpetic neuralgia	Truncal	8.07 ± 0.97	
D. Li et al. (59)	2018	RCT	China	Topical and peripheral chemical interventions	Intradermal injection of lidocaine	15	/	63.84 ± 10.20	/			8.14 ± 0.56	VAS
				Minimally invasive peripheral nerve modulation	Pulsed radiofrequency	15	/	63.70 ± 3.10	/	Postherpetic neuralgia	Truncal	7.31 ± 1.25	
				Sham treatment	Sham treatment	15	/	63.00 ± 4.30	/			7.23 ± 1.29	
				Sham treatment	Sham treatment	15	/	61.33 ± 7.96	/	Postherpetic neuralgia	Cervical, Truncal	7.73 ± 0.88	
B. Hu et al. (60)	2018	RCT	China	Nerve block	Nerve block	46	18 (39%)	59.00 ± 7.60	3 M			8.07 ± 0.96	VAS
				Minimally invasive peripheral nerve modulation	Pulsed radiofrequency	45	20 (44%)	/	3 M			7.80 ± 0.94	
J.-z. Cui et al. (61)	2018	RCT	China	Minimally invasive peripheral nerve modulation + Nerve block	Pulsed radiofrequency + Nerve block	49	28 (57%)	/	6 M			7.80 ± 0.86	VAS
				Medical oxidant therapy	Ozone autohemotherapy	48	28 (58%)	56.80 ± 3.10	6 M	Postherpetic neuralgia	Cranial, Cervical, Truncal, Upper Limbs	6.50 ± 1.40	
J. Ni et al. (62)	2017	RCT	China	Standard treatment	Standard oral therapy	50	26 (52%)	56.20 ± 3.70	6 M			6.20 ± 1.30	NRS
				Topical and peripheral chemical interventions	Intracutaneous injection of ropivacaine plus methylprednisolone	50	27 (54%)	53.14 ± 4.64	6 M	Acute herpes zoster pain	Truncal	7.00 ± 1.20	
J. Z. Cui et al. (63)	2017	RCT	China	Sham treatment	Sham treatment	47	26 (55%)	54.50 ± 4.85	6 M			7.10 ± 1.40	VAS
				Topical and peripheral chemical interventions	Subcutaneous injection of triamcinolone and lidocaine	46	27 (59%)	73.14 ± 6.64	6 M	Acute herpes zoster pain	Cranial, Cervical, Truncal	7.16 ± 1.22	
A. K. Saxena et al. (64)	2016	RCT	India	Standard treatment	Standard oral therapy	30	13 (43%)	71.18 ± 7.30	2 M			6.64 ± 1.44	VAS, NRS-sleep
				Topical and peripheral chemical interventions	Repetitive intracutaneous injections of local anesthetic plus steroid	30	13 (43%)	73.20 ± 10.50	2 M	Acute herpes zoster pain	Truncal	7.50 ± 1.70	
R. Meize-Grochowski et al. (65)	2015	RCT	USA	Standard treatment	Standard oral therapy	13	6 (46%)	77.50 ± 8.20	2 M			7.50 ± 1.40	SF-MPQ-2
				Minimally invasive peripheral nerve modulation	Pulsed radiofrequency	14	9 (64%)	66.82 ± 7.78	2 M	Postherpetic neuralgia	Truncal	6.87 ± 0.73	
M. Y. Makharita et al. (66)	2015	RCT	Egypt	Sham treatment	Sham treatment	70	36 (51%)	66.75 ± 10.98	6 M			7.10 ± 0.80	VAS, Adverse events
				Complementary and alternative medicine	Mindfulness meditation	68	37 (54%)	67.21 ± 9.37	6 M	Postherpetic neuralgia	Multiple sites	3.50 ± 2.20	
K. Y. Park et al. (68)	2013	RCT	Korea	Standard treatment	Usual care	14	7 (50%)	60.60 ± 2.20	/			2.40 ± 1.50	VAS, Adverse events
				Nerve block	Paravertebral block	14	6 (43%)	59.60 ± 3.20	/	Acute herpes zoster pain, Subacute herpes zoster pain	Truncal	7.37 ± 1.57	
K. Ma et al. (69)	2013	RCT	China	Sham treatment	Sham treatment	48	23 (48%)	69.20 ± 11.80	6 M			7.34 ± 1.17	VAS, Adverse events
				Physical therapy and energy medicine	830 nm Light-emitting diode therapy	48	26 (54%)	68.40 ± 12.30	6 M	Acute herpes zoster pain	Cranial, Cervical	6.78 ± 0.87	
Z. Apalla et al. (70)	2013	RCT	Greece	Standard treatment	Standard oral therapy	15	7 (47%)	65.47 ± 4.49	1 M			6.64 ± 0.69	VAS, 5-Item Sleep Questionnaire Score, Adverse events
				Minimally invasive peripheral nerve modulation	Pulsed Radiofrequency	15	5 (33%)	67.42 ± 5.93	1 M	Postherpetic neuralgia	Truncal	6.10 ± 0.08	
G. Xu et al. (67)	2013	RCT	China	Sham treatment	Sham treatment	33	18 (55%)	65.97 ± 2.97	1 M			6.36 ± 0.09	NRS
				Topical and peripheral chemical interventions	Botulinum Toxin A local injection			66.02 ± 2.97		Postherpetic neuralgia	Truncal, Upper Limbs, Lower Limbs	8.80 ± 1.00	
				Sham treatment	Sham treatment	33	16 (48%)	63.84 ± 10.20	1 M			8.70 ± 0.80	
M. Y. Makharita et al. (71)	2012	RCT	Egypt	Topical and peripheral chemical interventions	Local methylcobalamin injection	31	18 (58%)	63.70 ± 3.10	6 M	Subacute herpes zoster pain	Truncal	6.90 ± 1.50	VAS
				Topical and peripheral chemical interventions	Subcutaneous lidocaine injection	30	16 (53%)	63.00 ± 4.30	6 M			7.10 ± 1.60	
C.-j. He et al. (72)	2012	RCT	China	Standard treatment	Standard oral therapy	36	25 (69%)	61.33 ± 7.96	6 M			6.90 ± 1.10	VAS, Adverse events
				Nerve block	Stellate ganglion block	36	22 (61%)	59.00 ± 7.60	6 M	Acute herpes zoster pain	Cranial	7.00 ± 0.90	
G. Ji et al. (73)	2009	RCT	China	Sham treatment	Sham treatment	64	36 (56%)	/	12 M			7.10 ± 1.10	VAS, Adverse events
				Chemical selective neurolysis	Dorsal root ganglion destruction by adriamycin	68	38 (56%)	/	12 M	Postherpetic neuralgia	Truncal	7.64 ± 1.19	
A. J. van Wijck et al. (74)	2006	RCT	Netherlands	Nerve block	Transforaminal injection of dexamethasone plus lidocain	301	183 (61%)	56.80 ± 3.10	6 M			7.55 ± 1.44	VAS, Adverse events
				Nerve block	Repetitive paravertebral injections of local anesthetics and steroids	297	181 (61%)	56.20 ± 3.70	6 M	Acute herpes zoster pain	Cervical, Truncal, Upper Limbs, Lower Limbs	7.59 ± 1.07	

**Figure 2:**
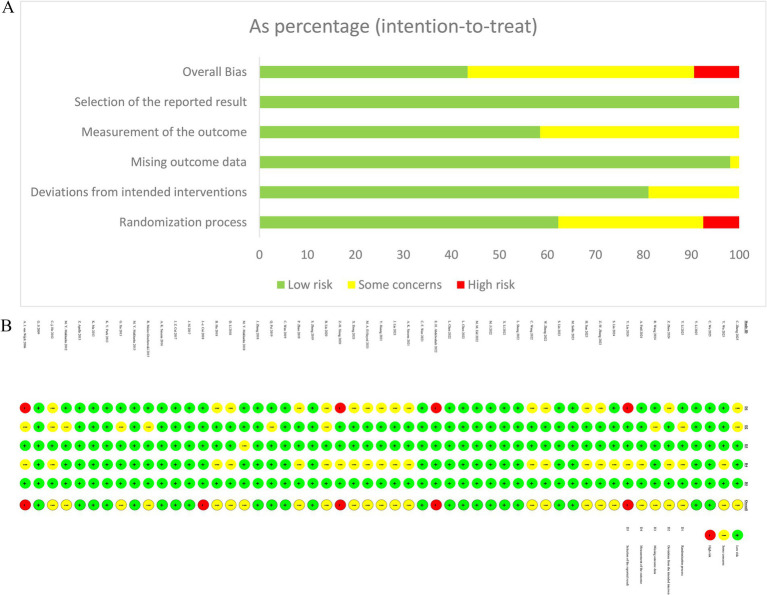
Bias assessment graph. **(A)** Summary chart of bias risk of included studies. **(B)** Risk of bias for each included study.

The risk of bias assessment for the included studies is illustrated in Figure 2. Of the 53 studies included, 23 (43.4%) were judged as low risk, 25 (47.2%) raised some concerns, and five (9.4%) were rated as high risk of bias. The main reasons for the ‘some concerns’ or ‘high risk’ ratings, analyzed by assessment domain, are shown below. In Domain 1 (D1: Bias arising from the randomization process), four studies received a high-risk rating due to an insufficient description of both random sequence generation and allocation concealment. An additional 12 studies were assessed as having ‘some concerns’ due to inadequate reporting of randomization-related details. Domain 2 (D2: Bias due to deviations from intended interventions) presented inherent methodological limitations. Fundamental differences in the nature of various interventions (e.g., comparisons between invasive procedures, such as neuromodulation and nerve blocks, and standard oral pharmacotherapy) precluded blinding of participants and study personnel in several studies. Regarding Domain 3 (D3: Bias due to missing outcome data), one study raised concerns due to a high attrition rate (> 10%) and the absence of an intention-to-treat analysis. The remaining studies exhibited a low risk given the minimal, balanced missing data across groups. For Domain 4 (D4: Bias in measurement of the outcome), the primary outcome (pain intensity) was patient-reported. In studies where participant blinding was not feasible, the potential for subjective expectation to influence reporting was a recognized source of bias. This is common in interventional research utilizing subjective endpoints. All studies were rated as low risk in Domain 5 (D5: Bias in selection of the reported result), indicating a low likelihood of selective outcome reporting. In summary, despite limitations regarding blinding and reporting randomization procedures, the overall risk of bias for most included studies remained within an acceptable range.

### 3.3 Results of the NMA

#### 3.3.1 Network characteristics and model assessment

**Figure 3:**
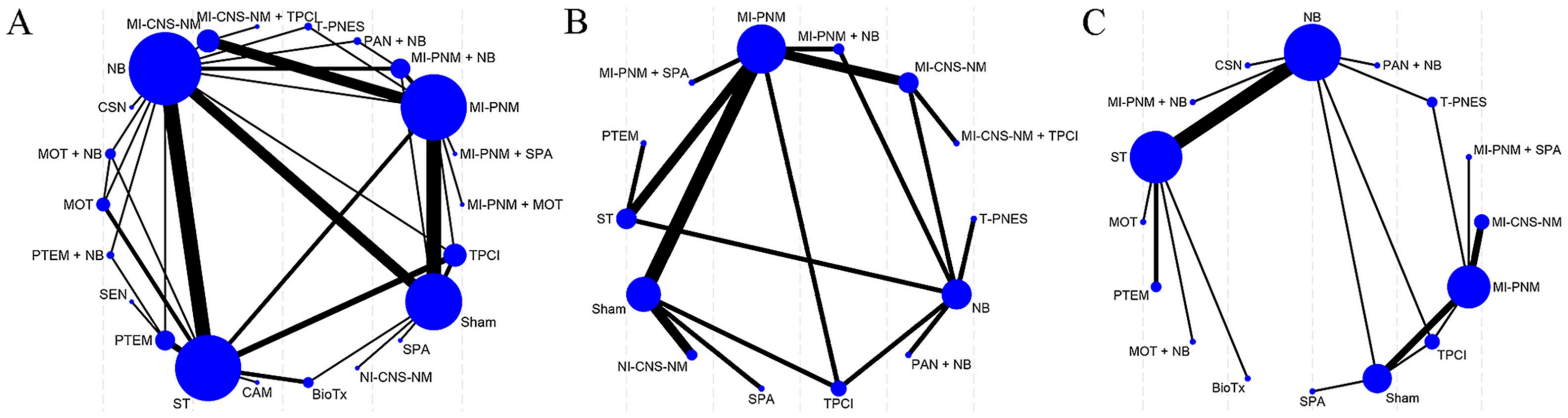
Network evidence diagram: **(A)** pain relief; **(B)** sleep quality improvement; **(C)** adverse events.

In the network diagram, each node corresponded to a distinct intervention, and the size of each node was proportional to the number of studies evaluating that intervention. Lines connecting nodes indicated the presence of direct comparative evidence. The thickness of the lines was proportional to the number of studies contributing to each direct comparison (Figure 3).

Evaluation of local heterogeneity revealed differential levels across the three outcomes. For the primary outcome (pain relief), most direct comparisons exhibited moderate to high heterogeneity (I^2^ ≥ 50%). Conversely, comparisons for the sleep quality and AEs were predominantly characterized by low heterogeneity (I^2^ < 50%). For all outcomes, certain comparisons lacked I^2^ calculations due to the inclusion of only one study (Supplementary Figures S1–S3).

Assessment of local inconsistency via the node-splitting method detected no significant divergence between direct and indirect evidence for any comparison related to pain relief, sleep quality, or AEs (all *p* > 0.05) (Supplementary Figures S4–S6). Global consistency was further evaluated by comparing the goodness-of-fit between the consistency and node-splitting inconsistency models. For both pain intensity (ΔDIC = −0.336) and sleep quality (ΔDIC = −0.396), the minimal absolute ΔDIC values were negative, indicating a superior fit for the consistency model and strongly supporting global consistency across the network. Regarding AEs, the value below five (ΔDIC = 4.390) did not suggest compelling evidence for substantial global inconsistency (Supplementary Table S1).

All models demonstrated a PSRF approaching 1.00 (< 1.05), indicating sufficient convergence of the Markov chains (Supplementary Figures S7–S9). Trace plots illustrated excellent mixing between chains for all parameters, further validating convergence (Supplementary Figures S10–S12).

#### 3.3.2 Pooled results for each outcome measure

##### 3.3.2.1 Pain relief

The present NMA synthesized data from all 53 included studies that reported pain scores. The results demonstrated that several intervention strategies, whether used alone or in combination, produced statistically significant pain reduction compared to standard oral treatment alone. Interventions associated with significantly lower pain scores included nerve block (SMD = 0.79, 95% CrI: 0.19 to 1.39), minimally invasive peripheral nerve modulation (SMD = 1.13, 95% CrI: 0.32 to 1.93), minimally invasive central nervous system neuromodulation (SMD = 2.48, 95% CrI: 1.37 to 3.59), topical and peripheral chemical interventions (SMD = 1.13, 95% CrI: 0.33 to 1.93), physical therapy and energy medicine (SMD = 1.81, 95% CrI: 0.75 to 2.87), chemical selective neurolysis (SMD = 4.34, 95% CrI: 2.18 to 6.49), minimally invasive peripheral nerve modulation + nerve block (SMD = 2.01, 95% CrI: 0.65 to 3.37), minimally invasive peripheral nerve modulation + systemic pharmacological analgesia (SMD = 2.96, 95% CrI: 0.71 to 5.18), and minimally invasive central nervous system neuromodulation + topical and peripheral chemical interventions (SMD = 3.41, 95% CrI: 1.08 to 5.73) (Table 2).

**Table 2:** League table of pain relief outcomes.

**ST**																					
**0.79 (0.19, 1.39)**	**NB**																				
**1.13 (0.32, 1.93)**	0.34 (−0.43, 1.1)	**MI-PNM**																			
−0.05 (−0.82, 0.7)	**−0.84 (−1.5, −0.19)**	**−1.18 (−1.83, −0.53)**	**Sham**																		
**2.48 (1.37, 3.59)**	**1.7 (0.63, 2.75)**	**1.36 (0.5, 2.21)**	**2.53 (1.51, 3.56)**	**MI-CNS-NM**																	
**1.13 (0.33, 1.93)**	0.34 (−0.52, 1.21)	0 (−0.93, 0.95)	**1.18 (0.3, 2.08)**	**−1.36 (−2.57, −0.13)**	**TPCI**																
**1.81 (0.75, 2.87)**	1.02 (−0.12, 2.16)	0.68 (−0.61, 1.98)	**1.86 (0.61, 3.12)**	−0.68 (−2.17, 0.82)	0.68 (−0.62, 1.98)	**PTEM**															
0.9 (−1.27, 3.05)	0.11 (−2.03, 2.23)	−0.23 (−2.37, 1.89)	0.95 (−1.08, 2.97)	−1.59 (−3.87, 0.69)	−0.23 (−2.46, 1.97)	−0.91 (−3.3, 1.47)	**SPA**														
1.58 (−0.01, 3.17)	0.79 (−0.73, 2.31)	0.46 (−1.07, 1.97)	**1.63 (0.07, 3.2)**	−0.9 (−2.61, 0.82)	0.46 (−1.24, 2.13)	−0.22 (−2.09, 1.64)	0.69 (−1.88, 3.25)	**T-PNES**													
2.25 (−0.08, 4.57)	1.46 (−0.91, 3.82)	1.12 (−1.32, 3.57)	2.3 (−0.12, 4.73)	−0.24 (−2.79, 2.33)	1.12 (−1.34, 3.55)	0.44 (−1.64, 2.51)	1.35 (−1.8, 4.49)	0.67 (−2.13, 3.46)	**SEN**												
0.95 (−0.27, 2.18)	0.17 (−1.14, 1.47)	−0.17 (−1.55, 1.2)	1 (−0.3, 2.31)	−1.53 (−3.11, 0.04)	−0.17 (−1.59, 1.23)	−0.85 (−2.46, 0.75)	0.06 (−2.35, 2.48)	−0.63 (−2.58, 1.32)	−1.29 (−3.91, 1.33)	**BioTx**											
1.58 (−0.12, 3.26)	0.79 (−0.86, 2.43)	0.45 (−1.19, 2.09)	**1.63 (0.13, 3.13)**	−0.9 (−2.73, 0.91)	0.45 (−1.31, 2.19)	−0.23 (−2.2, 1.72)	0.68 (−1.83, 3.21)	0 (−2.17, 2.17)	−0.67 (−3.53, 2.18)	0.63 (−1.37, 2.62)	**NI-CNS-NM**										
0.66 (−0.84, 2.17)	−0.13 (−1.75, 1.5)	−0.47 (−2.16, 1.25)	0.71 (−0.96, 2.41)	−1.82 (−3.68, 0.05)	−0.47 (−2.18, 1.23)	−1.15 (−2.98, 0.7)	−0.24 (−2.86, 2.41)	−0.92 (−3.1, 1.27)	−1.59 (−4.36, 1.18)	−0.29 (−2.23, 1.65)	−0.92 (−3.17, 1.35)	**MOT**									
0.33 (−1.82, 2.48)	−0.46 (−2.68, 1.77)	−0.8 (−3.08, 1.49)	0.38 (−1.88, 2.66)	−2.16 (−4.56, 0.27)	−0.8 (−3.09, 1.48)	−1.48 (−3.86, 0.92)	−0.56 (−3.59, 2.47)	−1.25 (−3.9, 1.42)	−1.92 (−5.07, 1.25)	−0.62 (−3.09, 1.85)	−1.25 (−3.98, 1.47)	−0.33 (−2.93, 2.29)	**CAM**								
**4.34 (2.18, 6.49)**	**3.55 (1.49, 5.62)**	**3.21 (1.01, 5.41)**	**4.39 (2.23, 6.56)**	1.85 (−0.46, 4.17)	**3.21 (0.97, 5.44)**	**2.53 (0.16, 4.89)**	**3.44 (0.5, 6.4)**	**2.76 (0.19, 5.32)**	2.09 (−1.07, 5.23)	**3.39 (0.94, 5.81)**	**2.76 (0.13, 5.4)**	**3.68 (1.05, 6.3)**	**4.01 (0.96, 7.04)**	**CSN**							
**2.01 (0.65, 3.37)**	1.22 (−0.06, 2.51)	0.88 (−0.4, 2.17)	**2.06 (0.79, 3.34)**	−0.47 (−1.98, 1.05)	0.88 (−0.58, 2.33)	0.2 (−1.47, 1.89)	1.11 (−1.27, 3.52)	0.43 (−1.48, 2.34)	−0.24 (−2.91, 2.43)	1.06 (−0.69, 2.82)	0.43 (−1.54, 2.41)	1.35 (−0.68, 3.38)	1.68 (−0.85, 4.21)	−2.33 (−4.76, 0.1)	**MI-PNM + NB**						
1.59 (−0.57, 3.75)	0.8 (−1.27, 2.88)	0.46 (−1.74, 2.67)	1.64 (−0.52, 3.82)	−0.9 (−3.21, 1.43)	0.46 (−1.79, 2.7)	−0.21 (−2.6, 2.16)	0.7 (−2.28, 3.67)	0.01 (−2.56, 2.57)	−0.66 (−3.83, 2.5)	0.64 (−1.82, 3.07)	0.01 (−2.62, 2.67)	0.93 (−1.71, 3.55)	1.26 (−1.78, 4.3)	−2.75 (−5.68, 0.17)	−0.42 (−2.86, 2.01)	**PAN + NB**					
**2.96 (0.71, 5.18)**	2.17 (−0.05, 4.38)	1.83 (−0.27, 3.92)	**3.01 (0.82, 5.19)**	0.47 (−1.78, 2.73)	1.83 (−0.46, 4.11)	1.15 (−1.3, 3.6)	2.06 (−0.91, 5.04)	1.38 (−1.21, 3.95)	0.71 (−2.49, 3.91)	2 (−0.48, 4.51)	1.38 (−1.27, 4.02)	2.3 (−0.41, 4.98)	2.63 (−0.48, 5.7)	−1.38 (−4.41, 1.64)	0.95 (−1.51, 3.39)	1.37 (−1.68, 4.4)	**MI-PNM + SPA**				
1.52 (−0.66, 3.7)	0.74 (−1.43, 2.89)	0.4 (−1.62, 2.43)	1.58 (−0.55, 3.71)	−0.96 (−3.16, 1.25)	0.4 (−1.85, 2.62)	−0.28 (−2.69, 2.11)	0.63 (−2.32, 3.57)	−0.06 (−2.58, 2.48)	−0.72 (−3.9, 2.44)	0.57 (−1.88, 3.01)	−0.06 (−2.65, 2.56)	0.87 (−1.79, 3.5)	1.19 (−1.86, 4.25)	−2.82 (−5.8, 0.18)	−0.49 (−2.88, 1.91)	−0.07 (−3.07, 2.92)	−1.43 (−4.35, 1.48)	**MI-PNM + MOT**			
**3.41 (1.08, 5.73)**	**2.62 (0.31, 4.92)**	**2.28 (0.06, 4.5)**	**3.46 (1.17, 5.75)**	0.92 (−1.13, 2.97)	2.28 (−0.11, 4.65)	1.6 (−0.93, 4.14)	2.51 (−0.54, 5.57)	1.83 (−0.85, 4.49)	1.16 (−2.11, 4.43)	2.46 (−0.13, 5.03)	1.83 (−0.9, 4.57)	2.75 (−0.03, 5.5)	3.08 (−0.08, 6.22)	−0.93 (−4.03, 2.16)	1.4 (−1.15, 3.93)	1.82 (−1.28, 4.91)	0.44 (−2.59, 3.52)	1.89 (−1.12, 4.89)	**MI-CNS-NM + TPCI**		
0.8 (−1.25, 2.87)	0.02 (−2.12, 2.16)	−0.32 (−2.53, 1.89)	0.86 (−1.33, 3.06)	−1.68 (−4.01, 0.67)	−0.32 (−2.53, 1.88)	−1 (−3.31, 1.31)	−0.09 (−3.06, 2.92)	−0.78 (−3.37, 1.82)	−1.45 (−4.55, 1.65)	−0.15 (−2.55, 2.25)	−0.78 (−3.44, 1.91)	0.15 (−2.41, 2.69)	0.48 (−2.48, 3.44)	**−3.53 (−6.51, −0.56)**	−1.21 (−3.66, 1.27)	−0.78 (−3.76, 2.21)	−2.15 (−5.19, 0.89)	−0.72 (−3.71, 2.29)	−2.6 (−5.7, 0.49)	**MOT + NB**	
1.86 (−0.39, 4.11)	1.07 (−1.1, 3.26)	0.73 (−1.57, 3.04)	1.91 (−0.35, 4.2)	−0.63 (−3.04, 1.8)	0.73 (−1.6, 3.07)	0.05 (−2.4, 2.51)	0.96 (−2.08, 4.01)	0.28 (−2.37, 2.93)	−0.39 (−3.61, 2.83)	0.9 (−1.63, 3.46)	0.28 (−2.44, 3.01)	1.19 (−1.52, 3.9)	1.53 (−1.6, 4.62)	−2.48 (−5.48, 0.53)	−0.16 (−2.68, 2.38)	0.27 (−2.73, 3.29)	−1.1 (−4.2, 2.02)	0.33 (−2.72, 3.42)	−1.55 (−4.7, 1.63)	1.05 (−1.99, 4.1)	**PTEM + NB**

Values represent the standardized mean difference (SMD) and 95% credible interval (CrI) for the comparison of the row intervention versus the column intervention in terms of pain relief outcomes. A positive SMD indicates that the row intervention is superior to the column intervention, while a negative SMD indicates the opposite. Statistically significant comparisons (95% CrI excluding 0) are presented in boldface. Full intervention names corresponding to the abbreviations used can be found in Supplementary Table S5.

**Figure 4:**
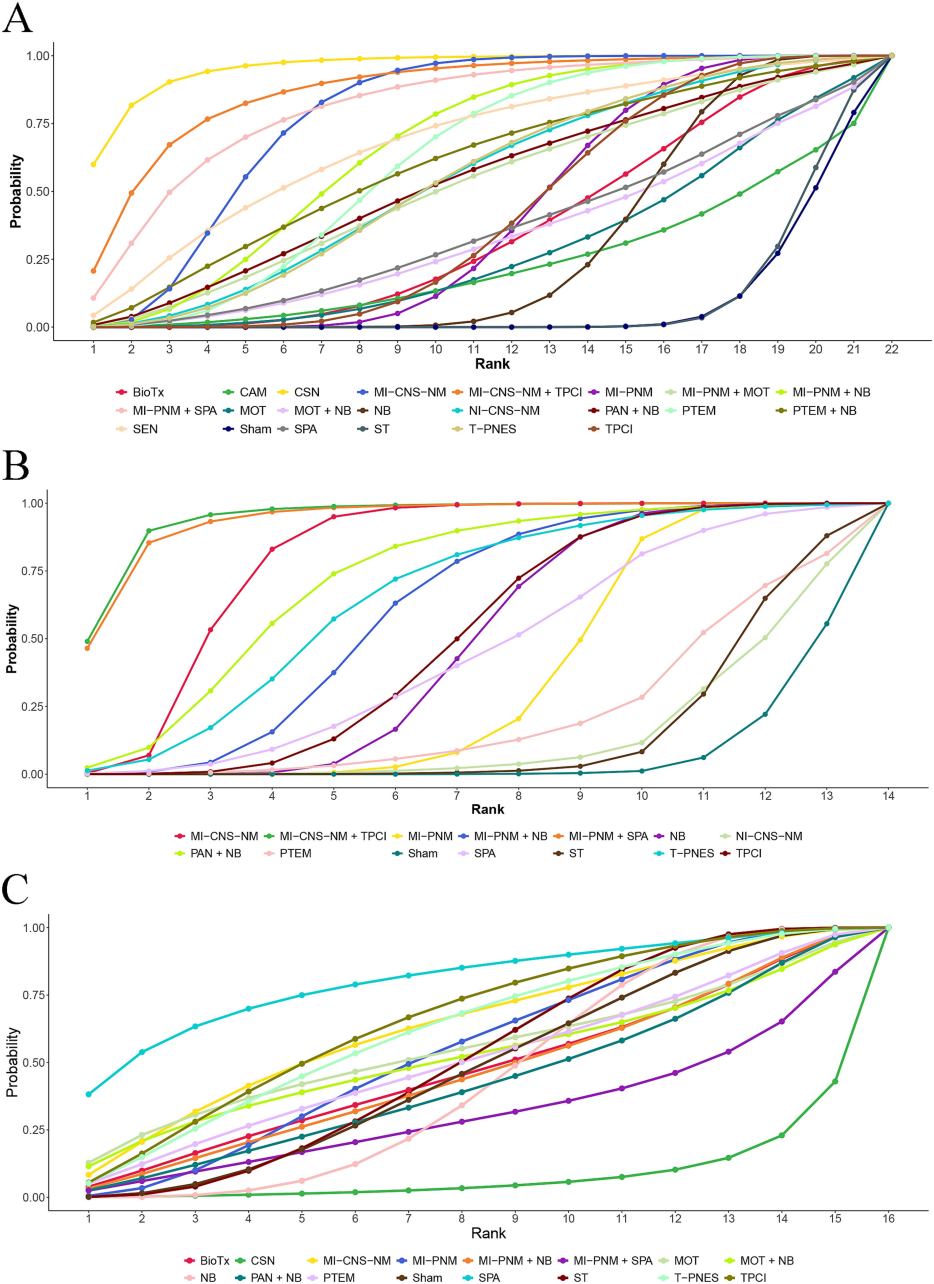
Rankograms of cumulative ranking probabilities. **(A)** Pain relief. The larger the area under each curve, the better the effect of treating and relieving pain. **(B)** Sleep quality improvement. The larger the area under each curve, the better the effect on improving sleep quality. **(C)** Adverse events. The larger the area under each curve, the lower the incidence of adverse reactions.

Based on the SUCRA values, chemical selective neurolysis had the highest probability (95.96%) of being the most effective analgesic strategy. The next highest probabilities were for minimally invasive central nervous system neuromodulation + topical and peripheral chemical interventions (87.63%) and minimally invasive peripheral nerve modulation + systemic pharmacological analgesia (81.79%) (Figure 4A).

##### 3.3.2.2 Sleep quality

Twenty studies reported on sleep quality outcomes. The NMA showed that several intervention strategies, whether used alone or in combination, were associated with significantly better sleep quality than standard treatment (oral conventional medications alone). These strategies included nerve block (SMD = 1.19, 95% CrI: 0.13 to 2.29), minimally invasive central nervous system neuromodulation (SMD = 2.43, 95% CrI: 1.27 to 3.78), minimally invasive peripheral nerve modulation + nerve block (SMD = 1.59, 95% CrI: 0.20 to 2.99), physically-ablative neuromodulation + nerve block (SMD = 2.12, 95% CrI: 0.28 to 4.04), minimally invasive peripheral nerve modulation + systemic pharmacological analgesia (SMD = 3.68, 95% CrI: 1.80 to 5.50), and minimally invasive central nervous system neuromodulation + topical and peripheral chemical interventions (SMD = 3.71, 95% CrI: 1.86 to 5.77) (Table 3).

**Table 3:** League table of sleep quality improvement outcomes.

**ST**													
**1.19 (0.13, 2.29)**	**NB**												
0.83 (−0.14, 1.74)	−0.36 (−1.38, 0.56)	**MI-PNM**											
−0.37 (−1.62, 0.81)	**−1.56 (−2.82, −0.41)**	**−1.2 (−2.01, −0.4)**	**Sham**										
**2.43 (1.27, 3.78)**	**1.24 (0.23, 2.4)**	**1.61 (0.75, 2.69)**	**2.81 (1.69, 4.17)**	**MI-CNS-NM**									
1.24 (−0.02, 2.53)	0.06 (−1.07, 1.16)	0.42 (−0.54, 1.45)	**1.62 (0.56, 2.76)**	−1.19 (−2.56, 0.02)	**TPCI**								
0.18 (−1.32, 1.68)	−1.01 (−2.88, 0.81)	−0.65 (−2.39, 1.15)	0.55 (−1.35, 2.51)	**−2.25 (−4.3, −0.39)**	−1.06 (−3.05, 0.89)	**PTEM**							
1.07 (−0.87, 2.96)	−0.11 (−2.06, 1.73)	0.25 (−1.44, 1.92)	1.44 (−0.01, 2.92)	−1.36 (−3.41, 0.43)	−0.17 (−2.05, 1.62)	0.89 (−1.57, 3.3)	**SPA**						
1.82 (−0.03, 3.72)	0.63 (−0.9, 2.16)	0.99 (−0.76, 2.86)	**2.19 (0.31, 4.19)**	−0.61 (−2.56, 1.19)	0.58 (−1.31, 2.48)	1.64 (−0.73, 4.08)	0.75 (−1.63, 3.24)	**T-PNES**					
−0.11 (−1.78, 1.51)	−1.29 (−2.98, 0.27)	−0.94 (−2.31, 0.42)	0.27 (−0.84, 1.36)	**−2.55 (−4.32, −1.01)**	−1.35 (−2.95, 0.16)	−0.29 (−2.54, 1.9)	−1.18 (−3.02, 0.65)	−1.93 (−4.23, 0.24)	**NI-CNS-NM**				
**1.59 (0.2, 2.99)**	0.4 (−0.81, 1.55)	0.76 (−0.39, 1.96)	**1.96 (0.59, 3.39)**	−0.84 (−2.35, 0.46)	0.34 (−1.09, 1.76)	1.41 (−0.64, 3.46)	0.51 (−1.48, 2.57)	−0.23 (−2.19, 1.67)	1.7 (−0.05, 3.51)	**MI-PNM + NB**			
**2.12 (0.28, 4.04)**	0.94 (−0.6, 2.47)	1.29 (−0.45, 3.16)	**2.49 (0.62, 4.51)**	−0.31 (−2.26, 1.49)	0.88 (−0.98, 2.78)	1.94 (−0.43, 4.36)	1.05 (−1.33, 3.54)	0.3 (−1.86, 2.46)	**2.23 (0.07, 4.52)**	0.53 (−1.36, 2.49)	**PAN + NB**		
**3.68 (1.8, 5.5)**	**2.49 (0.57, 4.29)**	**2.85 (1.26, 4.44)**	**4.05 (2.28, 5.83)**	1.24 (−0.73, 2.98)	**2.44 (0.52, 4.26)**	**3.5 (1.1, 5.85)**	**2.61 (0.31, 4.92)**	1.86 (−0.61, 4.21)	**3.79 (1.71, 5.89)**	**2.09 (0.1, 4.04)**	1.56 (−0.91, 3.9)	**MI-PNM + SPA**	
**3.71 (1.86, 5.77)**	**2.52 (0.76, 4.43)**	**2.88 (1.23, 4.79)**	**4.09 (2.28, 6.15)**	1.28 (−0.21, 2.77)	**2.47 (0.59, 4.52)**	**3.53 (1.16, 6.11)**	**2.64 (0.34, 5.19)**	1.89 (−0.43, 4.35)	**3.82 (1.73, 6.18)**	**2.12 (0.18, 4.27)**	1.59 (−0.72, 4.06)	0.04 (−2.22, 2.55)	**MI-CNS-NM + TPCI**

Values represent the standardized mean difference (SMD) and 95% credible interval (CrI) for the comparison of the row intervention versus the column intervention in terms of sleep quality improvement outcomes. A positive SMD indicates that the row intervention is superior to the column intervention, while a negative SMD indicates the opposite. Statistically significant comparisons (95% CrI excluding 0) are presented in boldface. Full intervention names corresponding to the abbreviations used can be found in Supplementary Table S5.

SUCRA ranking probabilities revealed the following order of effectiveness: minimally invasive central nervous system neuromodulation + topical and peripheral chemical interventions (94.57%), minimally invasive peripheral nerve modulation + systemic pharmacological analgesia (93.71%), and minimally invasive central nervous system neuromodulation (79.69%). Minimally invasive central nervous system neuromodulation + topical and peripheral chemical interventions demonstrated the greatest reduction in sleep quality scores (Figure 4B).

##### 3.3.2.3 AEs

Data on AEs were available from 24 studies. Most AEs were mild to moderate and transient, and severe events (e.g., pneumothorax) were rare. The NMA found no statistically significant differences in AE incidence between any intervention and standard oral treatment (as all 95% CrIs for the RR included 1). However, the wide range of RR estimates and their broad CrIs, however, suggest considerable uncertainty in these comparative safety profiles (Table 4).

**Table 4:** League table of adverse event incidence.

**ST**															
1.29 (0.24, 6.42)	**NB**														
0.82 (0.03, 20.59)	0.63 (0.03, 10.74)	**MI-PNM**													
1.1 (0.05, 20.17)	0.86 (0.06, 9.99)	1.35 (0.15, 12.16)	**Sham**												
0.49 (0.01, 26.77)	0.38 (0.01, 14.94)	0.61 (0.05, 6.47)	0.45 (0.02, 11.71)	**MI-CNS-NM**											
0.48 (0.02, 11.22)	0.37 (0.02, 5.76)	0.58 (0.03, 10.83)	0.43 (0.03, 6.93)	0.96 (0.02, 43.08)	**TPCI**										
1 (0.04, 24.85)	0.77 (0.02, 28.74)	1.23 (0.01, 133.82)	0.9 (0.01, 80.2)	2.03 (0.01, 396.54)	2.11 (0.02, 211.53)	**PTEM**									
0.12 (0, 13.93)	0.09 (0, 8.4)	0.15 (0, 11.93)	0.11 (0, 4.87)	0.24 (0, 36.89)	0.25 (0, 27.46)	0.12 (0, 37.58)	**SPA**								
0.56 (0.02, 14.46)	0.43 (0.02, 7.54)	0.68 (0.03, 18.5)	0.5 (0.02, 15.08)	1.12 (0.02, 68.28)	1.17 (0.03, 48.41)	0.55 (0.01, 53.7)	4.61 (0.03, 1199.88)	**T-PNES**							
1.21 (0.04, 34.15)	0.94 (0.02, 39.68)	1.5 (0.02, 176.09)	1.09 (0.01, 108.66)	2.47 (0.01, 510.81)	2.56 (0.03, 289.55)	1.23 (0.01, 123.81)	10.24 (0.03, 5847.25)	2.2 (0.02, 245.93)	**BioTx**						
0.75 (0.01, 84.92)	0.58 (0, 89.03)	0.92 (0, 319.22)	0.68 (0, 204.78)	1.53 (0, 839.25)	1.59 (0.01, 518.35)	0.76 (0, 224.42)	6.5 (0.01, 8025.7)	1.36 (0, 437.01)	0.61 (0, 198.55)	**MOT**					
32.05 (0.57, 3326.64)	24.52 (0.66, 1949.93)	39.81 (0.4, 7591.93)	29.34 (0.37, 4853.61)	67.11 (0.38, 20912.83)	68.6 (0.68, 12837.07)	33.33 (0.19, 8572.82)	284.04 (0.84, 223147.73)	58.71 (0.55, 10813.61)	27.38 (0.14, 7458.42)	44.23 (0.09, 30501.13)	**CSN**				
1.28 (0.03, 49.72)	0.99 (0.04, 26.64)	1.57 (0.02, 127.73)	1.15 (0.02, 78.69)	2.61 (0.02, 402.25)	2.72 (0.04, 215.84)	1.29 (0.01, 163.21)	10.78 (0.04, 4739.76)	2.32 (0.03, 185.82)	1.06 (0.01, 141.58)	1.71 (0, 640.44)	0.04 (0, 5.49)	**MI-PNM + NB**			
1.54 (0.04, 58.51)	1.19 (0.04, 32.11)	1.88 (0.03, 153.39)	1.38 (0.02, 93.62)	3.1 (0.02, 474.89)	3.22 (0.04, 258.88)	1.54 (0.01, 189.38)	12.85 (0.05, 5607.07)	2.79 (0.03, 226.09)	1.27 (0.01, 169.63)	2.05 (0.01, 787.31)	0.05 (0, 6.42)	1.21 (0.01, 127.77)	**PAN + NB**		
3.54 (0.02, 832.23)	2.72 (0.02, 511.17)	4.2 (0.09, 379.44)	3.16 (0.04, 475.64)	7.1 (0.08, 1152.06)	7.45 (0.06, 1506.78)	3.59 (0.01, 1896.67)	30.42 (0.09, 21759.82)	6.39 (0.04, 1512.36)	2.92 (0.01, 1686.26)	4.83 (0, 6184.35)	0.11 (0, 63.4)	2.81 (0.01, 1329.45)	2.32 (0.01, 1082.88)	**MI-PNM + SPA**	
0.89 (0.01, 105.23)	0.69 (0, 108.83)	1.1 (0, 390.01)	0.81 (0, 250)	1.83 (0, 1034.81)	1.87 (0.01, 656.9)	0.89 (0, 272.06)	7.6 (0.01, 9887.92)	1.61 (0, 541.6)	0.73 (0, 250.37)	1.19 (0, 965.16)	0.03 (0, 14.38)	0.7 (0, 293.35)	0.58 (0, 236.69)	0.25 (0, 294.6)	**MOT + NB**

Values represent the relative risk (RR) and 95% credible interval (CrI) for the comparison of the row intervention versus the column intervention in terms of adverse event incidence. An RR > 1 indicates that the row intervention is associated with a higher adverse event incidence than the column intervention, while an RR < 1 indicates the opposite. No comparisons showed statistical significance, as all 95% CrIs include 1. Full intervention names corresponding to the abbreviations used can be found in Supplementary Table S5.

SUCRA ranking probabilities indicated that minimally invasive central nervous system neuromodulation, after oral conventional medication, had the lowest probability of AEs (63.24%), followed by medical oxidant therapy plus nerve block (52.27%) and physical therapy and energy medicine (50.62%). In contrast, chemical selective neurolysis was ranked the lowest (7.97%), indicating the highest probability of AE incidence among all interventions (Figure 4C).

### 3.4 Bayesian meta-regression analysis

An assessment of transitivity (Table 1) revealed that, except for disease stage and lesion location, which exhibited the expected clinical variations, other factors, including age, sex, and baseline pain score, exhibited highly overlapping distributions across different comparison groups. These findings support the transitivity assumption underlying the NMA.

Bayesian meta-regression identified several significant effect modifiers. Regarding the pain relief outcome, the 95% CrIs for the regression coefficients of sample size, baseline pain score, disease stage, follow-up duration, age, and the proportion of female participants all excluded zero. This indicated their significant modifying effects on the treatment efficacy. No significant modifiers were detected for the sleep quality outcome. Regarding AEs, the 95% CrI for the regression coefficients of baseline pain score and sample size excluded zero. This suggested their significant influence on this outcome. After including these covariates, all models exhibited minimal residual heterogeneity (residual I^2^ < 10%). This finding implied a good model fit and that the majority of the heterogeneity had been accounted for. Crucially, despite these modifying effects, the relative ranking of intervention efficacy remained substantially unchanged, confirming the robustness of the primary analysis conclusions (Supplementary Table S2).

### 3.5 Publication bias

**Figure 5:**
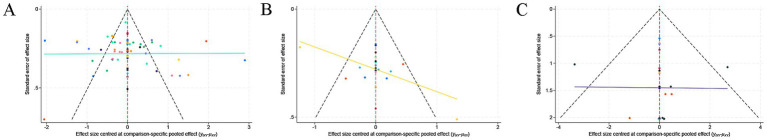
Comparison-adjusted funnel plot for publication bias assessment: **(A)** pain relief, **(B)** sleep quality improvement, and **(C)** adverse events.

Publication bias was evaluated using contour-enhanced funnel plots and Egger’s test. The funnel plots demonstrated a symmetrical distribution (Figure 5), suggesting no substantial publication bias. This visual assessment was corroborated by Egger’s test. The results of the test indicated no significant funnel plot asymmetry for any of the three outcomes: pain relief (*p* = 0.32), sleep quality (*p* = 0.57), and AEs (*p* = 0.41) (Supplementary Table S3).

### 3.6 CINeMA assessment

The CINeMA framework evaluation indicated an overall limited certainty of evidence for the three primary outcomes. Most comparisons for both pain relief and AEs were graded as “low” or “very low” certainty. The sleep quality outcome had the most favorable evidence profile, with 12 comparisons rated as “high” certainty. Nevertheless, over two-thirds (68.1%) of its comparisons had “low” or “very low” certainty. The primary reasons for downgrading the evidence were imprecision (wide CrIs) and within-study bias. The complete assessment results are available in Supplementary Table S4.

## 4 Discussion

This NMA revealed that chemical selective neurolysis had the greatest potential to alleviate ZAP. Two combination therapies, minimally invasive central nervous system neuromodulation + topical and peripheral chemical interventions, and minimally invasive peripheral nerve modulation + systemic pharmacological analgesia, demonstrated significant benefits for both pain relief and sleep improvement. A safety analysis suggested that minimally invasive central nervous system neuromodulation was associated with the lowest probability of AEs. Furthermore, most interventions exhibited a favorable safety profile. AEs were typically mild and transient.

Chemical selective neurolysis (doxorubicin-mediated DRG destruction) ranked first in the SUCRA for pain relief. However, it is important to note that SUCRA values reflect probabilistic ranking among interventions rather than definitive clinical superiority. Previous studies have confirmed the efficacy of doxorubicin-mediated selective DRG destruction in alleviating PHN and postherpetic trigeminal neuralgia. This suggests a potential approach to neuropathic pain management (75, 76). Doxorubicin is a broad-spectrum anthracycline antibiotic with cytotoxic properties that diffuses through the vascular pores of the DRG following paravertebral injection. It then undergoes retrograde axonal transport and accumulates selectively in the nuclei and nucleoli of sensory neurons (77). This accumulation induces chromatin condensation, nuclear membrane damage, and structural alterations in the nuclear pore complex, thereby inhibiting protein synthesis. The ultimate consequence is the necrosis of sensory neurons and Wallerian degeneration of associated nerve fibers, which effectively blocks pain signal transmission (78). Notably, this agent minimally impacts motor nerve roots, enabling the selective treatment of neuropathic pain in targeted areas (79). Regarding safety, doxorubicin therapy is associated with a relatively high incidence of adverse reactions, though severe complications are uncommon. The most frequent adverse effects are local numbness and hypoesthesia, which are primarily caused by ganglion cell necrosis, mild lymphocyte infiltration, and subsequent cellular loss (79). These symptoms are generally well-tolerated and seldom require specific intervention. Despite these findings, the clinical adoption of this therapy is limited by a lack of evidence and ethical considerations. First, the current evidence base is exceedingly limited: only a single-center randomized controlled trial (RCT) has evaluated the efficacy and safety of doxorubicin-mediated DRG destruction for PHN, and its follow-up duration was short. Consequently, the long-term benefits and potential risks must be validated through larger, multicenter studies. Second, as an irreversible, destructive intervention, this procedure causes permanent sensory loss and other irreversible neurological damage, raising significant ethical concerns (80). Currently, clinical practice guidelines for ZAP do not widely recommend such destructive procedures. The absence of robust, long-term safety data further restricts its clinical application scenarios (81).

Beyond pain, ZAP often presents with sleep disturbances, anxiety, depression, and impaired concentration. These symptoms substantially impair health-related quality of life in daily activities, work, and social functioning (82). Prior studies have suggested that minimally invasive central nervous system neuromodulation is more effective than minimally invasive peripheral nerve modulation in reducing pain, improving sleep, and reducing AEs. This supports the use of single-intervention strategies (83, 84). Nevertheless, the comparative effects and safety of different combination therapies have not yet been fully quantified using Bayesian NMA. The present results indicate that minimally invasive central nervous system neuromodulation combined with topical and peripheral chemical interventions, as well as minimally invasive peripheral nerve modulation combined with systemic pharmacological analgesia, yield favorable outcomes consistent with synergistic mechanisms. Neuromodulation inhibits central and peripheral pain signaling. For example, minimally invasive central nervous system neuromodulation activates A-*β* fibers, which suppresses C-fiber transmission. Conversely, minimally invasive peripheral nerve modulation targets nociceptive fibers (7, 85). Chemical and pharmacological interventions block sodium channels or reduce inflammation (86). This synergy may explain why combination therapies outperform monotherapies, as observed in sleep quality outcomes (87).

In this NMA, minimally invasive central nervous system neuromodulation, medical oxidant therapy plus nerve block, and physical therapy and energy medicine demonstrated favorable safety profiles, with a low incidence of AEs. The AEs reported in the included studies were predominantly mild to moderate and transient. Common reactions included local symptoms (e.g., injection-site pain, numbness, and mild hematoma) and systemic effects (e.g., nausea, dizziness, and somnolence). Although serious complications were uncommon, specific events such as pneumothorax, catheter dislodgement, and transient visual impairment were occasionally observed. Therefore, clinicians should still perform individualized assessments when selecting treatment strategies. According to relevant medical guidelines and clinical experience, this type of interventional therapy is not recommended for patients with puncture-site infections or skin ulcers, severe coagulation disorders, poor cooperation or inability to tolerate the procedure, significant anatomical abnormalities, or known drug hypersensitivity.

This review strictly adhered to the PRISMA and PRISMA-NMA reporting standards. Four major English databases, including Embase and PubMed, were searched thoroughly, and only high-quality RCTs were included. High-quality RCTs have a robust design and implementation, which effectively minimizes bias and ensures evidence homogeneity and strength of inference. Furthermore, this NMA provides a highly comprehensive assessment of ZAP management, covering a wider array of interventions than previous reports. This NMA uniquely synthesizes evidence from various categories, including physical, interventional, and alternative approaches, while examining the efficacy of combination regimens. Thus, it overcomes the limitations of previous studies that focused on isolated treatments.

Some limitations should be acknowledged. First, although key effect modifiers for different outcomes were identified via Bayesian meta-regression and core treatment rankings were maintained, considerable heterogeneity was observed among the included studies. Local inconsistency was detected within certain closed loops, which may affect the reliability of the associated effect size estimates. Second, the CINeMA evaluation revealed that the certainty of the evidence for most comparisons was low or very low, primarily due to imprecision (i.e., wide CrIs) and risks of bias within the original studies (e.g., lack of blinding). Third, the evidence base for key interventions remains limited. For example, data on the efficacy and safety of chemical selective neurolysis were derived from only a single, small-sample RCT. Additionally, the insufficient reporting of patient-important outcomes, such as anxiety, depression, and quality of life, constrained a comprehensive assessment of therapeutic benefits. Fourth, restricting the analysis to English-language publications may introduce language bias and limit the generalizability of the findings. Finally, the Bayesian meta-regression was conducted using aggregate study-level data, which limits the ability to make causal inferences.

### 4.1 Future prospects

Future research should include more high-quality, multicenter RCTs with long-term follow-up to verify the present findings, while also reporting multidimensional outcomes beyond pain intensity, including sleep quality, psychosocial function, and quality of life. From a methodological perspective, more emphasis should be placed on blinding procedures, such as using blinded outcome assessors and conducting sham-controlled interventions, to mitigate performance and detection biases. From a mechanistic research perspective, future investigations should extend beyond animal models and integrate multi-omics approaches to explore human biochemical markers and network mechanisms. Elucidating the foundational actions of different therapies will concurrently provide deeper evidence of their clinical value. Furthermore, current interventional research predominantly focuses on monotherapies and lacks in-depth exploration of combined and comprehensive treatment strategies. There is an urgent need to conduct RCTs specifically designed for combination regimens. These trials are essential for clarifying the relative efficacy of these regimens, optimizing treatment pathways, and identifying the patient subgroups that are most likely to benefit.

## 5 Conclusion

Based on the present analysis, minimally invasive central nervous system neuromodulation combined with topical and peripheral chemical interventions, as well as minimally invasive peripheral nerve modulation combined with systemic pharmacological analgesia, are relatively safe and effective options for treating ZAP. These options demonstrate favorable performance in terms of both pain relief and sleep quality improvement. For refractory and intractable ZAP, chemical selective neurolysis (doxorubicin-mediated dorsal root ganglion destruction) may be considered as a potential alternative, though its use requires careful consideration of the limited supporting evidence and lack of long-term safety data, as well as the ethical implications. Due to the low to very low certainty of the overall evidence, study heterogeneity, and short follow-up durations in the included trials, these findings should be interpreted with caution. Clinical practice should prioritize individualized treatment decisions based on patient characteristics and disease severity. Further large-scale, multicenter RCTs with long-term follow-up are warranted to validate the efficacy and safety of these interventions.

## Data Availability

The original contributions presented in the study are included in the article/Supplementary material, further inquiries can be directed to the corresponding author.
