# Supplementary material for: Comparison of efficacy and safety of non-oral therapeutic interventions for zoster-associated pain: a systematic review and network meta-analysis
Source: Front Neurol. 2026 Jan 27;17:1711536. doi: 10.3389/fneur.2026.1711536 (PMC12886049; doi:10.3389/fneur.2026.1711536)
Supplement: Supplementary file 1 [file Data_Sheet_1.zip › Supplementary_Material_Complete/Data Sheet 1.pdf]

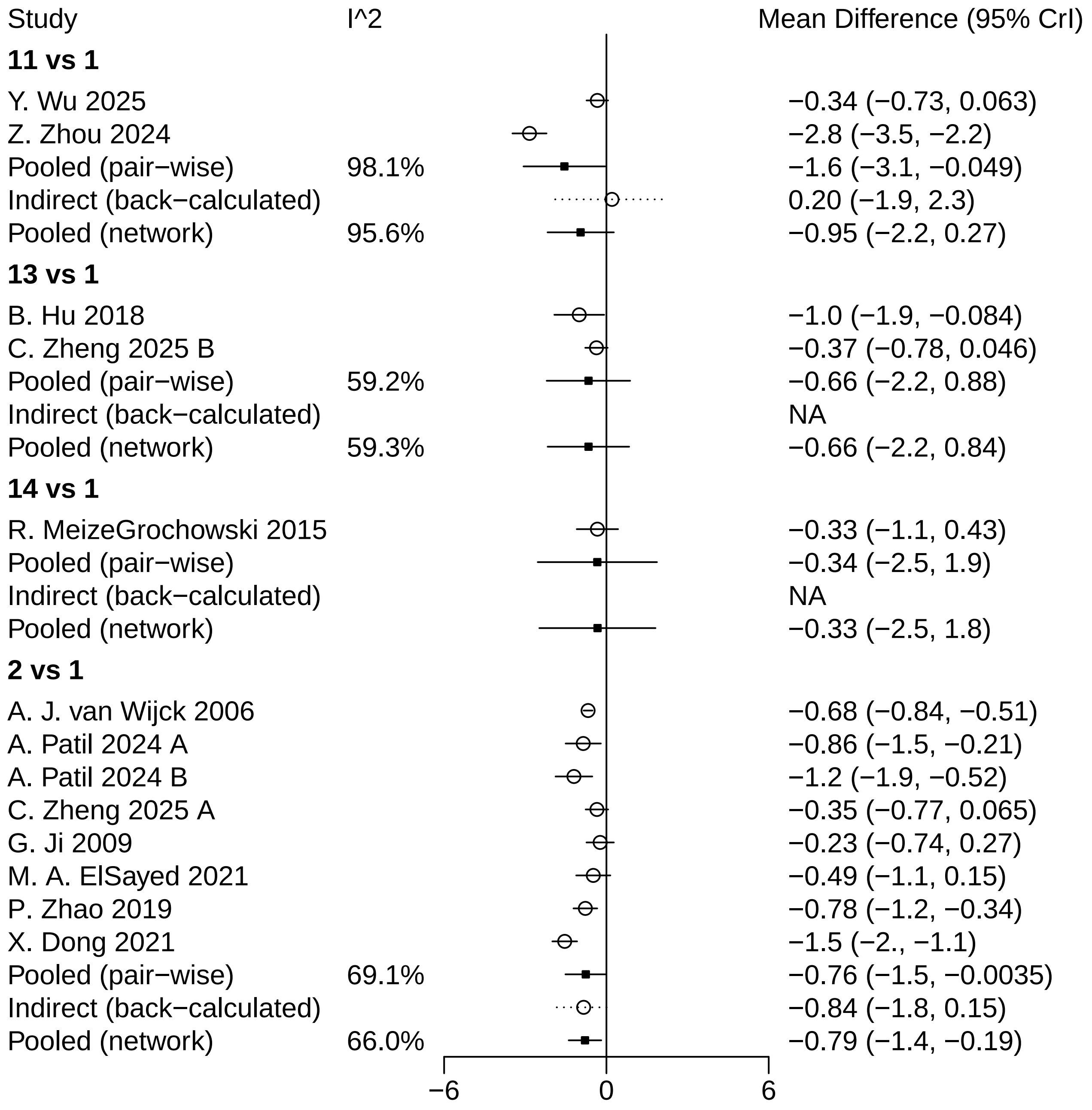
Study
I^2
Mean Difference (95% CrI)
11 vs 1
Y. Wu 2025
−0.34 (−0.73, 0.063)
Z. Zhou 2024
−2.8 (−3.5, −2.2)
Pooled (pair−wise)
98.1%
−1.6 (−3.1, −0.049)
Indirect (back−calculated)
0.20 (−1.9, 2.3)
Pooled (network)
95.6%
−0.95 (−2.2, 0.27)
13 vs 1
B. Hu 2018
−1.0 (−1.9, −0.084)
C. Zheng 2025 B
−0.37 (−0.78, 0.046)
Pooled (pair−wise)
59.2%
−0.66 (−2.2, 0.88)
Indirect (back−calculated)
NA
Pooled (network)
59.3%
−0.66 (−2.2, 0.84)
14 vs 1
R. MeizeGrochowski 2015
−0.33 (−1.1, 0.43)
Pooled (pair−wise)
−0.34 (−2.5, 1.9)
Indirect (back−calculated)
NA
Pooled (network)
−0.33 (−2.5, 1.8)
2 vs 1
A. J. van Wijck 2006
−0.68 (−0.84, −0.51)
A. Patil 2024 A
−0.86 (−1.5, −0.21)
A. Patil 2024 B
−1.2 (−1.9, −0.52)
C. Zheng 2025 A
−0.35 (−0.77, 0.065)
G. Ji 2009
−0.23 (−0.74, 0.27)
M. A. ElSayed 2021
−0.49 (−1.1, 0.15)
P. Zhao 2019
−0.78 (−1.2, −0.34)
X. Dong 2021
−1.5 (−2., −1.1)
Pooled (pair−wise)
69.1%
−0.76 (−1.5, −0.0035)
Indirect (back−calculated)
−0.84 (−1.8, 0.15)
Pooled (network)
66.0%
−0.79 (−1.4, −0.19)
−6
0
6

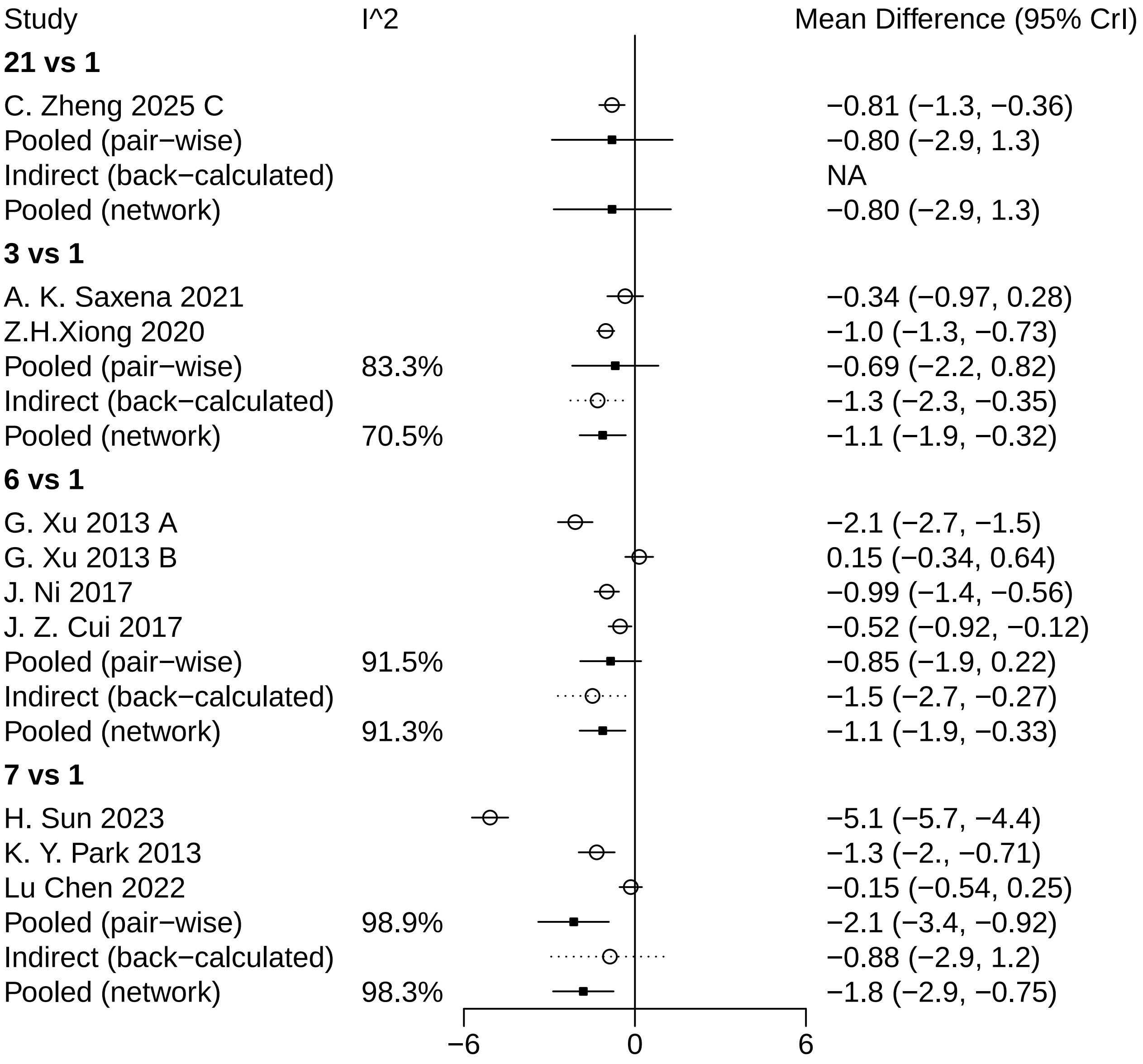
Study
I^2
Mean Difference (95% CrI)
21 vs 1
C. Zheng 2025 C
−0.81 (−1.3, −0.36)
Pooled (pair−wise)
−0.80 (−2.9, 1.3)
Indirect (back−calculated)
NA
Pooled (network)
−0.80 (−2.9, 1.3)
3 vs 1
A. K. Saxena 2021
−0.34 (−0.97, 0.28)
Z.H.Xiong 2020
−1.0 (−1.3, −0.73)
Pooled (pair−wise)
83.3%
−0.69 (−2.2, 0.82)
Indirect (back−calculated)
−1.3 (−2.3, −0.35)
Pooled (network)
70.5%
−1.1 (−1.9, −0.32)
6 vs 1
G. Xu 2013 A
−2.1 (−2.7, −1.5)
G. Xu 2013 B
0.15 (−0.34, 0.64)
J. Ni 2017
−0.99 (−1.4, −0.56)
J. Z. Cui 2017
−0.52 (−0.92, −0.12)
Pooled (pair−wise)
91.5%
−0.85 (−1.9, 0.22)
Indirect (back−calculated)
−1.5 (−2.7, −0.27)
Pooled (network)
91.3%
−1.1 (−1.9, −0.33)
7 vs 1
H. Sun 2023
−5.1 (−5.7, −4.4)
K. Y. Park 2013
−1.3 (−2., −0.71)
Lu Chen 2022
−0.15 (−0.54, 0.25)
Pooled (pair−wise)
98.9%
−2.1 (−3.4, −0.92)
Indirect (back−calculated)
−0.88 (−2.9, 1.2)
Pooled (network)
98.3%
−1.8 (−2.9, −0.75)
−6
0
6

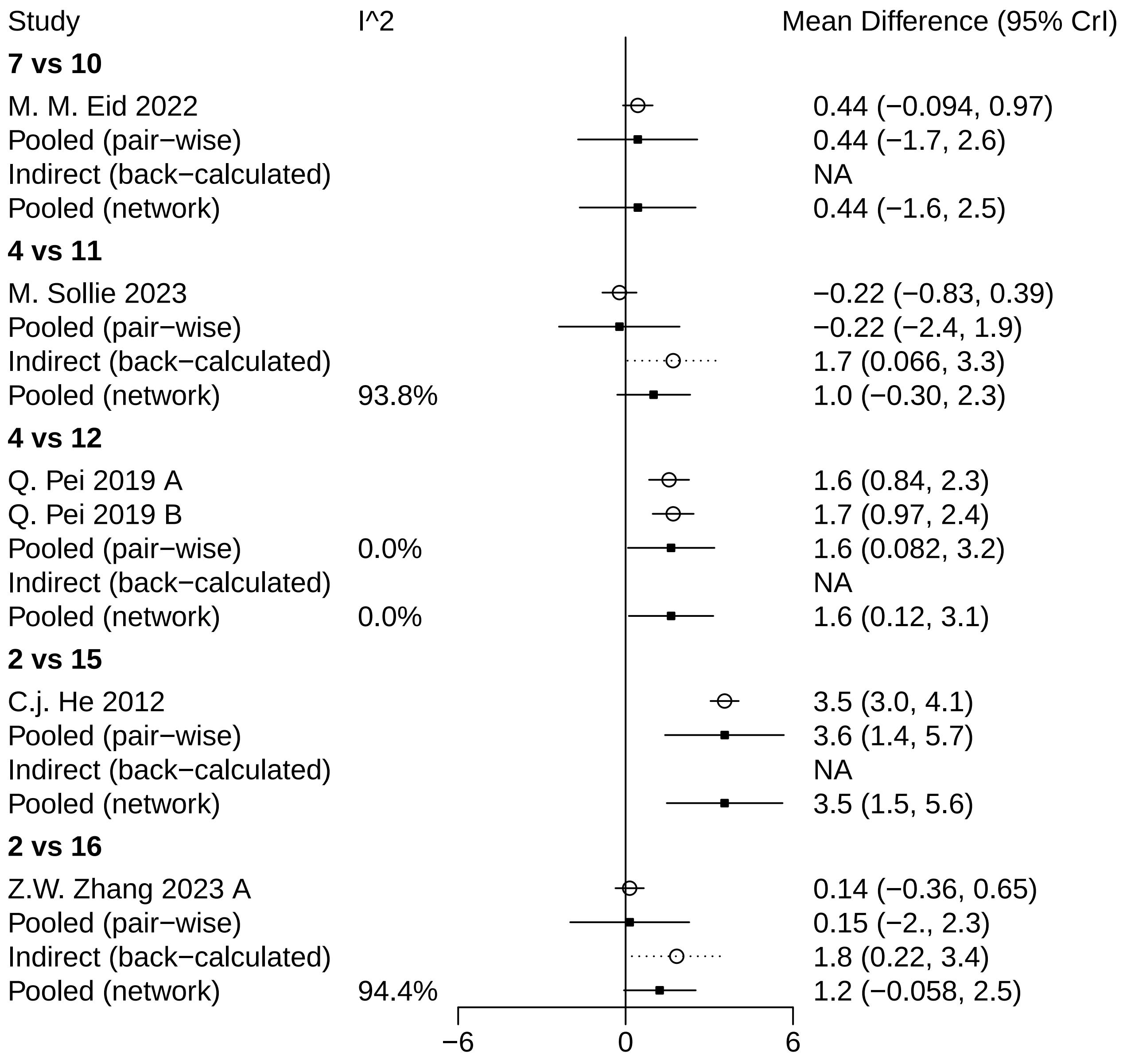
Study
I^2
Mean Difference (95% CrI)
7 vs 10
M. M. Eid 2022
0.44 (−0.094, 0.97)
Pooled (pair−wise)
0.44 (−1.7, 2.6)
Indirect (back−calculated)
NA
Pooled (network)
0.44 (−1.6, 2.5)
4 vs 11
M. Sollie 2023
−0.22 (−0.83, 0.39)
Pooled (pair−wise)
−0.22 (−2.4, 1.9)
Indirect (back−calculated)
1.7 (0.066, 3.3)
Pooled (network)
93.8%
1.0 (−0.30, 2.3)
4 vs 12
Q. Pei 2019 A
1.6 (0.84, 2.3)
Q. Pei 2019 B
1.7 (0.97, 2.4)
Pooled (pair−wise)
0.0%
1.6 (0.082, 3.2)
Indirect (back−calculated)
NA
Pooled (network)
0.0%
1.6 (0.12, 3.1)
2 vs 15
C.j. He 2012
3.5 (3.0, 4.1)
Pooled (pair−wise)
3.6 (1.4, 5.7)
Indirect (back−calculated)
NA
Pooled (network)
3.5 (1.5, 5.6)
2 vs 16
Z.W. Zhang 2023 A
0.14 (−0.36, 0.65)
Pooled (pair−wise)
0.15 (−2., 2.3)
Indirect (back−calculated)
1.8 (0.22, 3.4)
Pooled (network)
94.4%
1.2 (−0.058, 2.5)
−6
0
6

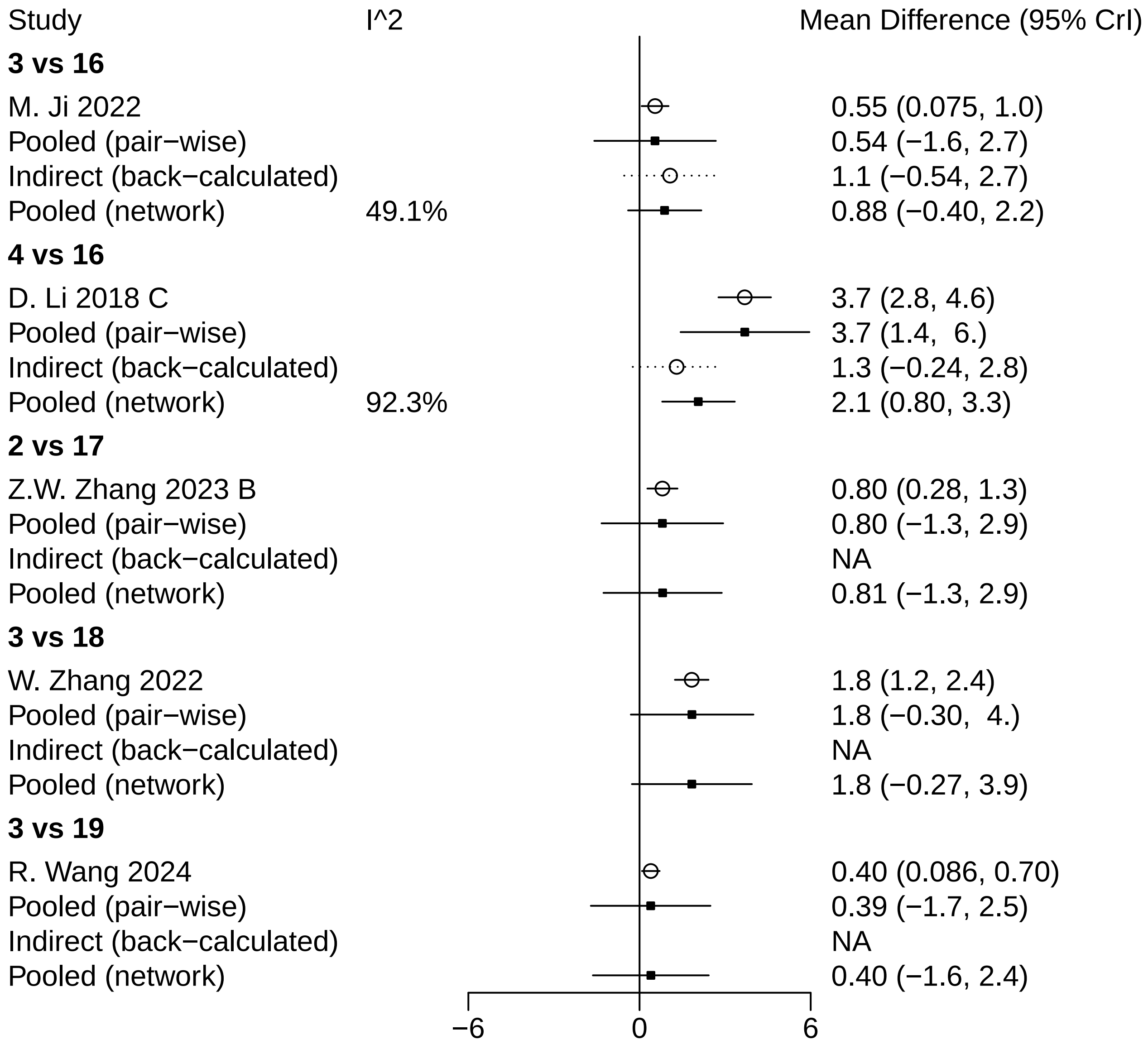

Study
I^2
Mean Difference (95% CrI)
3 vs 16
M. Ji 2022
0.55 (0.075, 1.0)
Pooled (pair−wise)
0.54 (−1.6, 2.7)
Indirect (back−calculated)
1.1 (−0.54, 2.7)
Pooled (network)
49.1%
0.88 (−0.40, 2.2)
4 vs 16
D. Li 2018 C
3.7 (2.8, 4.6)
Pooled (pair−wise)
3.7 (1.4, 6.)
Indirect (back−calculated)
1.3 (−0.24, 2.8)
Pooled (network)
92.3%
2.1 (0.80, 3.3)
2 vs 17
Z.W. Zhang 2023 B
0.80 (0.28, 1.3)
Pooled (pair−wise)
0.80 (−1.3, 2.9)
Indirect (back−calculated)
NA
Pooled (network)
0.81 (−1.3, 2.9)
3 vs 18
W. Zhang 2022
1.8 (1.2, 2.4)
Pooled (pair−wise)
1.8 (−0.30, 4.)
Indirect (back−calculated)
NA
Pooled (network)
1.8 (−0.27, 3.9)
3 vs 19
R. Wang 2024
0.40 (0.086, 0.70)
Pooled (pair−wise)
0.39 (−1.7, 2.5)
Indirect (back−calculated)
NA
Pooled (network)
0.40 (−1.6, 2.4)
−6
0
6

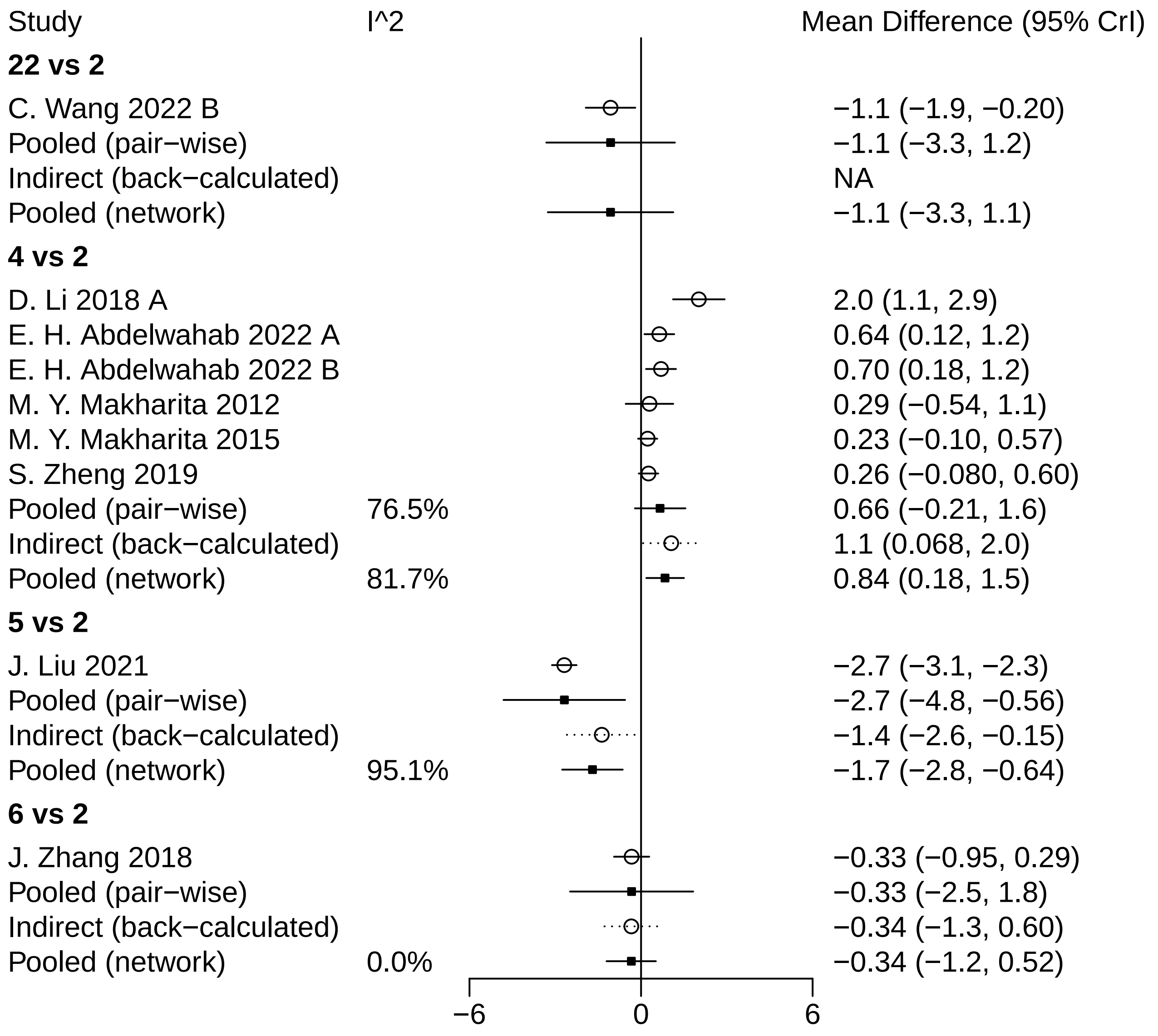
Study
I^2
Mean Difference (95% CrI)
22 vs 2
C. Wang 2022 B
−1.1 (−1.9, −0.20)
Pooled (pair−wise)
−1.1 (−3.3, 1.2)
Indirect (back−calculated)
NA
Pooled (network)
−1.1 (−3.3, 1.1)
4 vs 2
D. Li 2018 A
2.0 (1.1, 2.9)
E. H. Abdelwahab 2022 A
0.64 (0.12, 1.2)
E. H. Abdelwahab 2022 B
0.70 (0.18, 1.2)
M. Y. Makharita 2012
0.29 (−0.54, 1.1)
M. Y. Makharita 2015
0.23 (−0.10, 0.57)
S. Zheng 2019
0.26 (−0.080, 0.60)
Pooled (pair−wise)
76.5%
0.66 (−0.21, 1.6)
Indirect (back−calculated)
1.1 (0.068, 2.0)
Pooled (network)
81.7%
0.84 (0.18, 1.5)
5 vs 2
J. Liu 2021
−2.7 (−3.1, −2.3)
Pooled (pair−wise)
−2.7 (−4.8, −0.56)
Indirect (back−calculated)
−1.4 (−2.6, −0.15)
Pooled (network)
95.1%
−1.7 (−2.8, −0.64)
6 vs 2
J. Zhang 2018
−0.33 (−0.95, 0.29)
Pooled (pair−wise)
−0.33 (−2.5, 1.8)
Indirect (back−calculated)
−0.34 (−1.3, 0.60)
Pooled (network)
0.0%
−0.34 (−1.2, 0.52)
−6
0
6

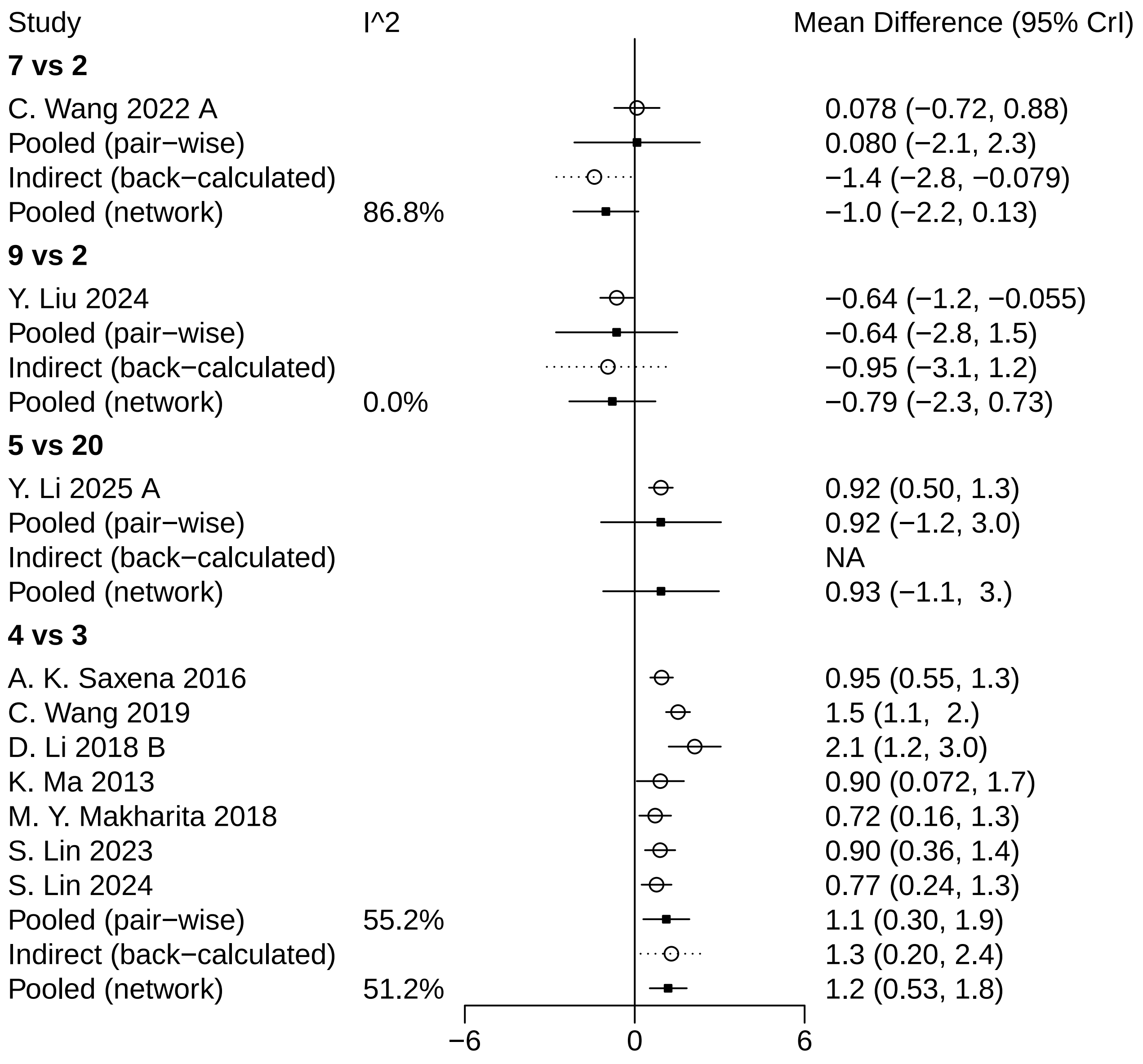
Study
I^2
Mean Difference (95% CrI)
7 vs 2
C. Wang 2022 A
0.078 (−0.72, 0.88)
Pooled (pair−wise)
0.080 (−2.1, 2.3)
Indirect (back−calculated)
−1.4 (−2.8, −0.079)
Pooled (network)
86.8%
−1.0 (−2.2, 0.13)
9 vs 2
Y. Liu 2024
−0.64 (−1.2, −0.055)
Pooled (pair−wise)
−0.64 (−2.8, 1.5)
Indirect (back−calculated)
−0.95 (−3.1, 1.2)
Pooled (network)
0.0%
−0.79 (−2.3, 0.73)
5 vs 20
Y. Li 2025 A
0.92 (0.50, 1.3)
Pooled (pair−wise)
0.92 (−1.2, 3.0)
Indirect (back−calculated)
NA
Pooled (network)
0.93 (−1.1, 3.)
4 vs 3
A. K. Saxena 2016
0.95 (0.55, 1.3)
C. Wang 2019
1.5 (1.1, 2.)
D. Li 2018 B
2.1 (1.2, 3.0)
K. Ma 2013
0.90 (0.072, 1.7)
M. Y. Makharita 2018
0.72 (0.16, 1.3)
S. Lin 2023
0.90 (0.36, 1.4)
S. Lin 2024
0.77 (0.24, 1.3)
Pooled (pair−wise)
55.2%
1.1 (0.30, 1.9)
Indirect (back−calculated)
1.3 (0.20, 2.4)
Pooled (network)
51.2%
1.2 (0.53, 1.8)
−6
0
6

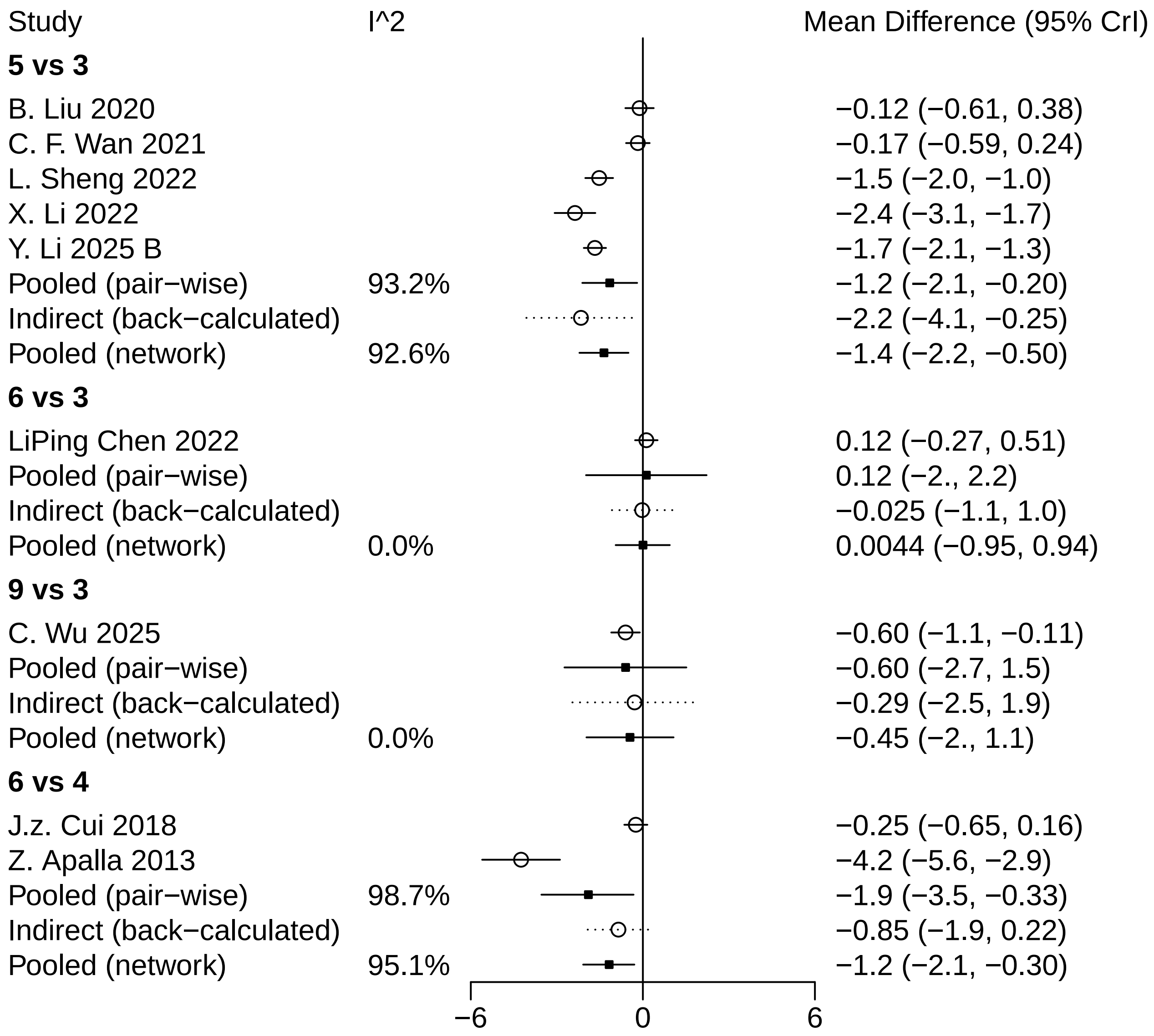
Study
I^2
Mean Difference (95% CrI)
5 vs 3
B. Liu 2020 −0.12 (−0.61, 0.38)
C. F. Wan 2021 −0.17 (−0.59, 0.24)
L. Sheng 2022 −1.5 (−2.0, −1.0)
X. Li 2022 −2.4 (−3.1, −1.7)
Y. Li 2025 B −1.7 (−2.1, −1.3)
Pooled (pair−wise) 93.2% −1.2 (−2.1, −0.20)
Indirect (back−calculated) −2.2 (−4.1, −0.25)
Pooled (network) 92.6% −1.4 (−2.2, −0.50)
6 vs 3
LiPing Chen 2022 0.12 (−0.27, 0.51)
Pooled (pair−wise) 0.12 (−2., 2.2)
Indirect (back−calculated) −0.025 (−1.1, 1.0)
Pooled (network) 0.0% 0.0044 (−0.95, 0.94)
9 vs 3
C. Wu 2025 −0.60 (−1.1, −0.11)
Pooled (pair−wise) −0.60 (−2.7, 1.5)
Indirect (back−calculated) −0.29 (−2.5, 1.9)
Pooled (network) 0.0% −0.45 (−2., 1.1)
6 vs 4
J.z. Cui 2018 −0.25 (−0.65, 0.16)
Z. Apalla 2013 −4.2 (−5.6, −2.9)
Pooled (pair−wise) 98.7% −1.9 (−3.5, −0.33)
Indirect (back−calculated) −0.85 (−1.9, 0.22)
Pooled (network) 95.1% −1.2 (−2.1, −0.30)
−6
0
6

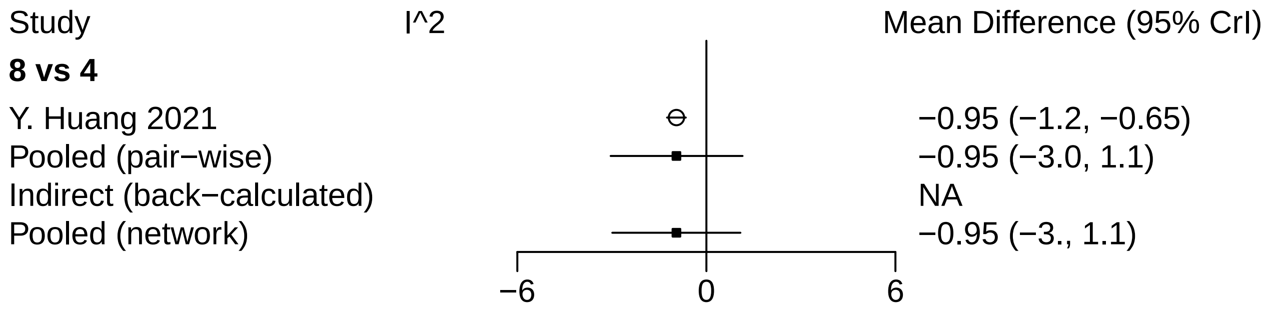

Supplementary Figure 1 Forest plots with heterogeneity assessment for the pain relief outcome.
Note: This forest plot presents the evidence synthesis results for each direct comparison regarding the pain relief outcome, based on a Bayesian random-effects model. It displays the direct pairwise meta-analysis results, indirect effect estimates, and the comprehensive network meta-analysis estimates for each comparison. All effect estimates are expressed as mean difference (MD) with 95% credible intervals (95% CrI). The I^2 statistic was used to assess local heterogeneity, with higher values indicating greater heterogeneity. The correspondence between intervention codes/abbreviations and their full names is provided in Table S5.
