# Supplementary material for: Comparison of efficacy and safety of non-oral therapeutic interventions for zoster-associated pain: a systematic review and network meta-analysis
Source: Front Neurol. 2026 Jan 27;17:1711536. doi: 10.3389/fneur.2026.1711536 (PMC12886049; doi:10.3389/fneur.2026.1711536)
Supplement: Supplementary file 1 [file Data_Sheet_1.zip › Supplementary_Material_Complete/Data Sheet 10.pdf]

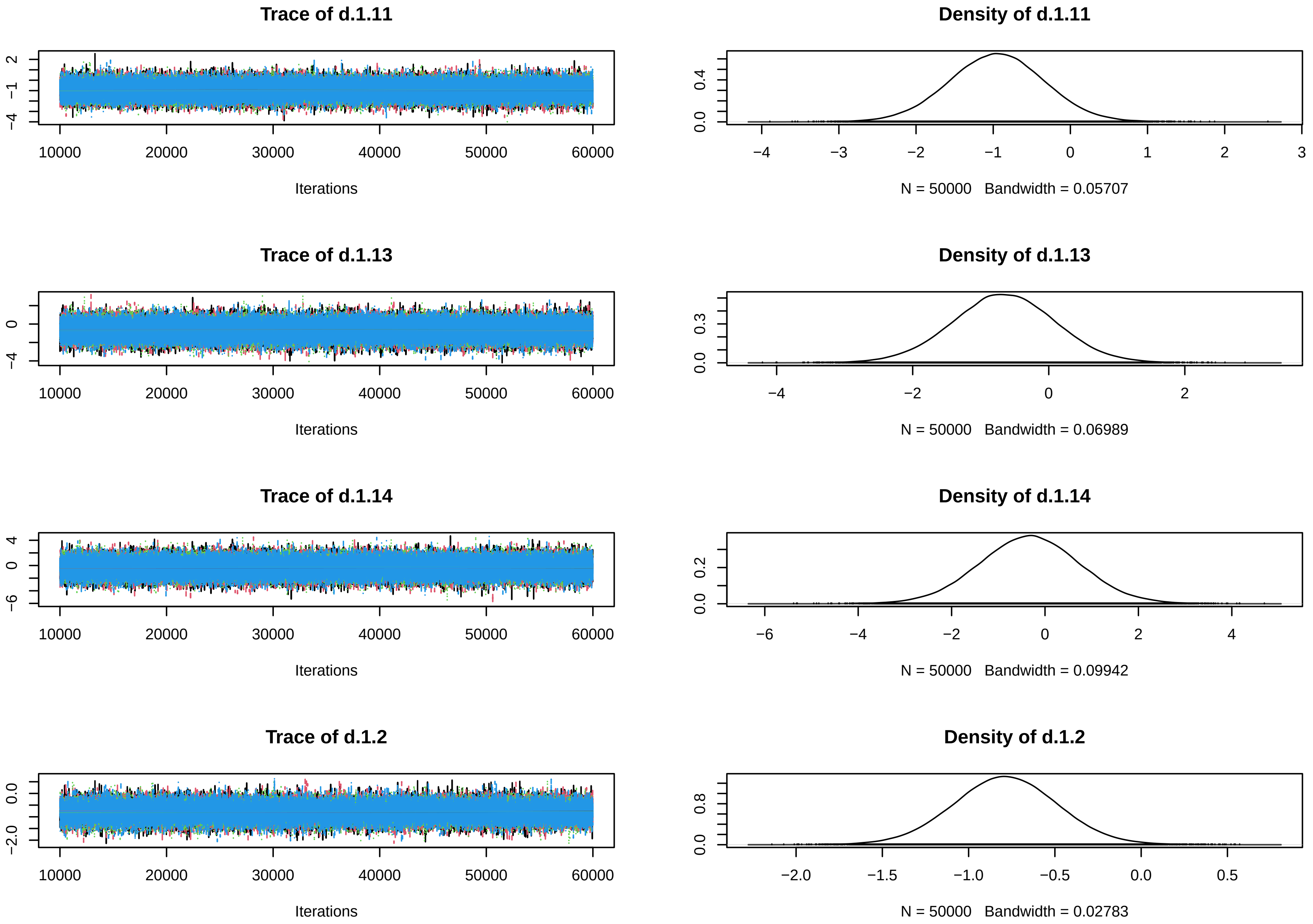
Trace of d.1.11
Iterations
Density of d.1.11
N = 50000 Bandwidth = 0.05707
Trace of d.1.13
Iterations
Density of d.1.13
N = 50000 Bandwidth = 0.06989
Trace of d.1.14
Iterations
Density of d.1.14
N = 50000 Bandwidth = 0.09942
Trace of d.1.2
Iterations
Density of d.1.2
N = 50000 Bandwidth = 0.02783

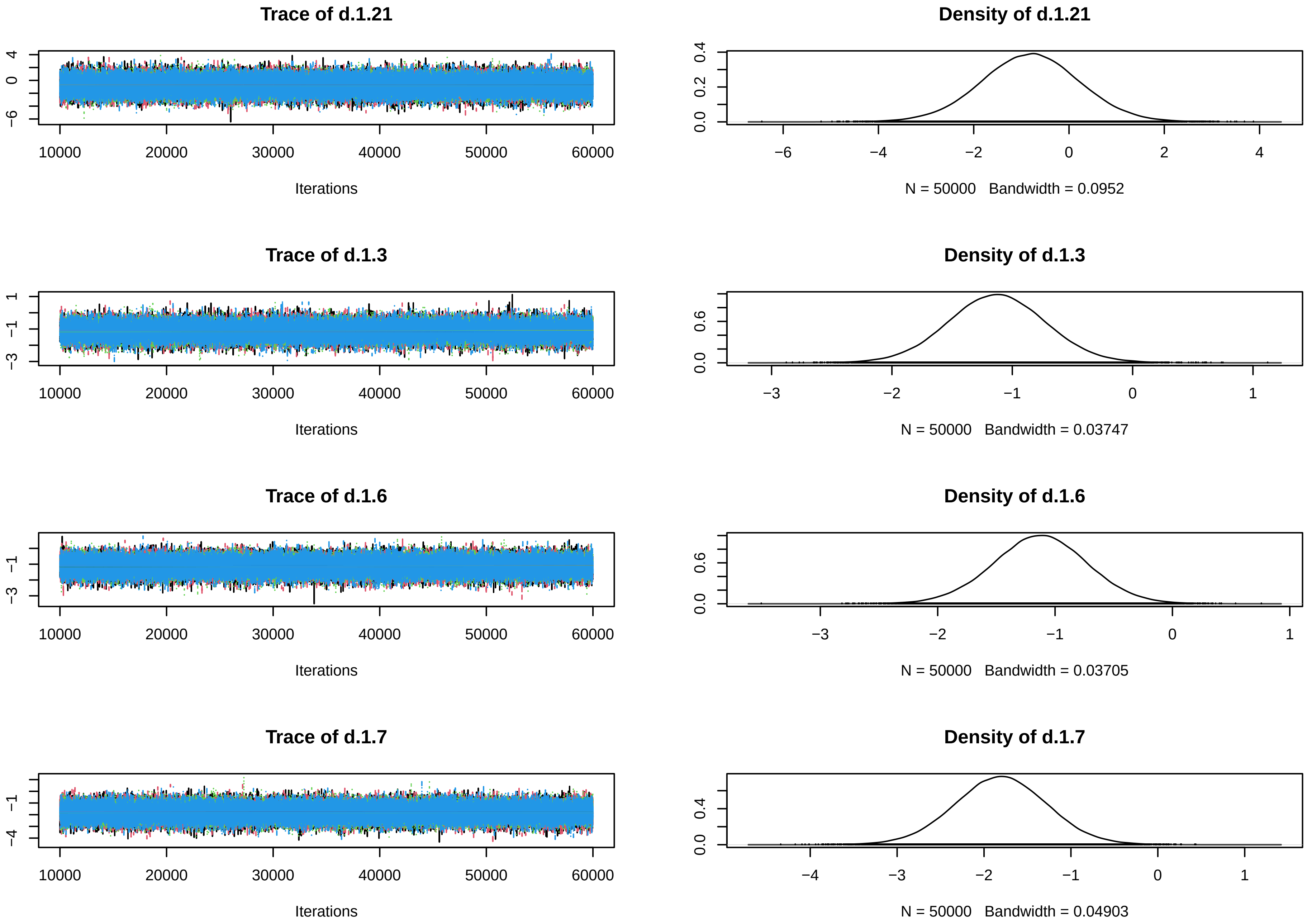
Trace of d.1.21
Iterations
Density of d.1.21
N = 50000 Bandwidth = 0.0952
Trace of d.1.3
Iterations
Density of d.1.3
N = 50000 Bandwidth = 0.03747
Trace of d.1.6
Iterations
Density of d.1.6
N = 50000 Bandwidth = 0.03705
Trace of d.1.7
Iterations
Density of d.1.7
N = 50000 Bandwidth = 0.04903

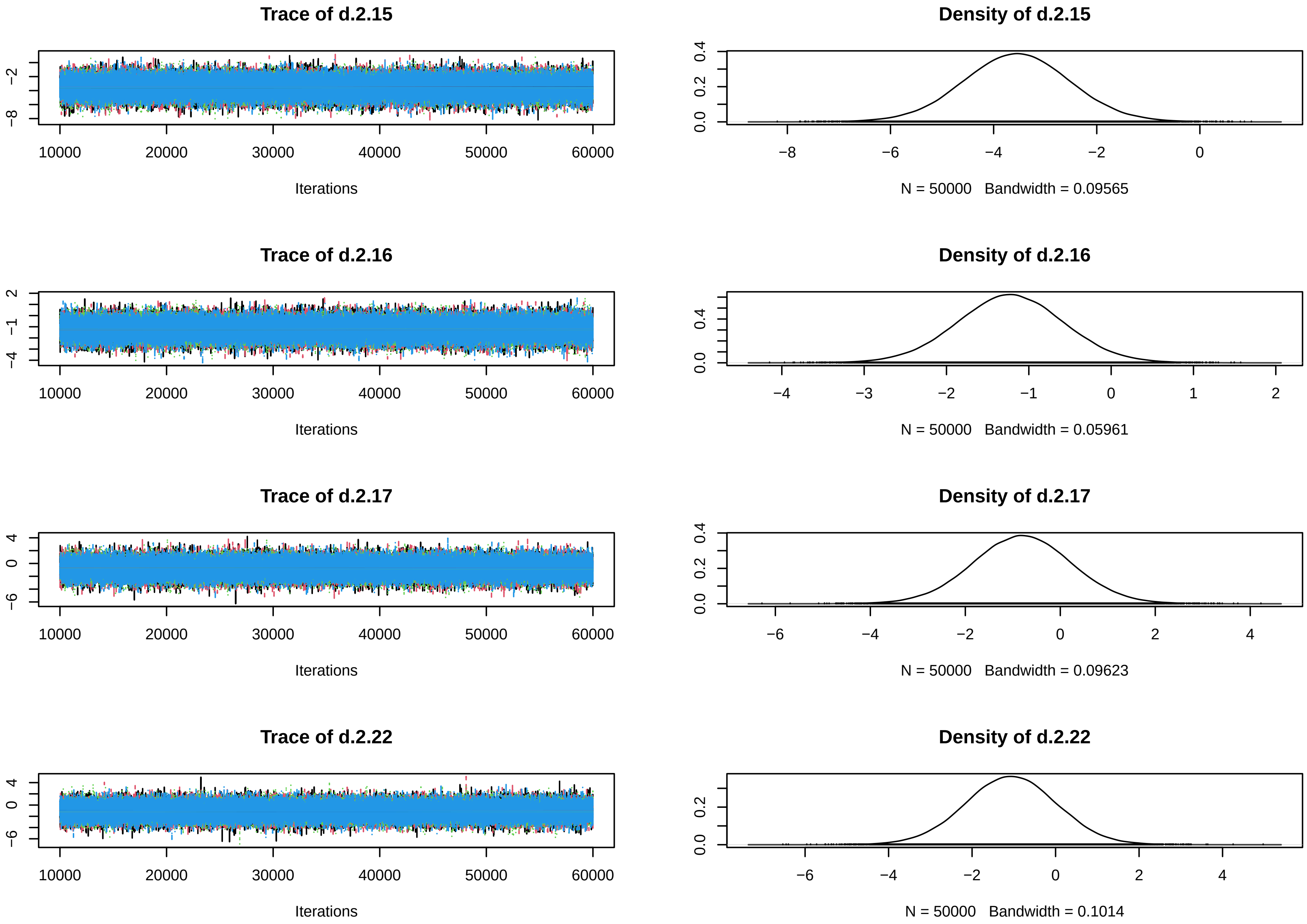
Trace of d.2.15
Iterations
Density of d.2.15
N = 50000   Bandwidth = 0.09565
Trace of d.2.16
Iterations
Density of d.2.16
N = 50000   Bandwidth = 0.05961
Trace of d.2.17
Iterations
Density of d.2.17
N = 50000   Bandwidth = 0.09623
Trace of d.2.22
Iterations
Density of d.2.22
N = 50000   Bandwidth = 0.1014

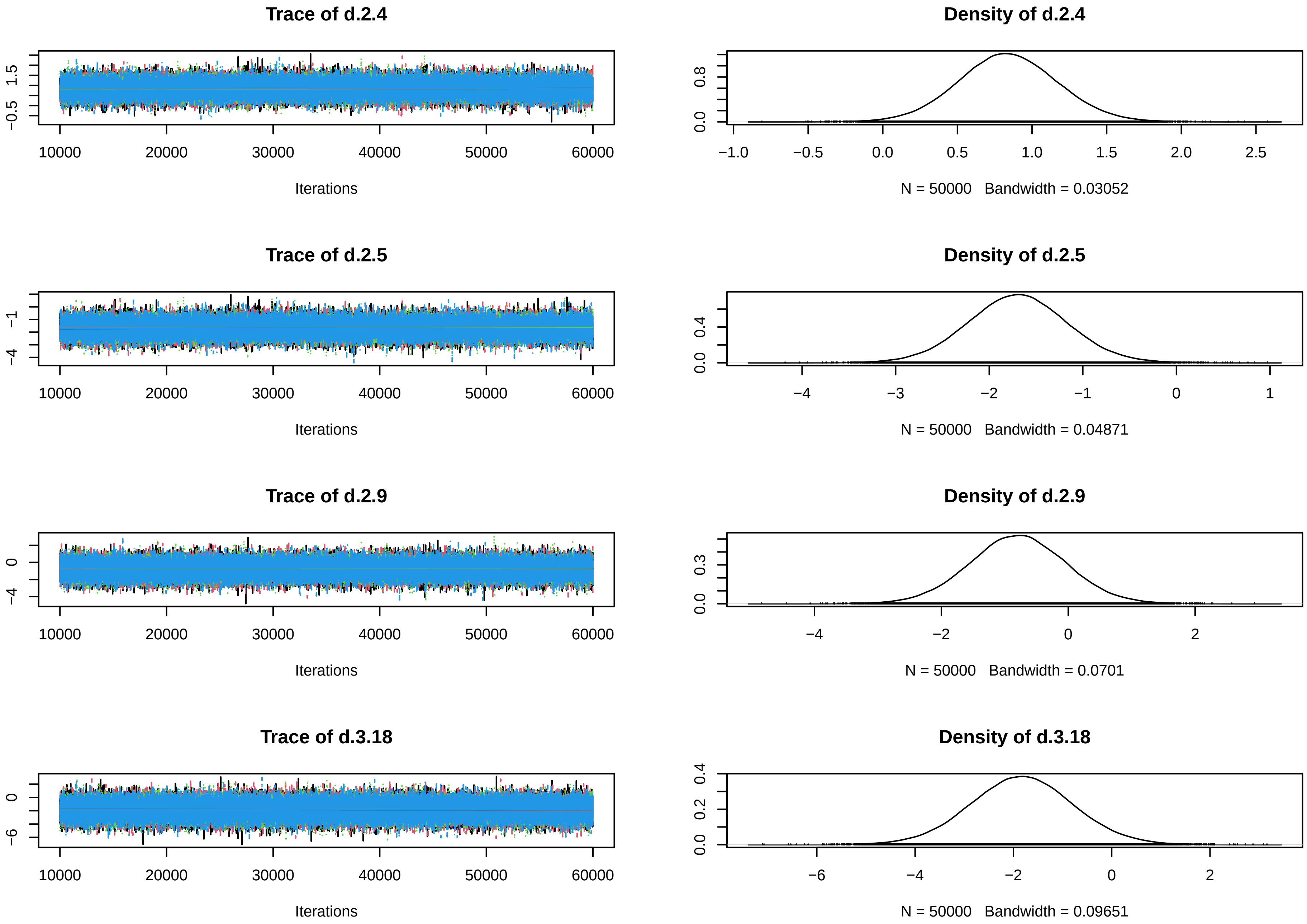
Trace of d.2.4
−0.5
1.5
10000
20000
30000
40000
50000
60000
Iterations
Density of d.2.4
0.0
0.8
−1.0
−0.5
0.0
0.5
1.0
1.5
2.0
2.5
N = 50000 Bandwidth = 0.03052
Trace of d.2.5
−4
−1
10000
20000
30000
40000
50000
60000
Iterations
Density of d.2.5
0.0
0.4
−4
−3
−2
−1
0
1
N = 50000 Bandwidth = 0.04871
Trace of d.2.9
−4
0
10000
20000
30000
40000
50000
60000
Iterations
Density of d.2.9
0.0
0.3
−4
−2
0
2
N = 50000 Bandwidth = 0.0701
Trace of d.3.18
−6
0
10000
20000
30000
40000
50000
60000
Iterations
Density of d.3.18
0.0
0.2
0.4
−6
−4
−2
0
2
N = 50000 Bandwidth = 0.09651

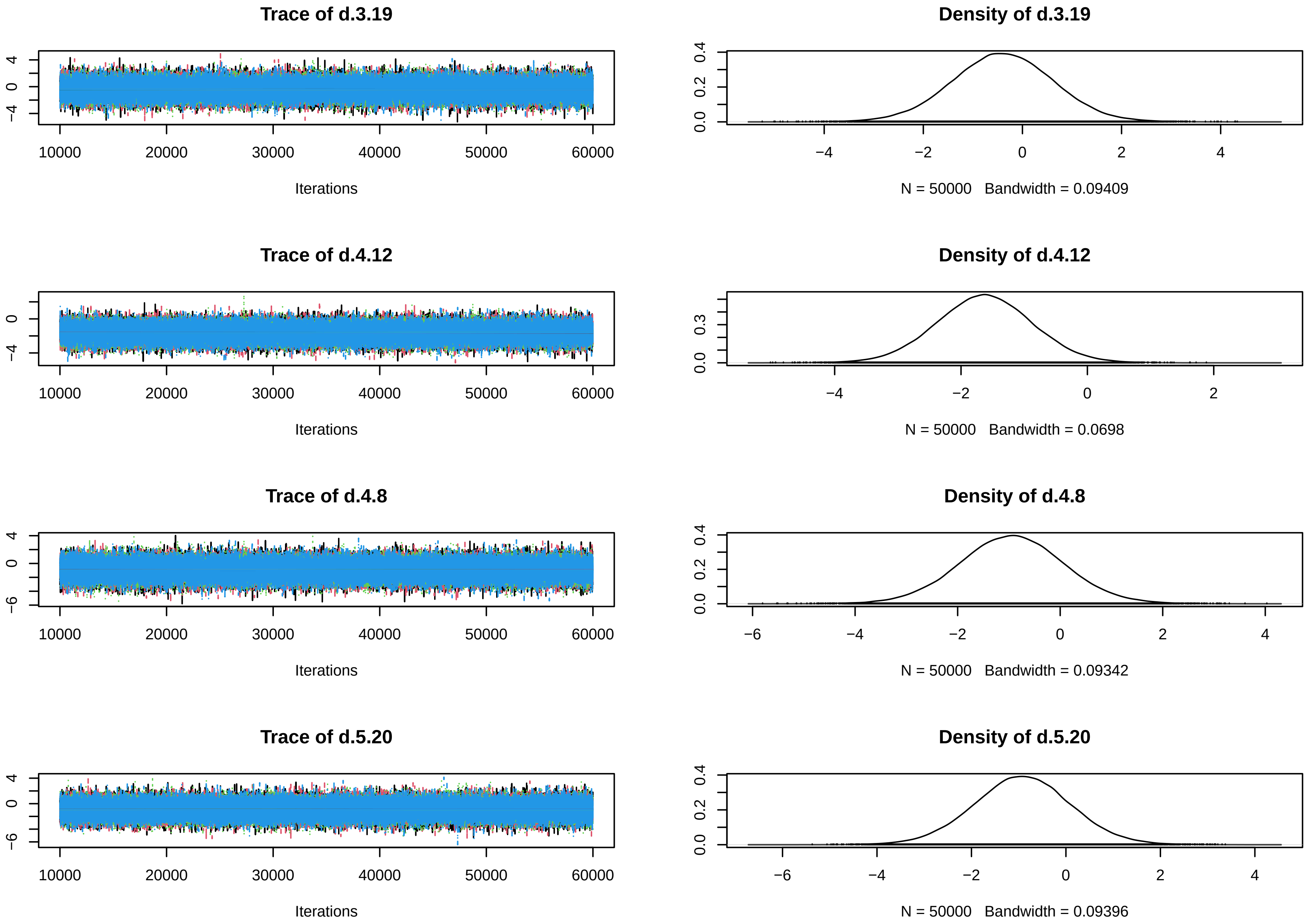
Trace of d.3.19
Iterations
Density of d.3.19
N = 50000   Bandwidth = 0.09409
Trace of d.4.12
Iterations
Density of d.4.12
N = 50000   Bandwidth = 0.0698
Trace of d.4.8
Iterations
Density of d.4.8
N = 50000   Bandwidth = 0.09342
Trace of d.5.20
Iterations
Density of d.5.20
N = 50000   Bandwidth = 0.09396

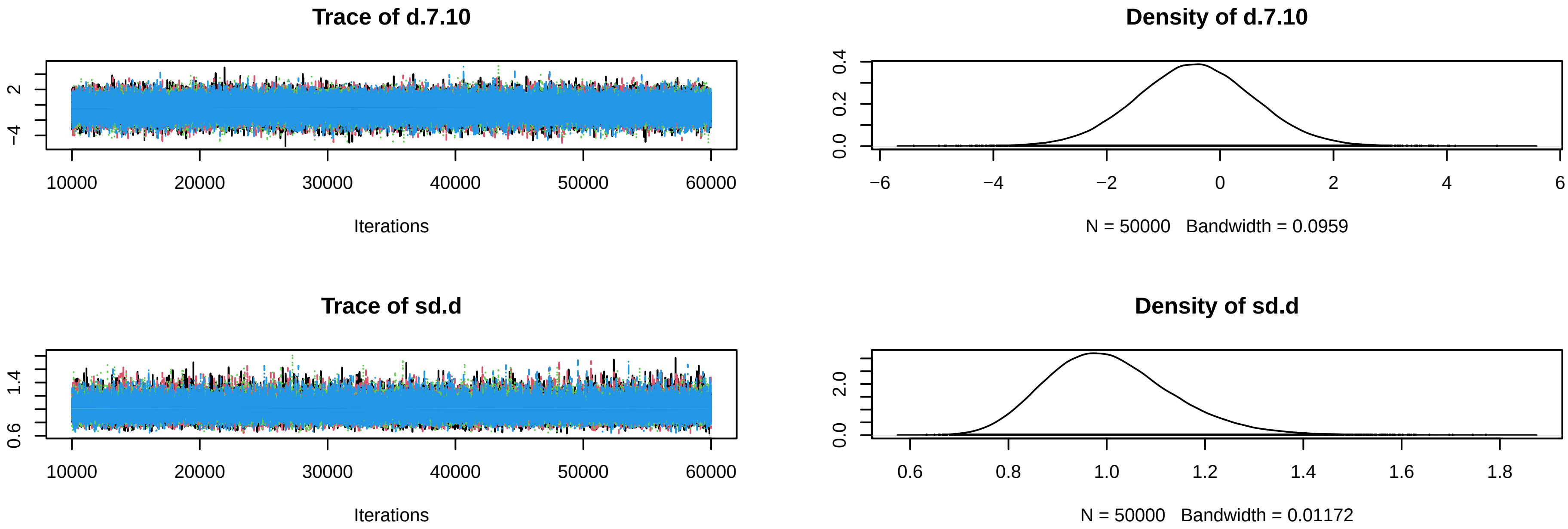

Supplementary Figure 10 Markov chain trace and density plots for the pain relief outcome.
Note: Trace plots (top, iterations vs. parameter value) and density plots (bottom, parameter value vs. density) for key parameters. Well-mixed chains without drift (trace plots) and smooth, unimodal distributions (density plots) indicate successful convergence. The correspondence between intervention codes/abbreviations and their full names is provided in Table S5.
