# Supplementary material for: Comparison of efficacy and safety of non-oral therapeutic interventions for zoster-associated pain: a systematic review and network meta-analysis
Source: Front Neurol. 2026 Jan 27;17:1711536. doi: 10.3389/fneur.2026.1711536 (PMC12886049; doi:10.3389/fneur.2026.1711536)
Supplement: Supplementary file 1 [file Data_Sheet_1.zip › Supplementary_Material_Complete/Data Sheet 11.pdf]

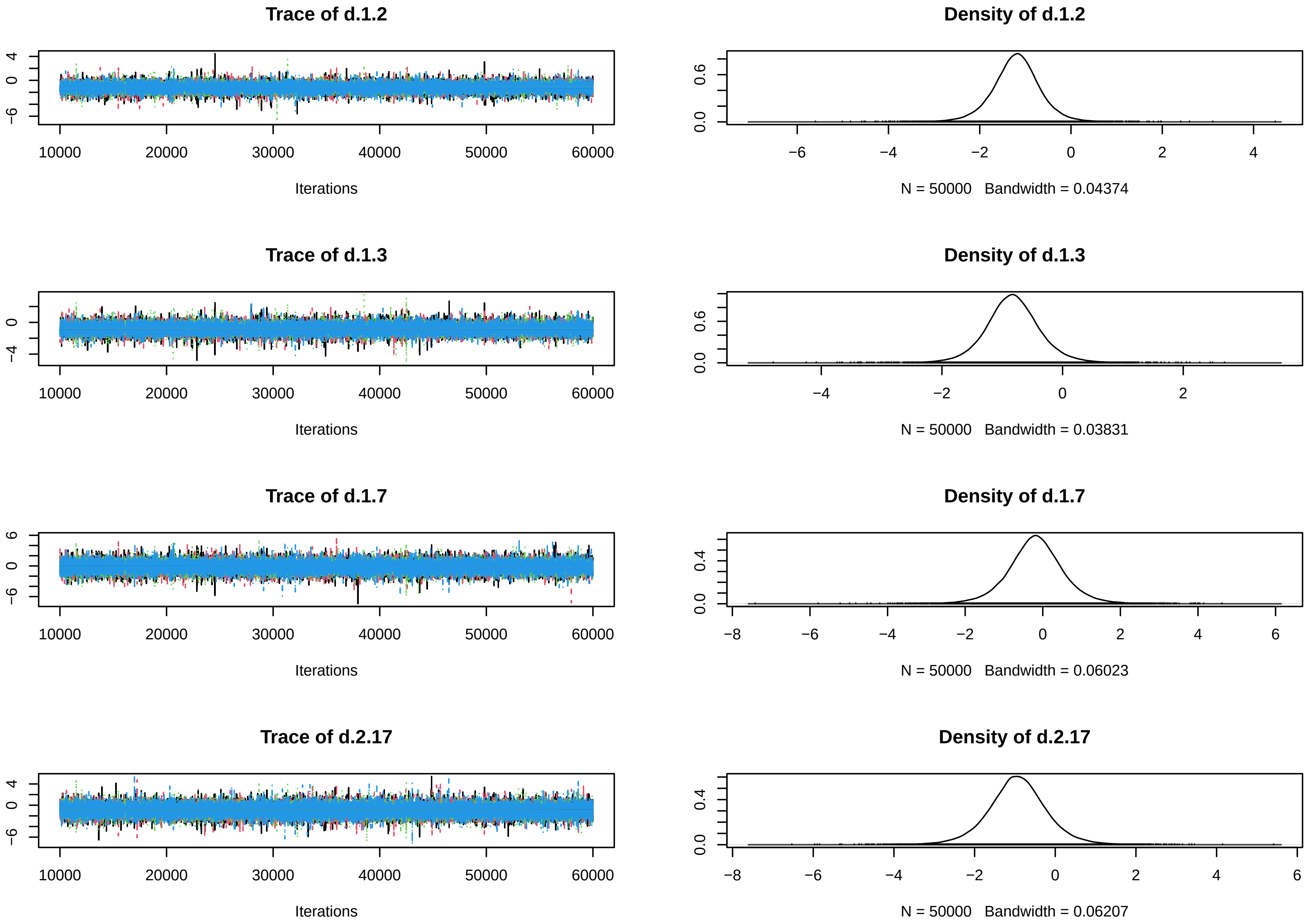

Trace of d.1.2
Iterations
Density of d.1.2
N = 50000 Bandwidth = 0.04374
Trace of d.1.3
Iterations
Density of d.1.3
N = 50000 Bandwidth = 0.03831
Trace of d.1.7
Iterations
Density of d.1.7
N = 50000 Bandwidth = 0.06023
Trace of d.2.17
Iterations
Density of d.2.17
N = 50000 Bandwidth = 0.06207

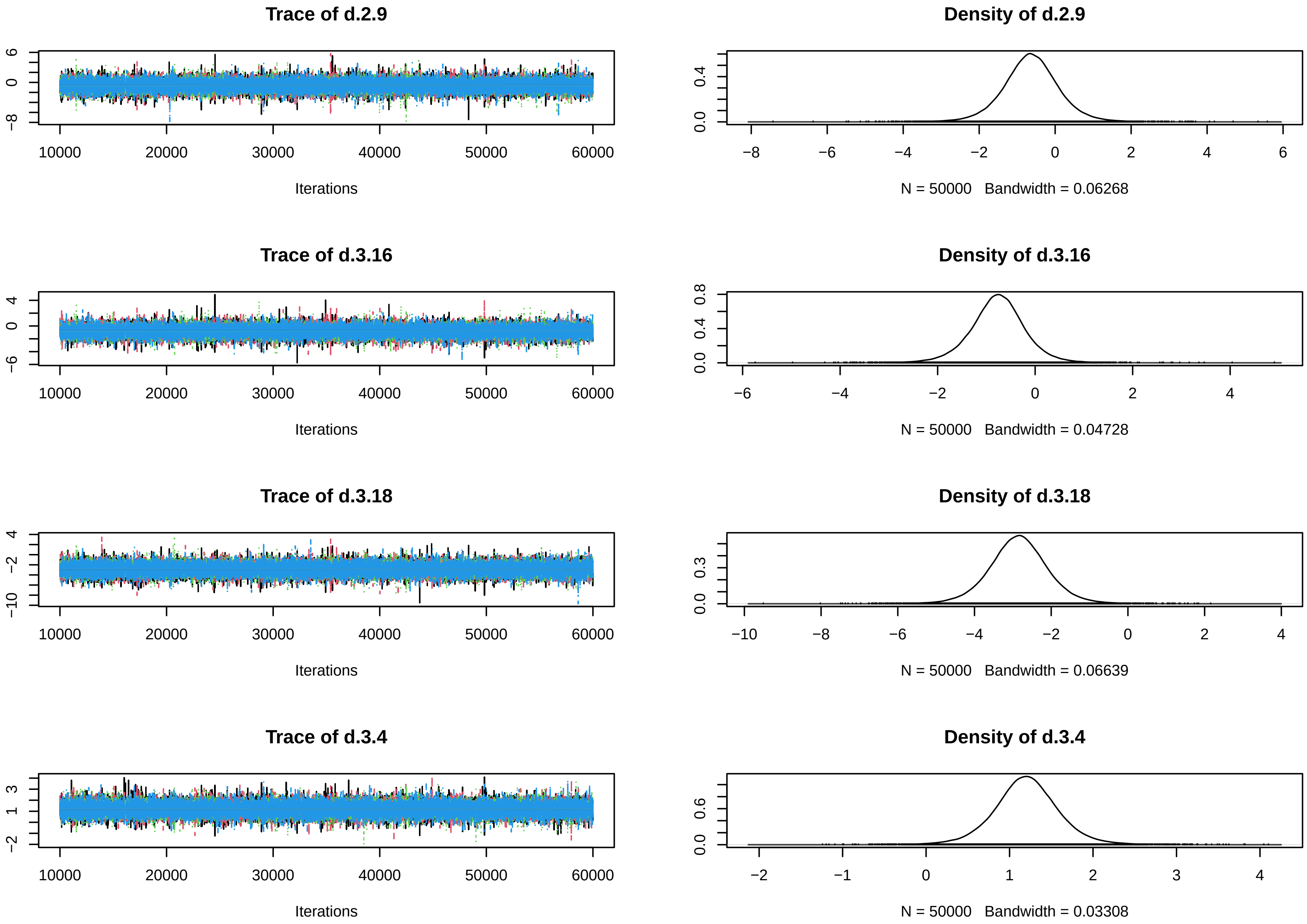

Trace of d.2.9
Iterations
Density of d.2.9
N = 50000   Bandwidth = 0.06268
Trace of d.3.16
Iterations
Density of d.3.16
N = 50000   Bandwidth = 0.04728
Trace of d.3.18
Iterations
Density of d.3.18
N = 50000   Bandwidth = 0.06639
Trace of d.3.4
Iterations
Density of d.3.4
N = 50000   Bandwidth = 0.03308

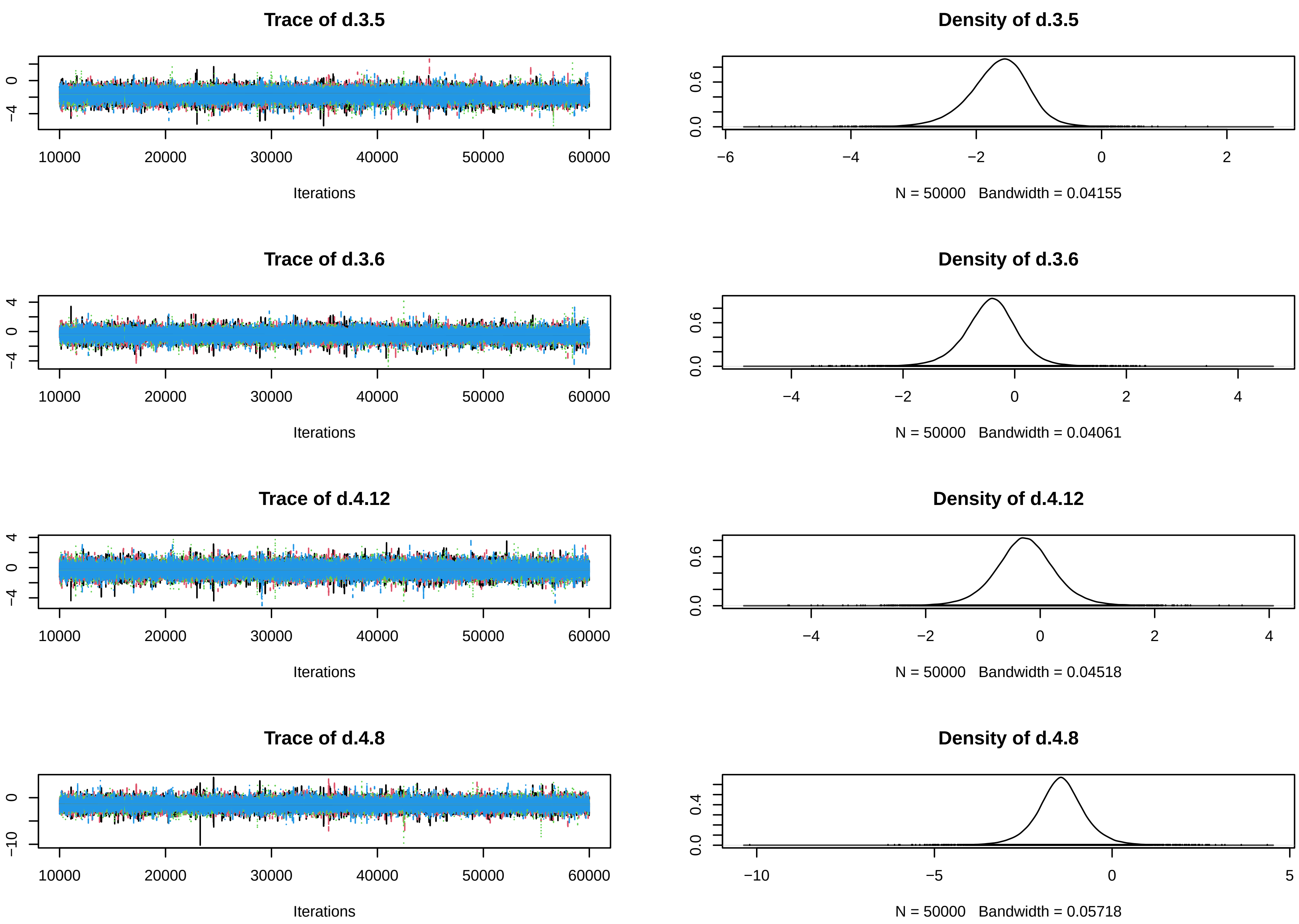
Trace of d.3.5
Iterations
10000 20000 30000 40000 50000 60000
−4 0
Density of d.3.5
N = 50000 Bandwidth = 0.04155
−6 −4 −2 0 2
0.0 0.6
Trace of d.3.6
Iterations
10000 20000 30000 40000 50000 60000
−4 0 4
Density of d.3.6
N = 50000 Bandwidth = 0.04061
−4 −2 0 2 4
0.0 0.6
Trace of d.4.12
Iterations
10000 20000 30000 40000 50000 60000
−4 0 4
Density of d.4.12
N = 50000 Bandwidth = 0.04518
−4 −2 0 2 4
0.0 0.6
Trace of d.4.8
Iterations
10000 20000 30000 40000 50000 60000
−10 0
Density of d.4.8
N = 50000 Bandwidth = 0.05718
−10 −5 0 5
0.0 0.4

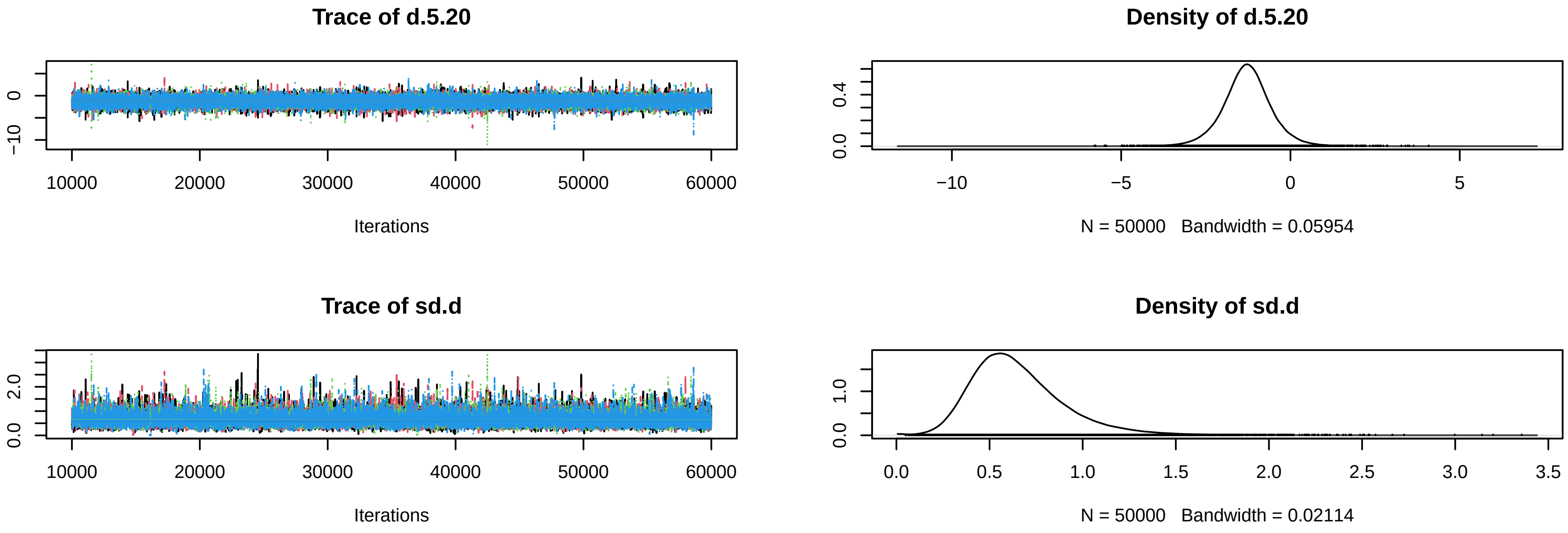

Supplementary Figure 11 Markov chain trace and density plots for the sleep quality outcome.
Note: Trace plots (top, iterations vs. parameter value) and density plots (bottom, parameter value vs. density) for key parameters. Well-mixed chains without drift (trace plots) and smooth, unimodal distributions (density plots) indicate successful convergence. The correspondence between intervention codes/abbreviations and their full names is provided in Table S5.
