# Supplementary material for: Comparison of efficacy and safety of non-oral therapeutic interventions for zoster-associated pain: a systematic review and network meta-analysis
Source: Front Neurol. 2026 Jan 27;17:1711536. doi: 10.3389/fneur.2026.1711536 (PMC12886049; doi:10.3389/fneur.2026.1711536)
Supplement: Supplementary file 1 [file Data_Sheet_1.zip › Supplementary_Material_Complete/Data Sheet 12.pdf]

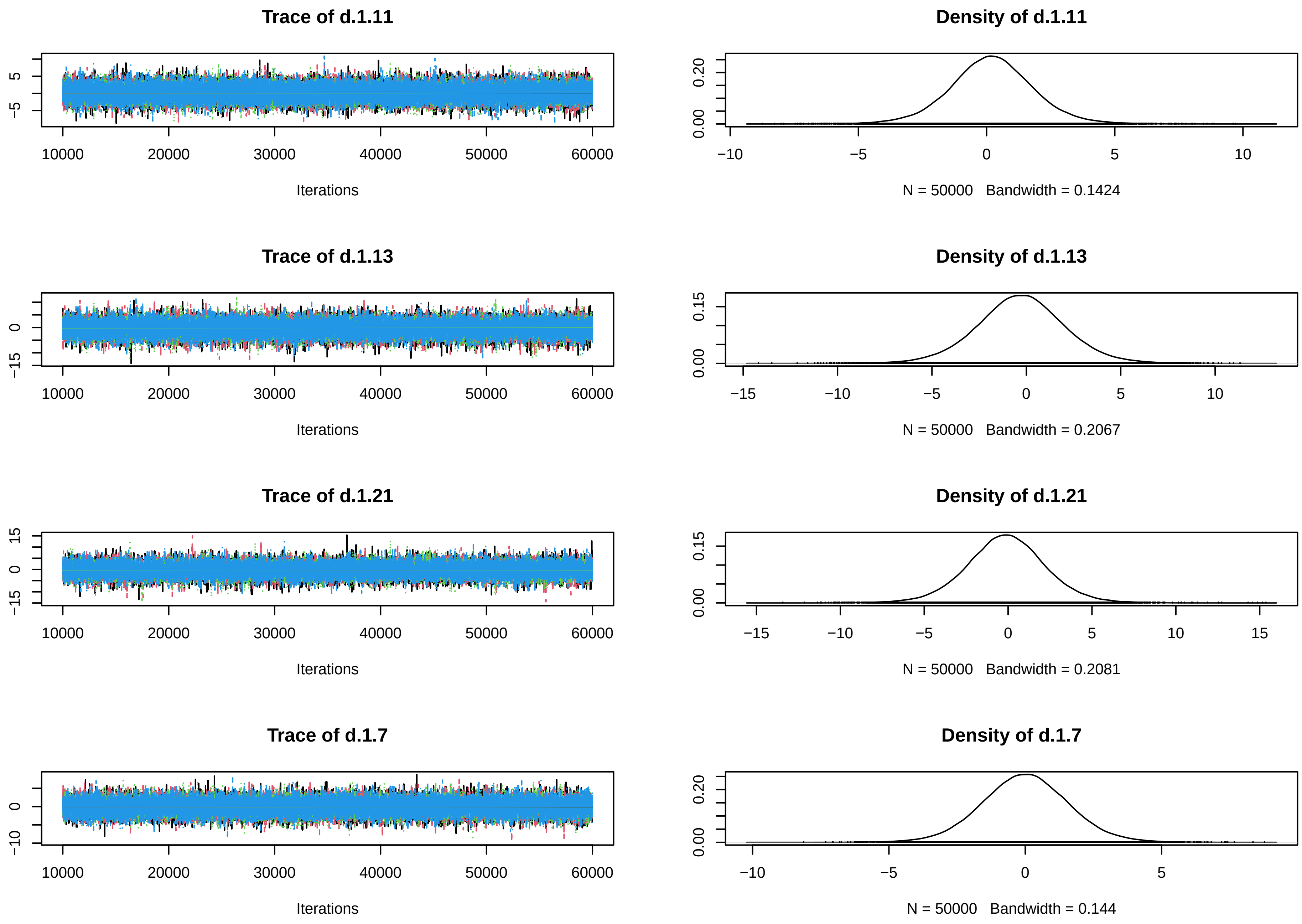

Trace of d.1.11
Iterations
Density of d.1.11
N = 50000 Bandwidth = 0.1424
Trace of d.1.13
Iterations
Density of d.1.13
N = 50000 Bandwidth = 0.2067
Trace of d.1.21
Iterations
Density of d.1.21
N = 50000 Bandwidth = 0.2081
Trace of d.1.7
Iterations
Density of d.1.7
N = 50000 Bandwidth = 0.144

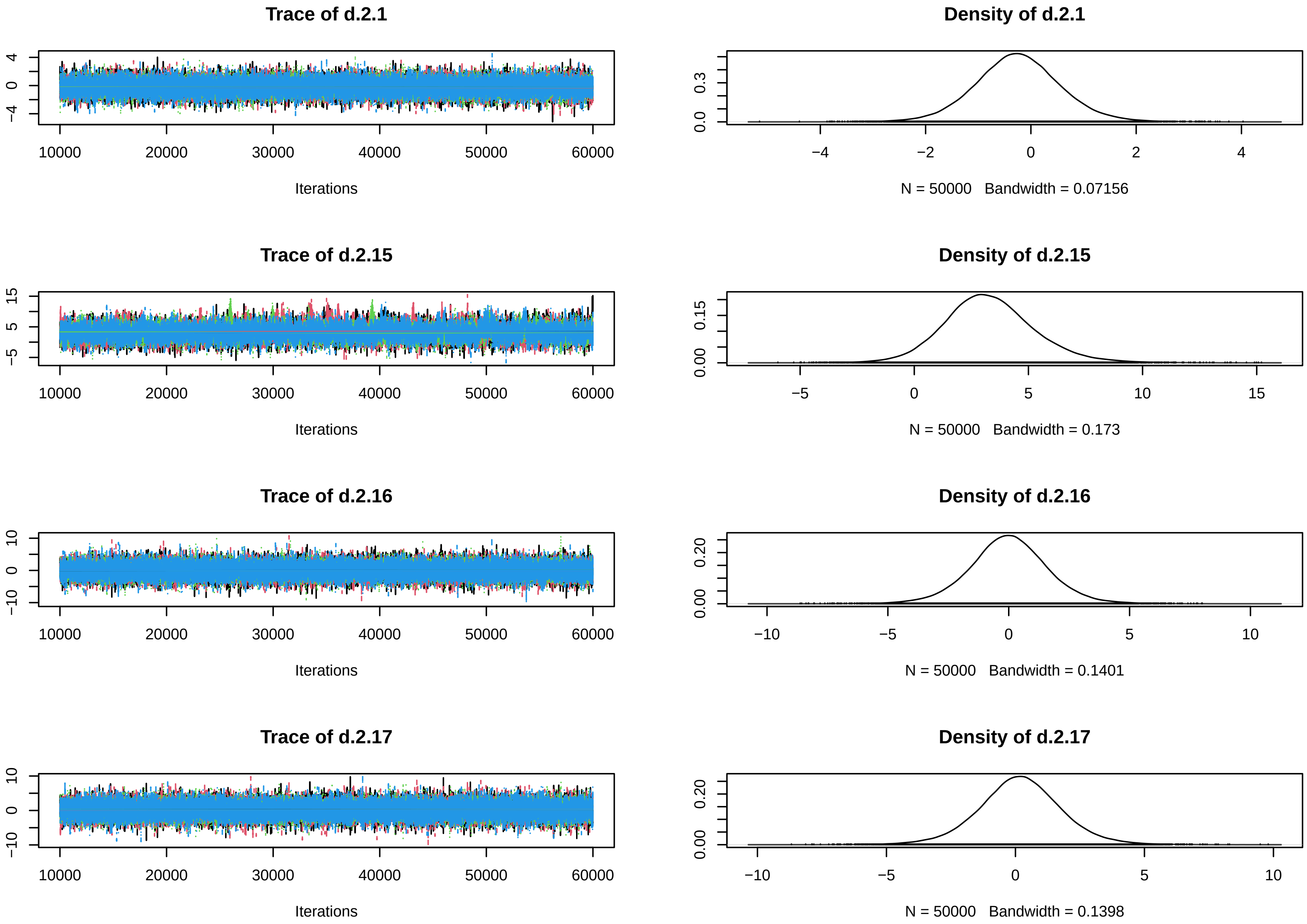
Trace of d.2.1
−4 0 4
10000 20000 30000 40000 50000 60000
Iterations
Density of d.2.1
0.0 0.3
−4 −2 0 2 4
N = 50000 Bandwidth = 0.07156
Trace of d.2.15
−5 5 15
10000 20000 30000 40000 50000 60000
Iterations
Density of d.2.15
0.00 0.15
−5 0 5 10 15
N = 50000 Bandwidth = 0.173
Trace of d.2.16
−10 0 10
10000 20000 30000 40000 50000 60000
Iterations
Density of d.2.16
0.00 0.20
−10 −5 0 5 10
N = 50000 Bandwidth = 0.1401
Trace of d.2.17
−10 0 10
10000 20000 30000 40000 50000 60000
Iterations
Density of d.2.17
0.00 0.20
−10 −5 0 5 10
N = 50000 Bandwidth = 0.1398

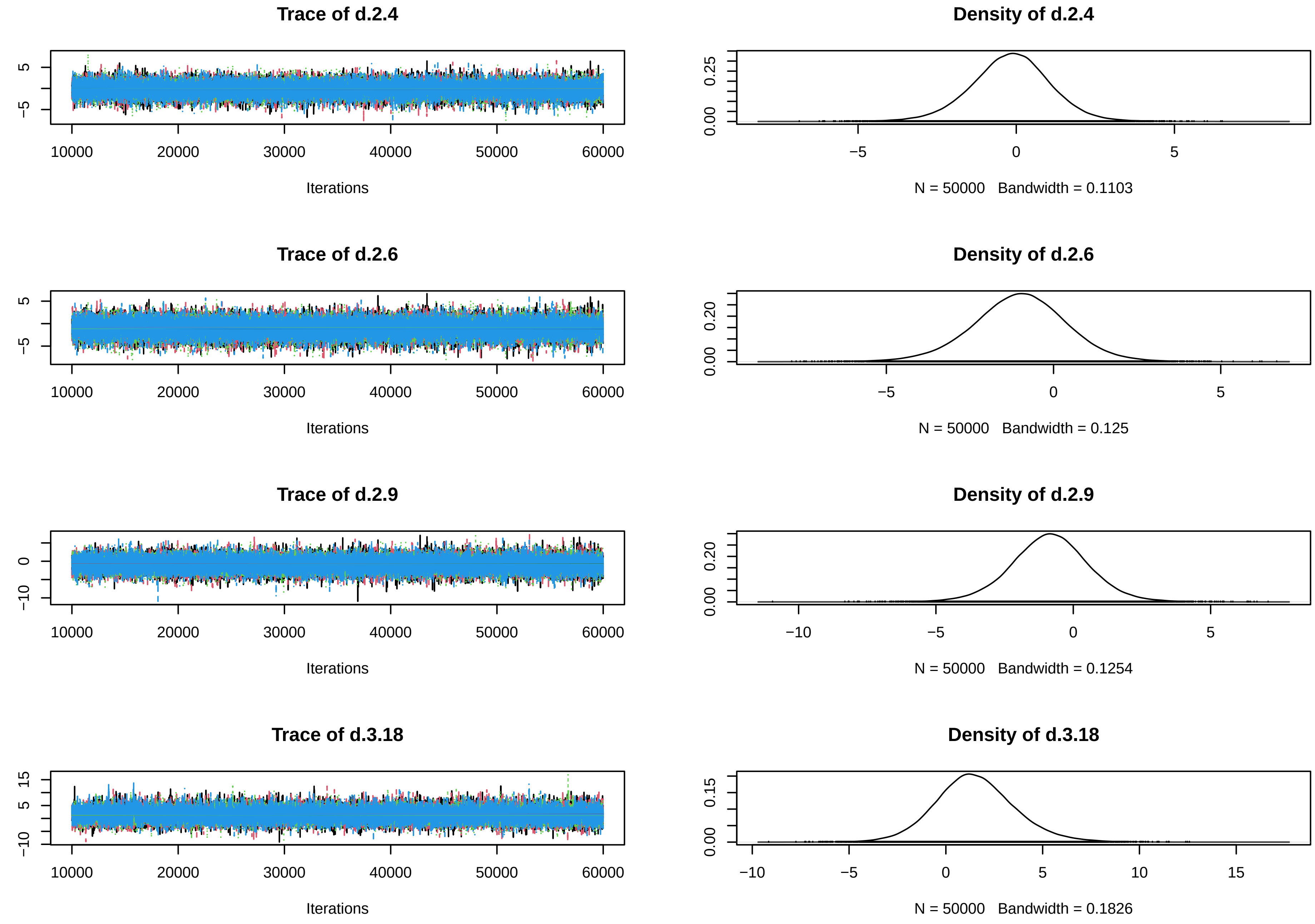
Trace of d.2.4
Iterations
Density of d.2.4
N = 50000 Bandwidth = 0.1103
Trace of d.2.6
Iterations
Density of d.2.6
N = 50000 Bandwidth = 0.125
Trace of d.2.9
Iterations
Density of d.2.9
N = 50000 Bandwidth = 0.1254
Trace of d.3.18
Iterations
Density of d.3.18
N = 50000 Bandwidth = 0.1826

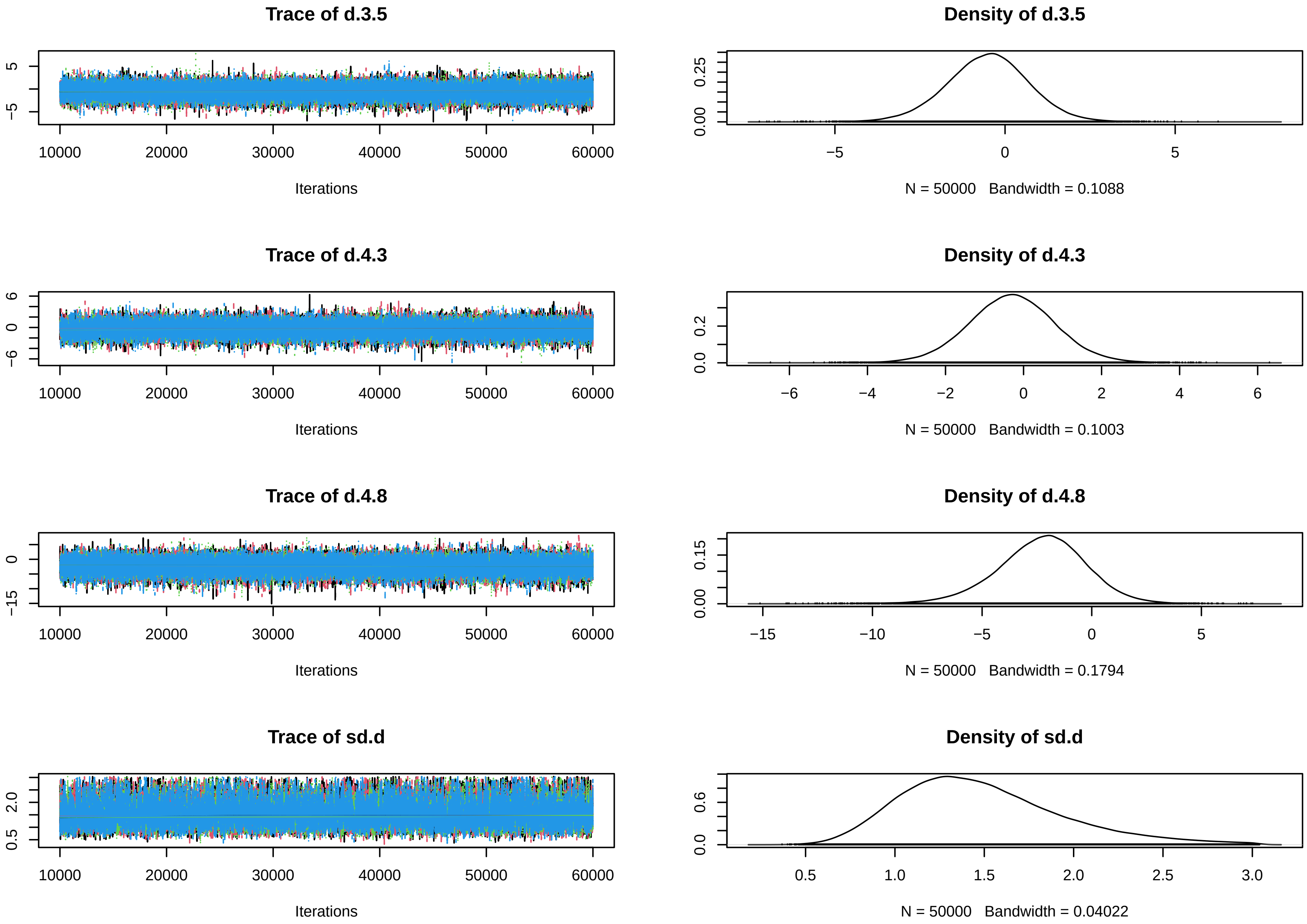
Trace of d.3.5
Density of d.3.5
N = 50000 Bandwidth = 0.1088
Trace of d.4.3
Density of d.4.3
N = 50000 Bandwidth = 0.1003
Trace of d.4.8
Density of d.4.8
N = 50000 Bandwidth = 0.1794
Trace of sd.d
Density of sd.d
N = 50000 Bandwidth = 0.04022
Iterations

Supplementary Figure 12 Markov chain trace and density plots for the adverse events outcome.
Note: Trace plots (top, iterations vs. parameter value) and density plots (bottom, parameter value vs. density) for key parameters. Well-mixed chains without drift (trace plots) and smooth, unimodal distributions (density plots) indicate successful convergence. The correspondence between intervention codes/abbreviations and their full names is provided in Table S5.
