# Supplementary material for: Comparison of efficacy and safety of non-oral therapeutic interventions for zoster-associated pain: a systematic review and network meta-analysis
Source: Front Neurol. 2026 Jan 27;17:1711536. doi: 10.3389/fneur.2026.1711536 (PMC12886049; doi:10.3389/fneur.2026.1711536)
Supplement: Supplementary file 1 [file Data_Sheet_1.zip › Supplementary_Material_Complete/Data Sheet 13.docx]

**File 1** Search strategy

**The search strategy (Pubmed)**

| Search number | Query | Results |
| --- | --- | --- |
| 1 | Herpes Zoster[MeSH Terms] | 13919 |
| 2 | "disseminated herpes zoster"[Title/Abstract] OR "herpes zona"[Title/Abstract] OR "Herpes Zoster"[Title/Abstract] OR "herpes zoster disease"[Title/Abstract] OR "herpes zoster infection"[Title/Abstract] OR "herpes zoster neuralgia"[Title/Abstract] OR "herpes zoster paralysis"[Title/Abstract] OR "infection by varicella zoster virus"[Title/Abstract] OR "infection by VZV"[Title/Abstract] OR "infection caused by varicella zoster virus"[Title/Abstract] OR "infection caused by VZV"[Title/Abstract] OR "Shingles"[Title/Abstract] OR "varicella zoster infection"[Title/Abstract] OR "varicella zoster viral infection"[Title/Abstract] OR "Varicella zoster virus infection"[Title/Abstract] OR "Varicellovirus infection"[Title/Abstract] OR "VZV infection"[Title/Abstract] OR "Zona"[Title/Abstract] OR "Zoster"[Title/Abstract] | 37694 |
| 3 | (Pain[MeSH Terms]) OR (Neuralgia[MeSH Terms]) | 489460 |
| 4 | "Ache"[Title/Abstract] OR "Aches"[Title/Abstract] OR "acute pain"[Title/Abstract] OR "alveolar neuralgia"[Title/Abstract] OR "Atypical Neuralgia*"[Title/Abstract] OR "Burning Pain*"[Title/Abstract] OR "cardiac neuralgia"[Title/Abstract] OR "cervico occipital neuralgia"[Title/Abstract] OR "Crushing Pain*"[Title/Abstract] OR "deep pain"[Title/Abstract] OR "degenerative neuralgia"[Title/Abstract] OR "epileptiform neuralgia"[Title/Abstract] OR "hallucinatory neuralgia"[Title/Abstract] OR "idiopathic neuralgia"[Title/Abstract] OR "Iliohypogastric Nerve Neuralgia*"[Title/Abstract] OR "Ilioinguinal Neuralgia*"[Title/Abstract] OR "lightning pain"[Title/Abstract] OR "mammary neuralgia"[Title/Abstract] OR "mandibular joint neuralgia"[Title/Abstract] OR "Migratory Pain*"[Title/Abstract] OR "nasociliary neuralgia"[Title/Abstract] OR "Nerve Pain*"[Title/Abstract] OR "Neuralgia*"[Title/Abstract] OR "neuralgic pain"[Title/Abstract] OR "neuralgy"[Title/Abstract] OR "Neurodynia*"[Title/Abstract] OR "neurologic pain"[Title/Abstract] OR "neurological pain"[Title/Abstract] OR "Neuropathic Pain*"[Title/Abstract] OR "nocturnal pain"[Title/Abstract] OR "occipital neuralgia"[Title/Abstract] OR "Pain"[Title/Abstract] OR "pain response"[Title/Abstract] OR "pain syndrome"[Title/Abstract] OR "Paroxysmal Nerve Pain*"[Title/Abstract] OR "Perineal Neuralgia*"[Title/Abstract] OR "peripheral neuralgia"[Title/Abstract] OR "pharyngeal neuralgia"[Title/Abstract] OR "Physical Suffering*"[Title/Abstract] OR "Radiating Pain*"[Title/Abstract] OR "red neuralgia"[Title/Abstract] OR "reminiscent neuralgia"[Title/Abstract] OR "rheumatic neuralgia"[Title/Abstract] OR "segmental neuralgia"[Title/Abstract] OR "sphenopalatine neuralgia"[Title/Abstract] OR "Splitting Pain*"[Title/Abstract] OR "Stump Neuralgia*"[Title/Abstract] OR "Supraorbital Neuralgia*"[Title/Abstract] OR "symptomatic neuralgia"[Title/Abstract] OR "treatment related pain"[Title/Abstract] OR "trifacial neuralgia"[Title/Abstract] OR "Vidian Neuralgia*"[Title/Abstract] OR "visceral neuralgia"[Title/Abstract] | 904378 |
| 5 | (#1 OR #2) AND (#3 OR #4) | 4655 |

**The search strategy (embase)**

| Search number | Query | Results |
| --- | --- | --- |
| 1 | 'herpes zoster'/exp | 36157 |
| 2 | 'disseminated herpes zoster':ab,ti,kw OR 'herpes zona':ab,ti,kw OR 'herpes zoster':ab,ti,kw OR 'herpes zoster disease':ab,ti,kw OR 'herpes zoster infection':ab,ti,kw OR 'herpes zoster neuralgia':ab,ti,kw OR 'herpes zoster paralysis':ab,ti,kw OR 'infection by varicella zoster virus':ab,ti,kw OR 'infection by vzv':ab,ti,kw OR 'infection caused by varicella zoster virus':ab,ti,kw OR 'infection caused by vzv':ab,ti,kw OR 'shingles':ab,ti,kw OR 'varicella zoster infection':ab,ti,kw OR 'varicella zoster viral infection':ab,ti,kw OR 'varicella zoster virus infection':ab,ti,kw OR 'varicellovirus infection':ab,ti,kw OR 'vzv infection':ab,ti,kw OR 'zona':ab,ti,kw OR 'zoster':ab,ti,kw | 50149 |
| 3 | 'pain'/exp | 1948488 |
| 4 | 'neuralgia'/exp | 151106 |
| 5 | 'ache':ab,ti,kw OR 'aches':ab,ti,kw OR 'acute pain':ab,ti,kw OR 'alveolar neuralgia':ab,ti,kw OR 'atypical neuralgia*':ab,ti,kw OR 'burning pain*':ab,ti,kw OR 'cardiac neuralgia':ab,ti,kw OR 'cervico occipital neuralgia':ab,ti,kw OR 'crushing pain*':ab,ti,kw OR 'deep pain':ab,ti,kw OR 'degenerative neuralgia':ab,ti,kw OR 'epileptiform neuralgia':ab,ti,kw OR 'hallucinatory neuralgia':ab,ti,kw OR 'idiopathic neuralgia':ab,ti,kw OR 'iliohypogastric nerve neuralgia*':ab,ti,kw OR 'ilioinguinal neuralgia*':ab,ti,kw OR 'lightning pain':ab,ti,kw OR 'mammary neuralgia':ab,ti,kw OR 'mandibular joint neuralgia':ab,ti,kw OR 'migratory pain*':ab,ti,kw OR 'nasociliary neuralgia':ab,ti,kw OR 'nerve pain*':ab,ti,kw OR 'neuralgia*':ab,ti,kw OR 'neuralgic pain':ab,ti,kw OR 'neuralgy':ab,ti,kw OR 'neurodynia*':ab,ti,kw OR 'neurologic pain':ab,ti,kw OR 'neurological pain':ab,ti,kw OR 'neuropathic pain*':ab,ti,kw OR 'nocturnal pain':ab,ti,kw OR 'occipital neuralgia':ab,ti,kw OR 'pain':ab,ti,kw OR 'pain response':ab,ti,kw OR 'pain syndrome':ab,ti,kw OR 'paroxysmal nerve pain*':ab,ti,kw OR 'perineal neuralgia*':ab,ti,kw OR 'peripheral neuralgia':ab,ti,kw OR 'pharyngeal neuralgia':ab,ti,kw OR 'physical suffering*':ab,ti,kw OR 'radiating pain*':ab,ti,kw OR 'red neuralgia':ab,ti,kw OR 'reminiscent neuralgia':ab,ti,kw OR 'rheumatic neuralgia':ab,ti,kw OR 'segmental neuralgia':ab,ti,kw OR 'sphenopalatine neuralgia':ab,ti,kw OR 'splitting pain*':ab,ti,kw OR 'stump neuralgia*':ab,ti,kw OR 'supraorbital neuralgia*':ab,ti,kw OR 'symptomatic neuralgia':ab,ti,kw OR 'treatment related pain':ab,ti,kw OR 'trifacial neuralgia':ab,ti,kw OR 'vidian neuralgia*':ab,ti,kw OR 'visceral neuralgia':ab,ti,kw | 1380982 |
| 6 | (#1 OR #2) AND (#3 OR #4 OR #5) | 14384 |

**The search strategy (Cochrane)**

| Search number | Query | Results |
| --- | --- | --- |
| 1 | MeSH descriptor: [Herpes Zoster] explode all trees | 798 |
| 2 | ('disseminated herpes zoster' OR 'herpes zona' OR 'Herpes Zoster' OR 'herpes zoster disease' OR 'herpes zoster infection' OR 'herpes zoster neuralgia' OR 'herpes zoster paralysis' OR 'infection by varicella zoster virus' OR 'infection by VZV' OR 'infection caused by varicella zoster virus' OR 'infection caused by VZV' OR 'Shingles' OR 'varicella zoster infection' OR 'varicella zoster viral infection' OR 'Varicella zoster virus infection' OR 'Varicellovirus infection' OR 'VZV infection' OR 'Zona' OR 'Zoster'):ab,ti,kw | 3521 |
| 3 | MeSH descriptor: [Pain] explode all trees | 73001 |
| 4 | MeSH descriptor: [Neuralgia] explode all trees | 2502 |
| 5 | ('Ache' OR 'Aches' OR 'acute pain' OR 'alveolar neuralgia' OR 'Atypical Neuralgia*' OR 'Burning Pain*' OR 'cardiac neuralgia' OR 'cervico occipital neuralgia' OR 'Crushing Pain*' OR 'deep pain' OR 'degenerative neuralgia' OR 'epileptiform neuralgia' OR 'hallucinatory neuralgia' OR 'idiopathic neuralgia' OR 'Iliohypogastric Nerve Neuralgia*' OR 'Ilioinguinal Neuralgia*' OR 'lightning pain' OR 'mammary neuralgia' OR 'mandibular joint neuralgia' OR 'Migratory Pain*' OR 'nasociliary neuralgia' OR 'Nerve Pain*' OR 'Neuralgia*' OR 'neuralgic pain' OR 'neuralgy' OR 'Neurodynia*' OR 'neurologic pain' OR 'neurological pain' OR 'Neuropathic Pain*' OR 'nocturnal pain' OR 'occipital neuralgia' OR 'Pain' OR 'pain response' OR 'pain syndrome' OR 'Paroxysmal Nerve Pain*' OR 'Perineal Neuralgia*' OR 'peripheral neuralgia' OR 'pharyngeal neuralgia' OR 'Physical Suffering*' OR 'Radiating Pain*' OR 'red neuralgia' OR 'reminiscent neuralgia' OR 'rheumatic neuralgia' OR 'segmental neuralgia' OR 'sphenopalatine neuralgia' OR 'Splitting Pain*' OR 'Stump Neuralgia*' OR 'Supraorbital Neuralgia*' OR 'symptomatic neuralgia' OR 'treatment related pain' OR 'trifacial neuralgia' OR 'Vidian Neuralgia*' OR 'visceral neuralgia'):ab,ti,kw | 269545 |
| 6 | (#1 OR #2) AND (#3 OR #4 OR #5) | 1233 |

**The search strategy (Web of Science)**

| Search number | Query | Results |
| --- | --- | --- |
| 1 | TS=((disseminated herpes zoster) OR (herpes zona) OR (Herpes Zoster) OR (herpes zoster disease) OR (herpes zoster infection) OR (herpes zoster neuralgia) OR (herpes zoster paralysis) OR (infection by varicella zoster virus) OR (infection by VZV) OR (infection caused by varicella zoster virus) OR (infection caused by VZV) OR (Shingles) OR (varicella zoster infection) OR (varicella zoster viral infection) OR (Varicella zoster virus infection) OR (Varicellovirus infection) OR (VZV infection) OR (Zona) OR (Zoster)) and Preprint Citation Index (Exclude – Database) | 100110 |
| 2 | TS=((Ache) OR (Aches) OR (acute pain) OR (alveolar neuralgia) OR (Atypical Neuralgia*) OR (Burning Pain*) OR (cardiac neuralgia) OR (cervico occipital neuralgia) OR (Crushing Pain*) OR (deep pain) OR (degenerative neuralgia) OR (epileptiform neuralgia) OR (hallucinatory neuralgia) OR (idiopathic neuralgia) OR (Iliohypogastric Nerve Neuralgia*) OR (Ilioinguinal Neuralgia*) OR (lightning pain) OR (mammary neuralgia) OR (mandibular joint neuralgia) OR (Migratory Pain*) OR (nasociliary neuralgia) OR (Nerve Pain*) OR (Neuralgia*) OR (neuralgic pain) OR (neuralgy) OR (Neurodynia*) OR (neurologic pain) OR (neurological pain) OR (Neuropathic Pain*) OR (nocturnal pain) OR (occipital neuralgia) OR (Pain) OR (pain response) OR (pain syndrome) OR (Paroxysmal Nerve Pain*) OR (Perineal Neuralgia*) OR (peripheral neuralgia) OR (pharyngeal neuralgia) OR (Physical Suffering*) OR (Radiating Pain*) OR (red neuralgia) OR (reminiscent neuralgia) OR (rheumatic neuralgia) OR (segmental neuralgia) OR (sphenopalatine neuralgia) OR (Splitting Pain*) OR (Stump Neuralgia*) OR (Supraorbital Neuralgia*) OR (symptomatic neuralgia) OR (treatment related pain) OR (trifacial neuralgia) OR (Vidian Neuralgia*) OR (visceral neuralgia)) and Preprint Citation Index (Exclude – Database) | 1368296 |
| 3 | #2 AND #1 and Preprint Citation Index (Exclude – Database) | 7169 |
