# Supplementary material for: Comparison of efficacy and safety of non-oral therapeutic interventions for zoster-associated pain: a systematic review and network meta-analysis
Source: Front Neurol. 2026 Jan 27;17:1711536. doi: 10.3389/fneur.2026.1711536 (PMC12886049; doi:10.3389/fneur.2026.1711536)
Supplement: Supplementary file 1 [file Data_Sheet_1.zip › Supplementary_Material_Complete/Data Sheet 2.pdf]

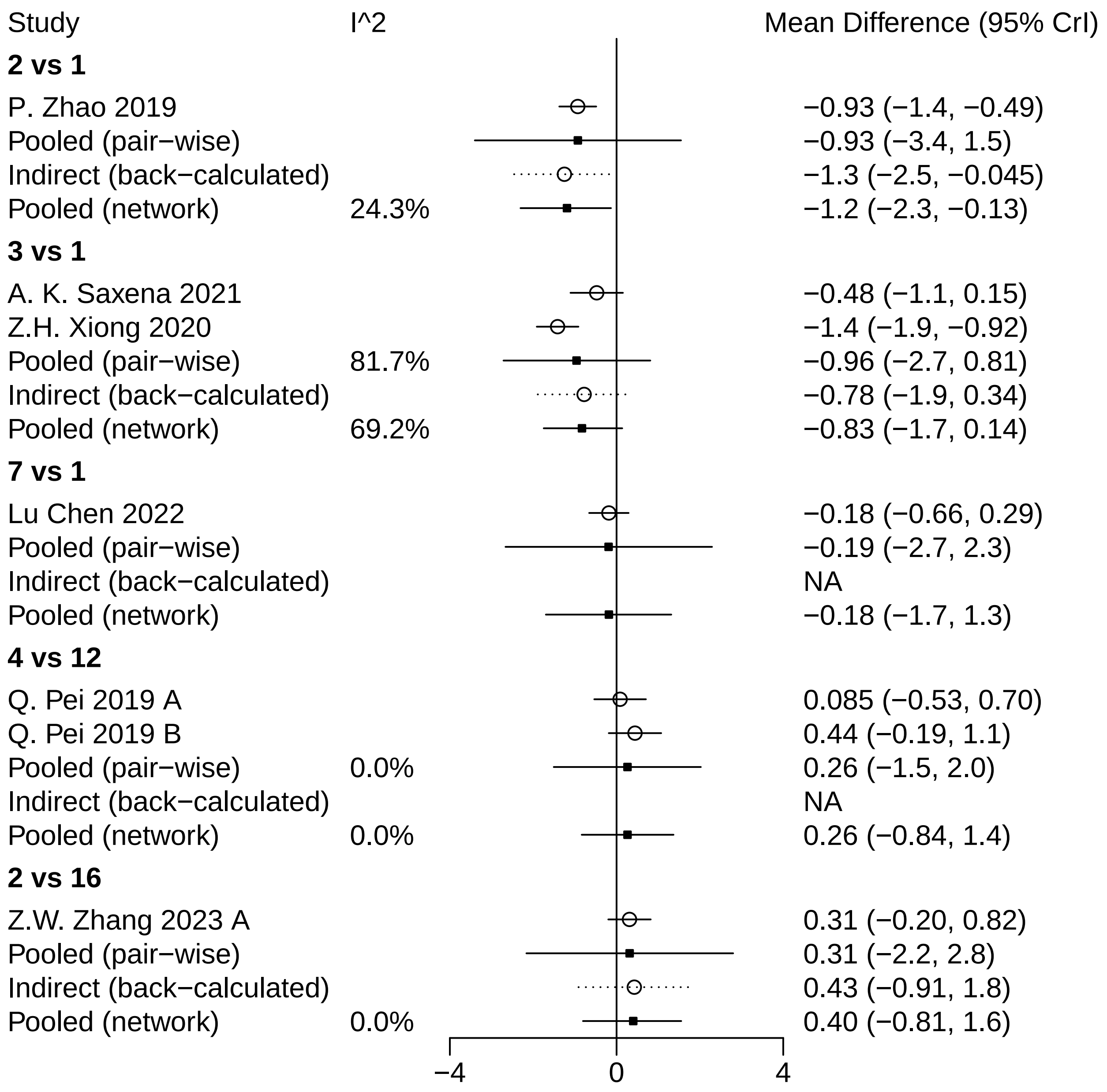
Study
I^2
Mean Difference (95% CrI)
2 vs 1
P. Zhao 2019
−0.93 (−1.4, −0.49)
Pooled (pair−wise)
−0.93 (−3.4, 1.5)
Indirect (back−calculated)
−1.3 (−2.5, −0.045)
Pooled (network)
24.3%
−1.2 (−2.3, −0.13)
3 vs 1
A. K. Saxena 2021
−0.48 (−1.1, 0.15)
Z.H. Xiong 2020
−1.4 (−1.9, −0.92)
Pooled (pair−wise)
81.7%
−0.96 (−2.7, 0.81)
Indirect (back−calculated)
−0.78 (−1.9, 0.34)
Pooled (network)
69.2%
−0.83 (−1.7, 0.14)
7 vs 1
Lu Chen 2022
−0.18 (−0.66, 0.29)
Pooled (pair−wise)
−0.19 (−2.7, 2.3)
Indirect (back−calculated)
NA
Pooled (network)
−0.18 (−1.7, 1.3)
4 vs 12
Q. Pei 2019 A
0.085 (−0.53, 0.70)
Q. Pei 2019 B
0.44 (−0.19, 1.1)
Pooled (pair−wise)
0.0%
0.26 (−1.5, 2.0)
Indirect (back−calculated)
NA
Pooled (network)
0.0%
0.26 (−0.84, 1.4)
2 vs 16
Z.W. Zhang 2023 A
0.31 (−0.20, 0.82)
Pooled (pair−wise)
0.31 (−2.2, 2.8)
Indirect (back−calculated)
0.43 (−0.91, 1.8)
Pooled (network)
0.0%
0.40 (−0.81, 1.6)
−4
0
4

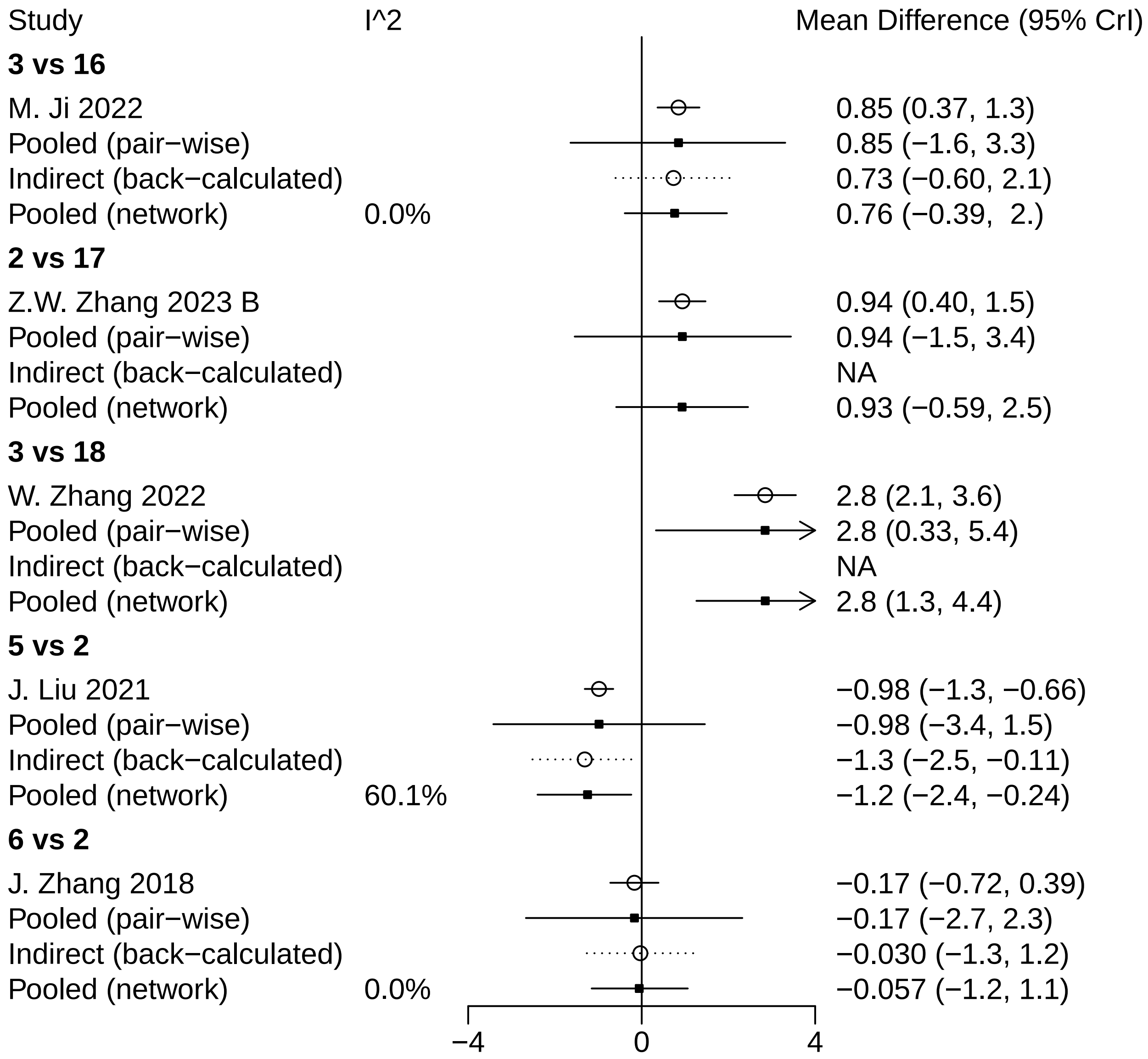
Study
I^2
Mean Difference (95% CrI)
3 vs 16
M. Ji 2022
0.85 (0.37, 1.3)
Pooled (pair−wise)
0.85 (−1.6, 3.3)
Indirect (back−calculated)
0.73 (−0.60, 2.1)
Pooled (network)
0.0%
0.76 (−0.39, 2.)
2 vs 17
Z.W. Zhang 2023 B
0.94 (0.40, 1.5)
Pooled (pair−wise)
0.94 (−1.5, 3.4)
Indirect (back−calculated)
NA
Pooled (network)
0.93 (−0.59, 2.5)
3 vs 18
W. Zhang 2022
2.8 (2.1, 3.6)
Pooled (pair−wise)
2.8 (0.33, 5.4)
Indirect (back−calculated)
NA
Pooled (network)
2.8 (1.3, 4.4)
5 vs 2
J. Liu 2021
−0.98 (−1.3, −0.66)
Pooled (pair−wise)
−0.98 (−3.4, 1.5)
Indirect (back−calculated)
−1.3 (−2.5, −0.11)
Pooled (network)
60.1%
−1.2 (−2.4, −0.24)
6 vs 2
J. Zhang 2018
−0.17 (−0.72, 0.39)
Pooled (pair−wise)
−0.17 (−2.7, 2.3)
Indirect (back−calculated)
−0.030 (−1.3, 1.2)
Pooled (network)
0.0%
−0.057 (−1.2, 1.1)
−4
0
4

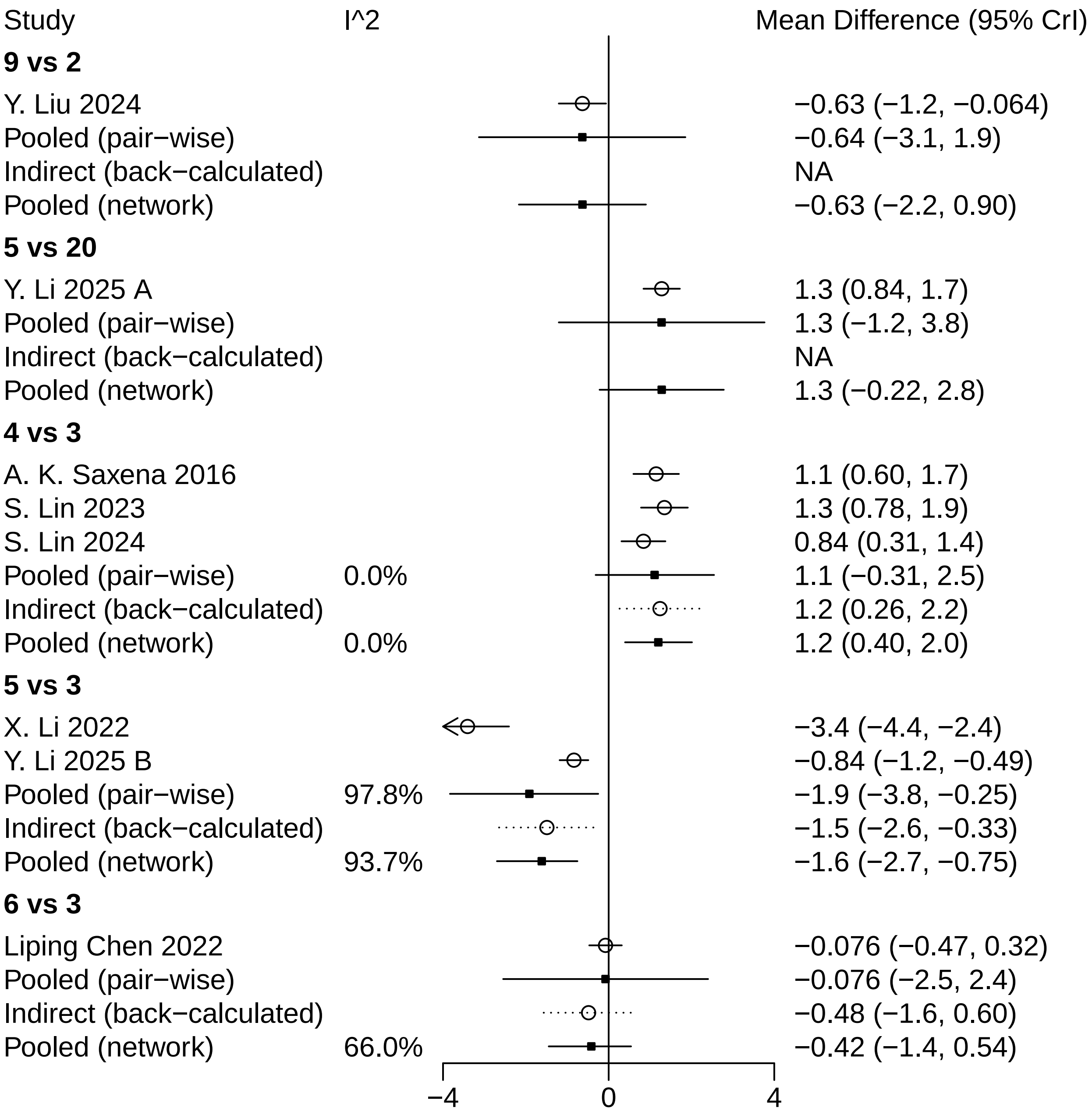

Study
I^2
Mean Difference (95% CrI)
9 vs 2
Y. Liu 2024 −0.63 (−1.2, −0.064)
Pooled (pair−wise) −0.64 (−3.1, 1.9)
Indirect (back−calculated) NA
Pooled (network) −0.63 (−2.2, 0.90)
5 vs 20
Y. Li 2025 A 1.3 (0.84, 1.7)
Pooled (pair−wise) 1.3 (−1.2, 3.8)
Indirect (back−calculated) NA
Pooled (network) 1.3 (−0.22, 2.8)
4 vs 3
A. K. Saxena 2016 1.1 (0.60, 1.7)
S. Lin 2023 1.3 (0.78, 1.9)
S. Lin 2024 0.84 (0.31, 1.4)
Pooled (pair−wise) 0.0% 1.1 (−0.31, 2.5)
Indirect (back−calculated) 1.2 (0.26, 2.2)
Pooled (network) 0.0% 1.2 (0.40, 2.0)
5 vs 3
X. Li 2022 −3.4 (−4.4, −2.4)
Y. Li 2025 B −0.84 (−1.2, −0.49)
Pooled (pair−wise) 97.8% −1.9 (−3.8, −0.25)
Indirect (back−calculated) −1.5 (−2.6, −0.33)
Pooled (network) 93.7% −1.6 (−2.7, −0.75)
6 vs 3
Liping Chen 2022 −0.076 (−0.47, 0.32)
Pooled (pair−wise) −0.076 (−2.5, 2.4)
Indirect (back−calculated) −0.48 (−1.6, 0.60)
Pooled (network) 66.0% −0.42 (−1.4, 0.54)
−4
0
4

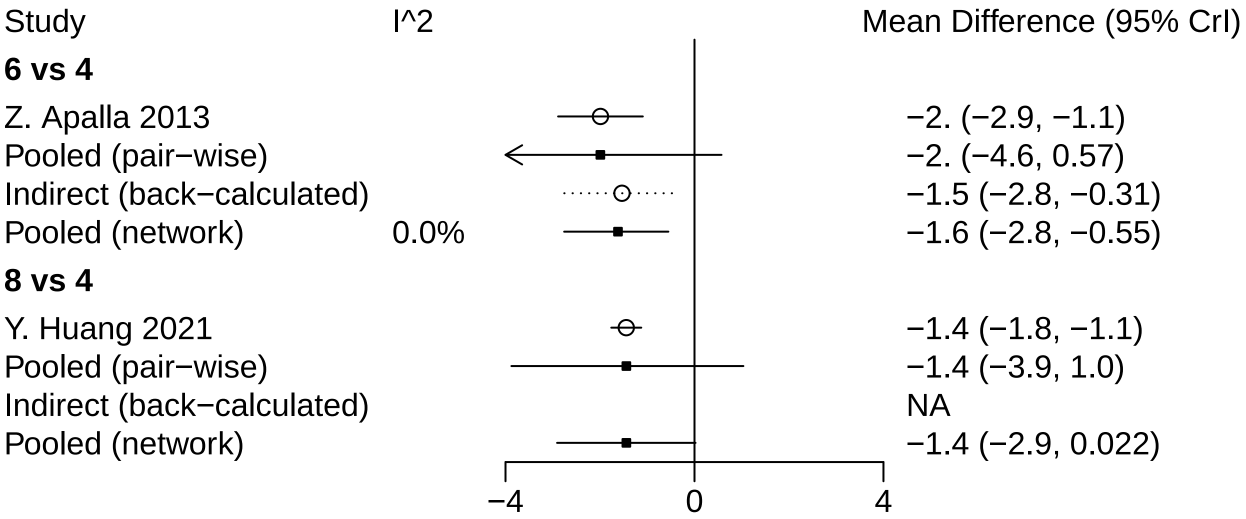

Supplementary Figure 2 Forest plots with heterogeneity assessment for the sleep quality outcome.

Note: This forest plot presents the evidence synthesis results for each direct comparison regarding the sleep quality outcome, based on a Bayesian random-effects model. It displays the direct pairwise meta-analysis results, indirect effect estimates, and the comprehensive network meta-analysis estimates for each comparison. All effect estimates are expressed as mean difference (MD) with 95% credible intervals (95% CrI). The I^2 statistic was used to assess local heterogeneity, with higher values indicating greater heterogeneity. The correspondence between intervention codes/abbreviations and their full names is provided in Table S5.
