# Supplementary material for: Comparison of efficacy and safety of non-oral therapeutic interventions for zoster-associated pain: a systematic review and network meta-analysis
Source: Front Neurol. 2026 Jan 27;17:1711536. doi: 10.3389/fneur.2026.1711536 (PMC12886049; doi:10.3389/fneur.2026.1711536)
Supplement: Supplementary file 1 [file Data_Sheet_1.zip › Supplementary_Material_Complete/Data Sheet 3.pdf]

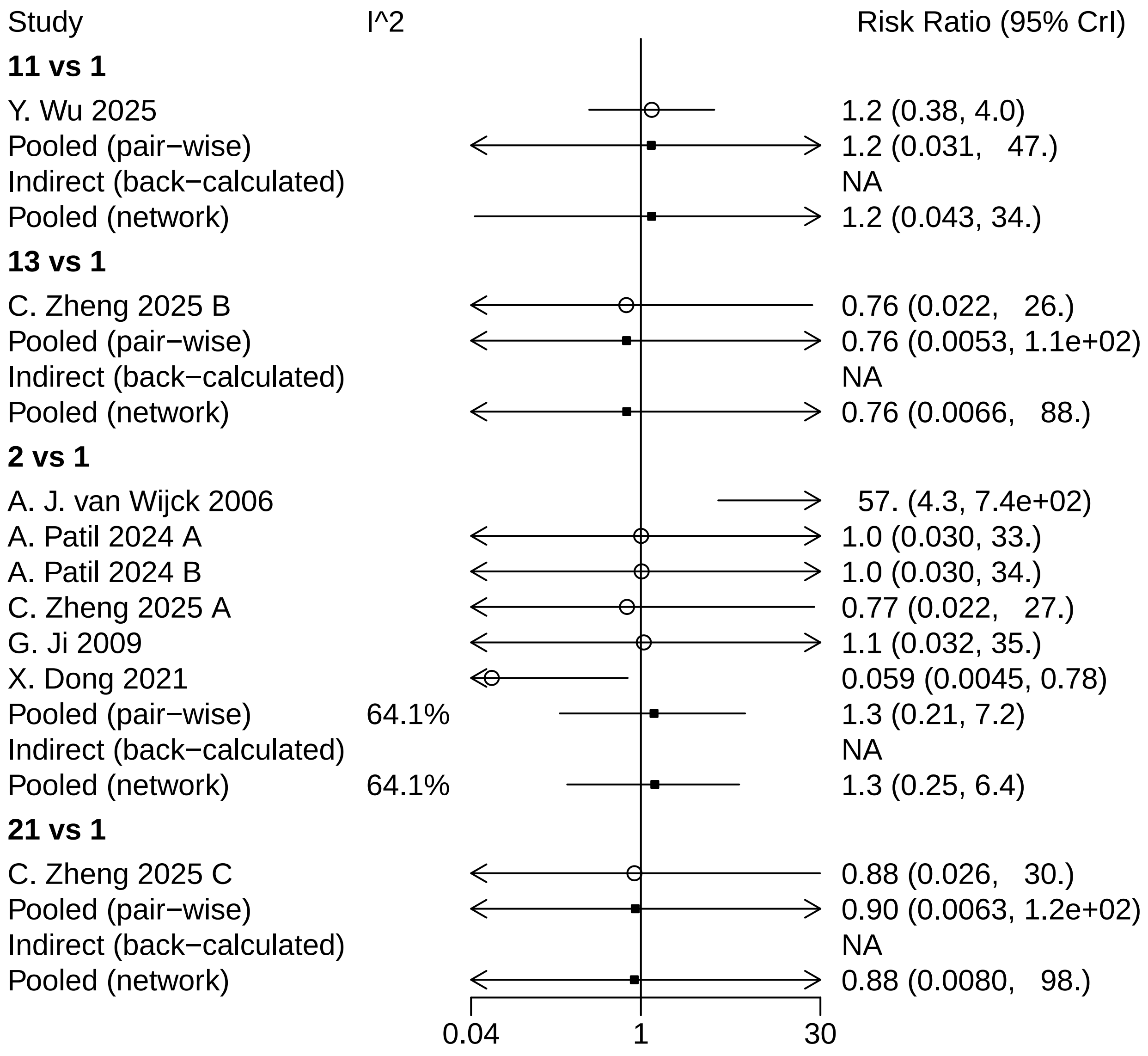
Study
I^2
Risk Ratio (95% CrI)
11 vs 1
Y. Wu 2025
1.2 (0.38, 4.0)
Pooled (pair−wise)
1.2 (0.031, 47.)
Indirect (back−calculated)
NA
Pooled (network)
1.2 (0.043, 34.)
13 vs 1
C. Zheng 2025 B
0.76 (0.022, 26.)
Pooled (pair−wise)
0.76 (0.0053, 1.1e+02)
Indirect (back−calculated)
NA
Pooled (network)
0.76 (0.0066, 88.)
2 vs 1
A. J. van Wijck 2006
57. (4.3, 7.4e+02)
A. Patil 2024 A
1.0 (0.030, 33.)
A. Patil 2024 B
1.0 (0.030, 34.)
C. Zheng 2025 A
0.77 (0.022, 27.)
G. Ji 2009
1.1 (0.032, 35.)
X. Dong 2021
0.059 (0.0045, 0.78)
Pooled (pair−wise)
64.1%
1.3 (0.21, 7.2)
Indirect (back−calculated)
NA
Pooled (network)
64.1%
1.3 (0.25, 6.4)
21 vs 1
C. Zheng 2025 C
0.88 (0.026, 30.)
Pooled (pair−wise)
0.90 (0.0063, 1.2e+02)
Indirect (back−calculated)
NA
Pooled (network)
0.88 (0.0080, 98.)
0.04
1
30

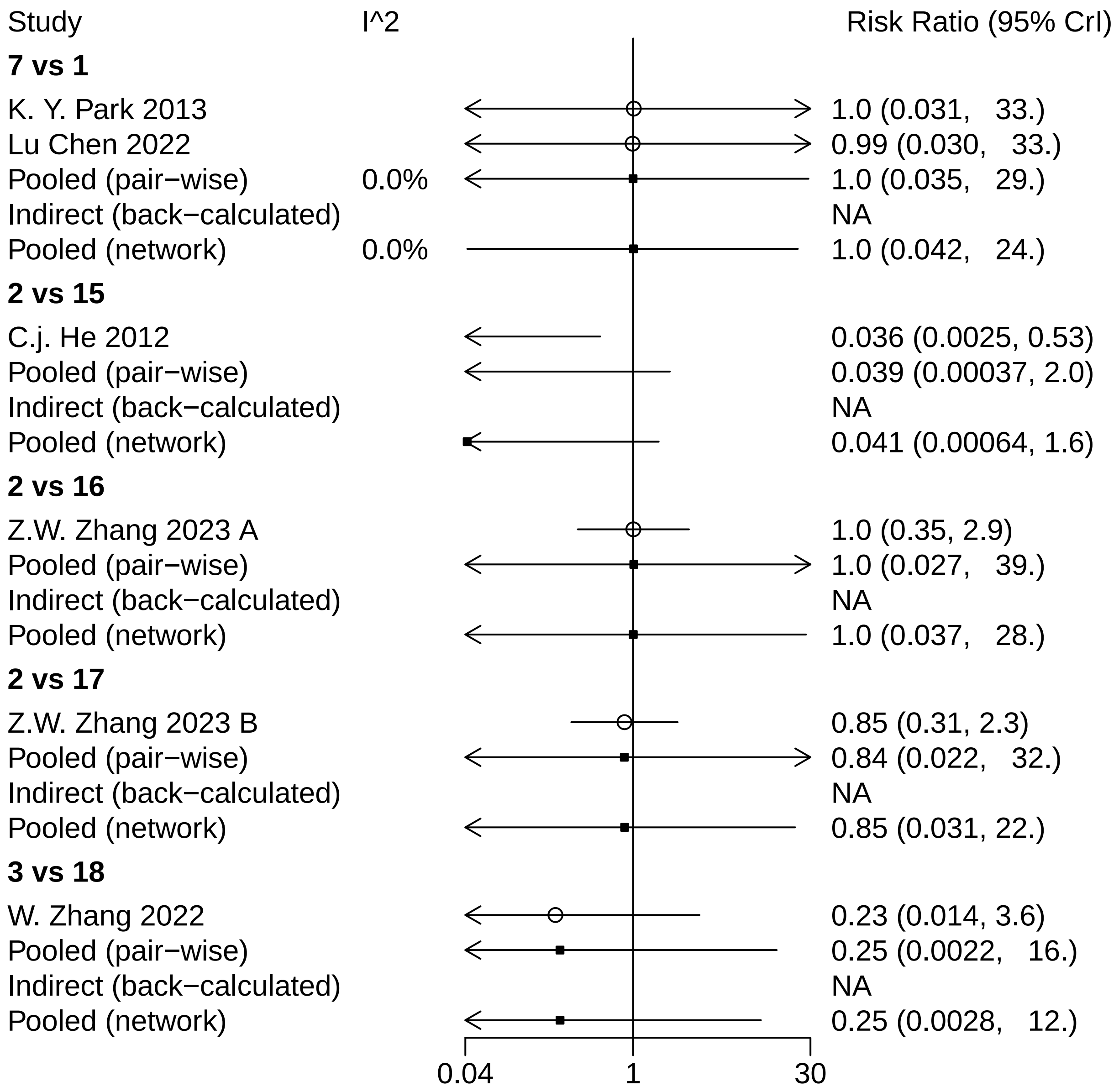
Study
I^2
Risk Ratio (95% CrI)
7 vs 1
K. Y. Park 2013
1.0 (0.031, 33.)
Lu Chen 2022
0.99 (0.030, 33.)
Pooled (pair−wise)
0.0%
1.0 (0.035, 29.)
Indirect (back−calculated)
NA
Pooled (network)
0.0%
1.0 (0.042, 24.)
2 vs 15
C.j. He 2012
0.036 (0.0025, 0.53)
Pooled (pair−wise)
0.039 (0.00037, 2.0)
Indirect (back−calculated)
NA
Pooled (network)
0.041 (0.00064, 1.6)
2 vs 16
Z.W. Zhang 2023 A
1.0 (0.35, 2.9)
Pooled (pair−wise)
1.0 (0.027, 39.)
Indirect (back−calculated)
NA
Pooled (network)
1.0 (0.037, 28.)
2 vs 17
Z.W. Zhang 2023 B
0.85 (0.31, 2.3)
Pooled (pair−wise)
0.84 (0.022, 32.)
Indirect (back−calculated)
NA
Pooled (network)
0.85 (0.031, 22.)
3 vs 18
W. Zhang 2022
0.23 (0.014, 3.6)
Pooled (pair−wise)
0.25 (0.0022, 16.)
Indirect (back−calculated)
NA
Pooled (network)
0.25 (0.0028, 12.)
0.04
1
30

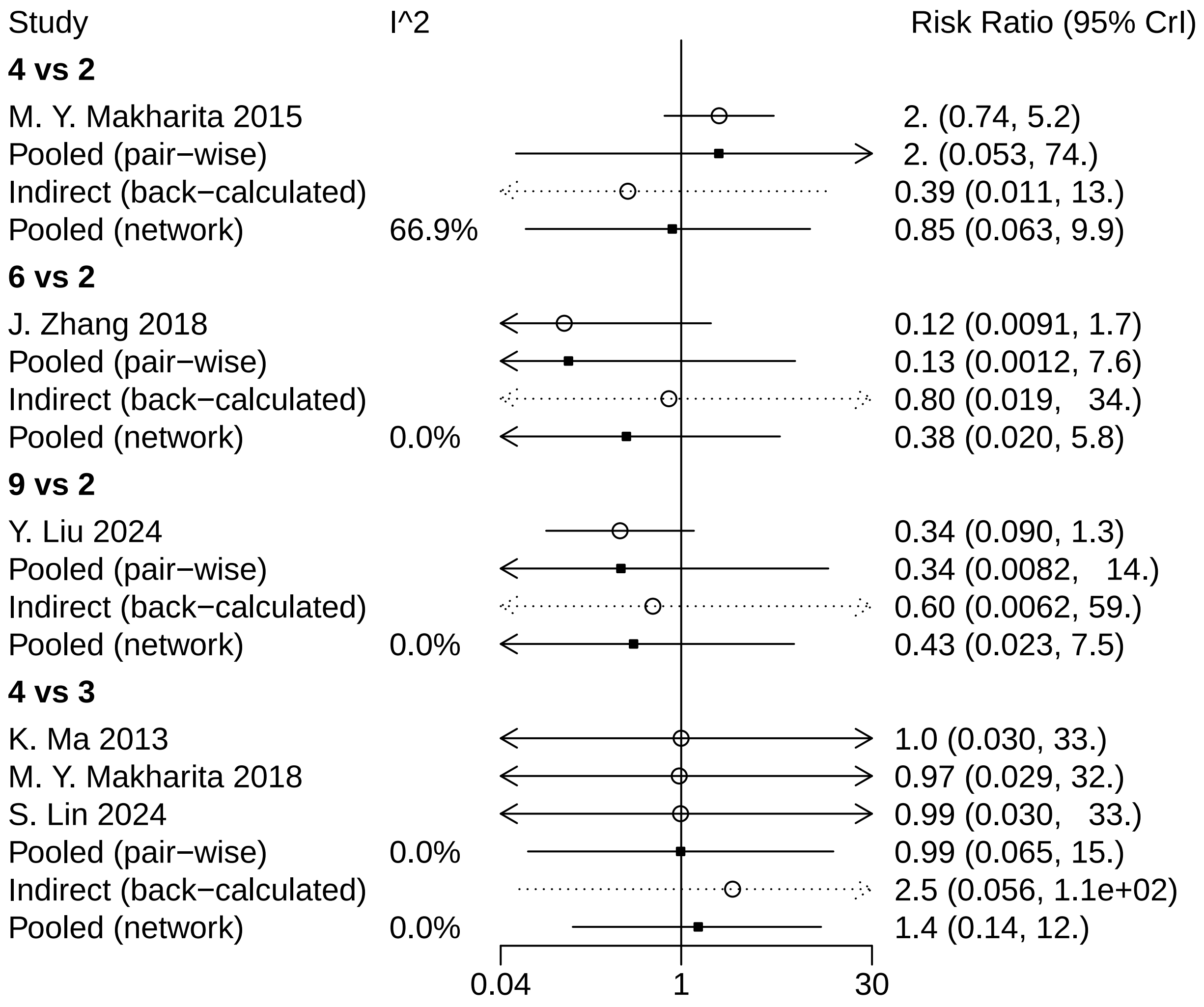
Study
I^2
Risk Ratio (95% CrI)
4 vs 2
M. Y. Makharita 2015
2. (0.74, 5.2)
Pooled (pair−wise)
2. (0.053, 74.)
Indirect (back−calculated)
0.39 (0.011, 13.)
Pooled (network)
66.9%
0.85 (0.063, 9.9)
6 vs 2
J. Zhang 2018
0.12 (0.0091, 1.7)
Pooled (pair−wise)
0.13 (0.0012, 7.6)
Indirect (back−calculated)
0.80 (0.019, 34.)
Pooled (network)
0.0%
0.38 (0.020, 5.8)
9 vs 2
Y. Liu 2024
0.34 (0.090, 1.3)
Pooled (pair−wise)
0.34 (0.0082, 14.)
Indirect (back−calculated)
0.60 (0.0062, 59.)
Pooled (network)
0.0%
0.43 (0.023, 7.5)
4 vs 3
K. Ma 2013
1.0 (0.030, 33.)
M. Y. Makharita 2018
0.97 (0.029, 32.)
S. Lin 2024
0.99 (0.030, 33.)
Pooled (pair−wise)
0.0%
0.99 (0.065, 15.)
Indirect (back−calculated)
2.5 (0.056, 1.1e+02)
Pooled (network)
0.0%
1.4 (0.14, 12.)
0.04
1
30

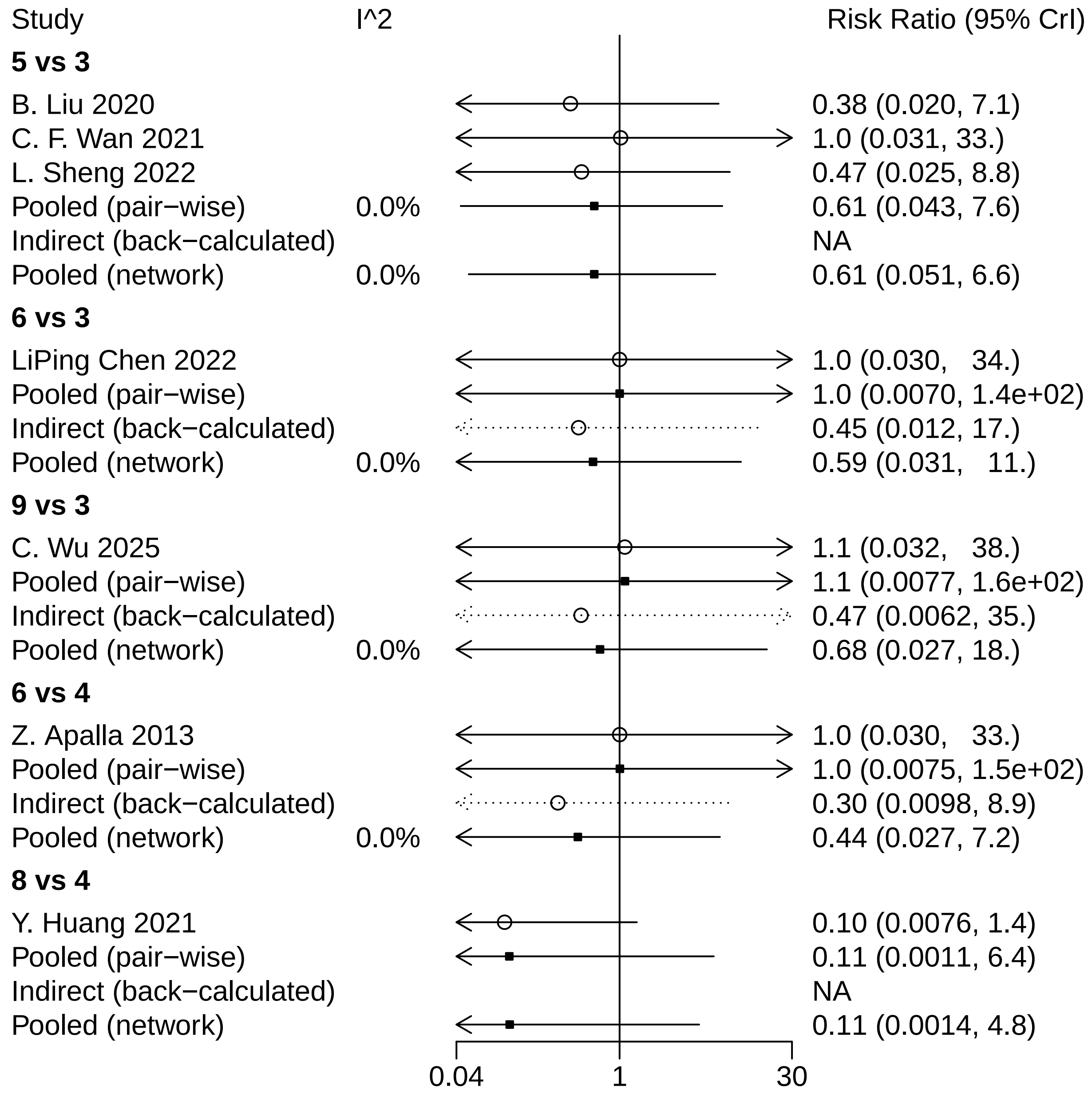
Study
I^2
Risk Ratio (95% CrI)
5 vs 3
B. Liu 2020
0.38 (0.020, 7.1)
C. F. Wan 2021
1.0 (0.031, 33.)
L. Sheng 2022
0.47 (0.025, 8.8)
Pooled (pair−wise)
0.0%
0.61 (0.043, 7.6)
Indirect (back−calculated)
NA
Pooled (network)
0.0%
0.61 (0.051, 6.6)
6 vs 3
LiPing Chen 2022
1.0 (0.030, 34.)
Pooled (pair−wise)
1.0 (0.0070, 1.4e+02)
Indirect (back−calculated)
0.45 (0.012, 17.)
Pooled (network)
0.0%
0.59 (0.031, 11.)
9 vs 3
C. Wu 2025
1.1 (0.032, 38.)
Pooled (pair−wise)
1.1 (0.0077, 1.6e+02)
Indirect (back−calculated)
0.47 (0.0062, 35.)
Pooled (network)
0.0%
0.68 (0.027, 18.)
6 vs 4
Z. Apalla 2013
1.0 (0.030, 33.)
Pooled (pair−wise)
1.0 (0.0075, 1.5e+02)
Indirect (back−calculated)
0.30 (0.0098, 8.9)
Pooled (network)
0.0%
0.44 (0.027, 7.2)
8 vs 4
Y. Huang 2021
0.10 (0.0076, 1.4)
Pooled (pair−wise)
0.11 (0.0011, 6.4)
Indirect (back−calculated)
NA
Pooled (network)
0.11 (0.0014, 4.8)
0.04
1
30

Supplementary Figure 3 Forest plots with heterogeneity assessment for the adverse events outcome.
Note: This forest plot presents the evidence synthesis results for each direct comparison regarding the adverse events outcome, based on a Bayesian random-effects model. It displays the direct pairwise meta-analysis results, indirect effect estimates, and the comprehensive network meta-analysis estimates for each comparison. All effect estimates are expressed as risk ratio (RR) with 95% credible intervals (95% CrI). The I^2 statistic was used to assess local heterogeneity, with higher values indicating greater heterogeneity. The correspondence between intervention codes/abbreviations and their full names is provided in Table S5.
