# Supplementary material for: Comparison of efficacy and safety of non-oral therapeutic interventions for zoster-associated pain: a systematic review and network meta-analysis
Source: Front Neurol. 2026 Jan 27;17:1711536. doi: 10.3389/fneur.2026.1711536 (PMC12886049; doi:10.3389/fneur.2026.1711536)
Supplement: Supplementary file 1 [file Data_Sheet_1.zip › Supplementary_Material_Complete/Data Sheet 4.pdf]

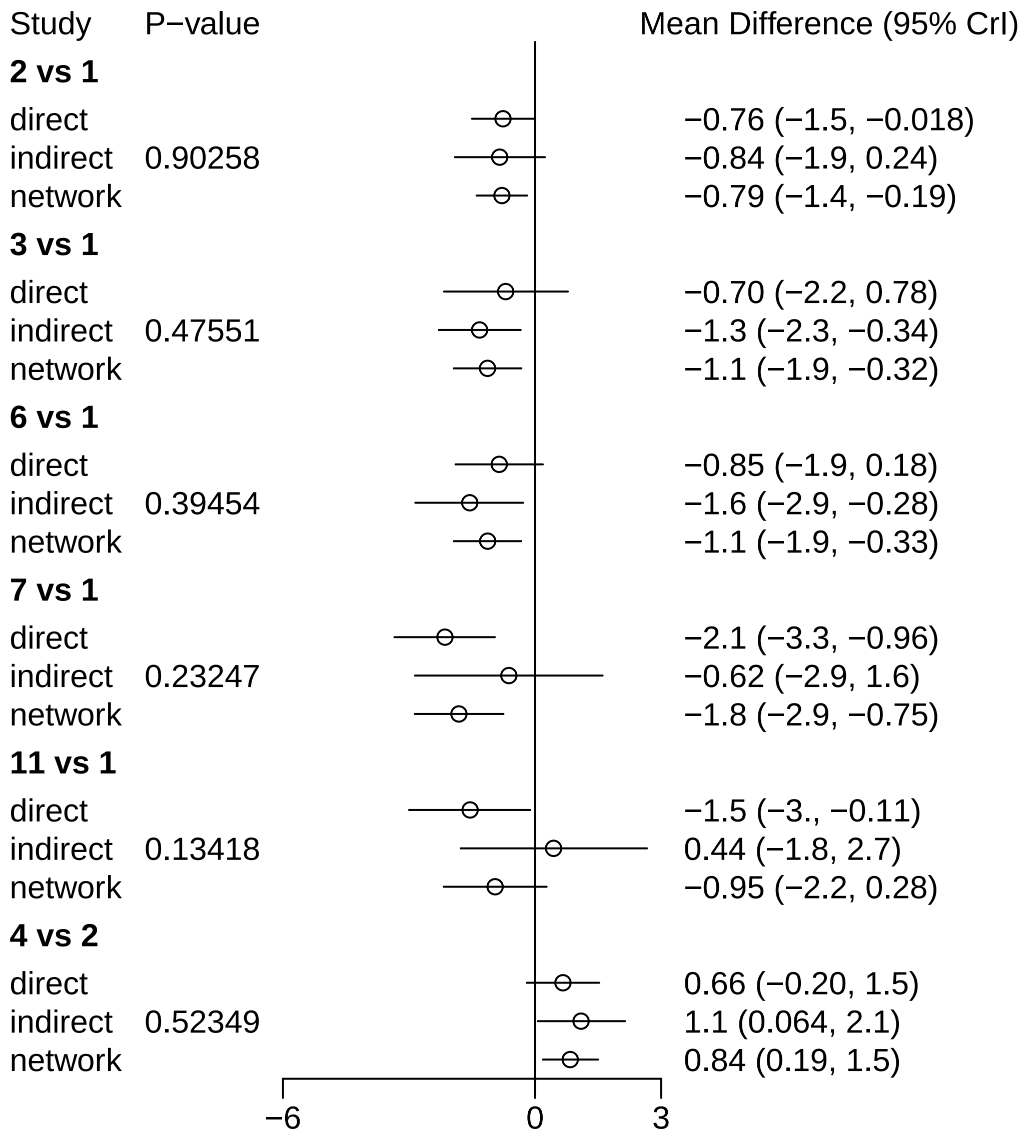
Study
P−value
Mean Difference (95% CrI)
2 vs 1
direct −0.76 (−1.5, −0.018)
indirect 0.90258 −0.84 (−1.9, 0.24)
network −0.79 (−1.4, −0.19)
3 vs 1
direct −0.70 (−2.2, 0.78)
indirect 0.47551 −1.3 (−2.3, −0.34)
network −1.1 (−1.9, −0.32)
6 vs 1
direct −0.85 (−1.9, 0.18)
indirect 0.39454 −1.6 (−2.9, −0.28)
network −1.1 (−1.9, −0.33)
7 vs 1
direct −2.1 (−3.3, −0.96)
indirect 0.23247 −0.62 (−2.9, 1.6)
network −1.8 (−2.9, −0.75)
11 vs 1
direct −1.5 (−3., −0.11)
indirect 0.13418 0.44 (−1.8, 2.7)
network −0.95 (−2.2, 0.28)
4 vs 2
direct 0.66 (−0.20, 1.5)
indirect 0.52349 1.1 (0.064, 2.1)
network 0.84 (0.19, 1.5)
−6
0
3

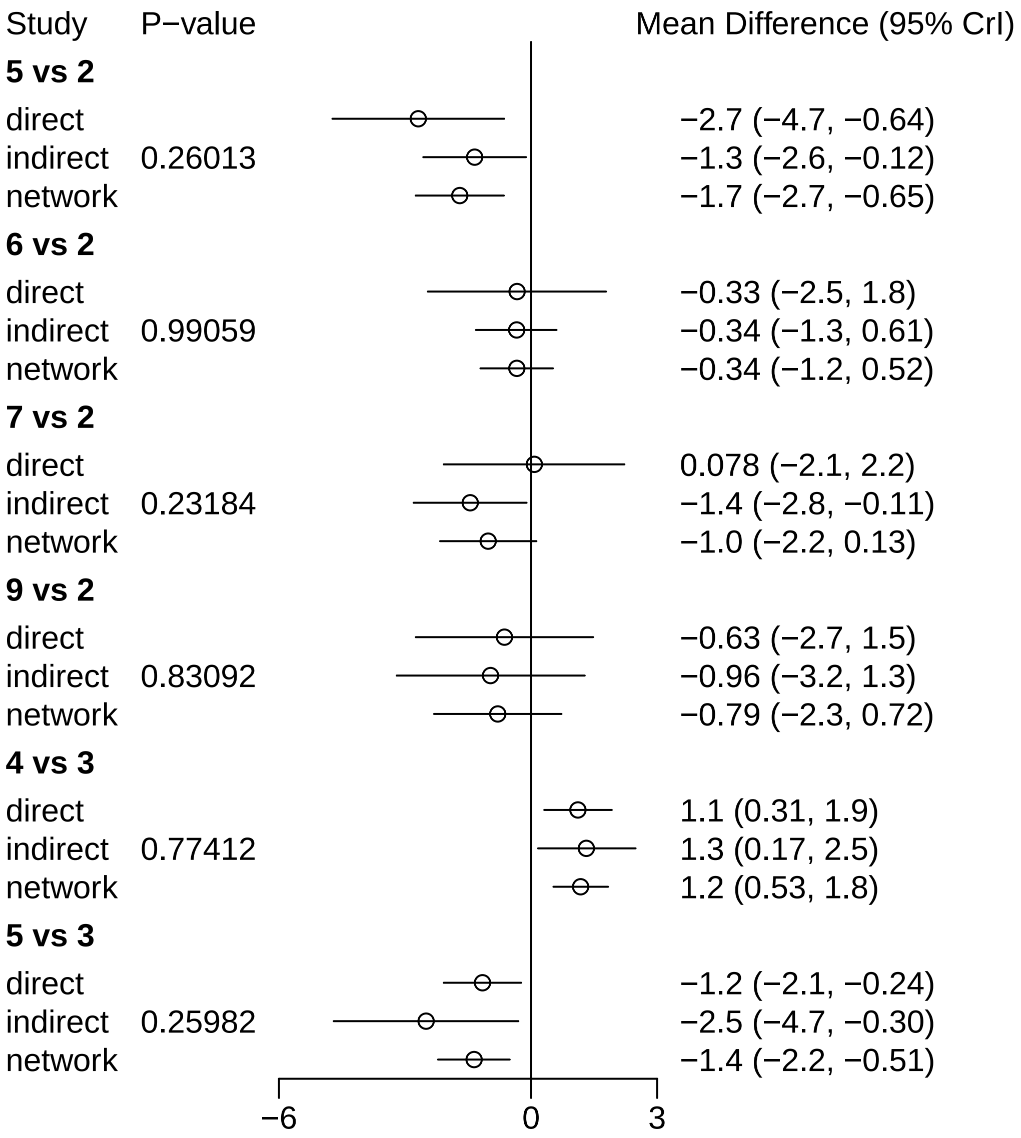
Study
P−value
Mean Difference (95% CrI)
5 vs 2
direct −2.7 (−4.7, −0.64)
indirect 0.26013 −1.3 (−2.6, −0.12)
network −1.7 (−2.7, −0.65)
6 vs 2
direct −0.33 (−2.5, 1.8)
indirect 0.99059 −0.34 (−1.3, 0.61)
network −0.34 (−1.2, 0.52)
7 vs 2
direct 0.078 (−2.1, 2.2)
indirect 0.23184 −1.4 (−2.8, −0.11)
network −1.0 (−2.2, 0.13)
9 vs 2
direct −0.63 (−2.7, 1.5)
indirect 0.83092 −0.96 (−3.2, 1.3)
network −0.79 (−2.3, 0.72)
4 vs 3
direct 1.1 (0.31, 1.9)
indirect 0.77412 1.3 (0.17, 2.5)
network 1.2 (0.53, 1.8)
5 vs 3
direct −1.2 (−2.1, −0.24)
indirect 0.25982 −2.5 (−4.7, −0.30)
network −1.4 (−2.2, −0.51)
−6
0
3

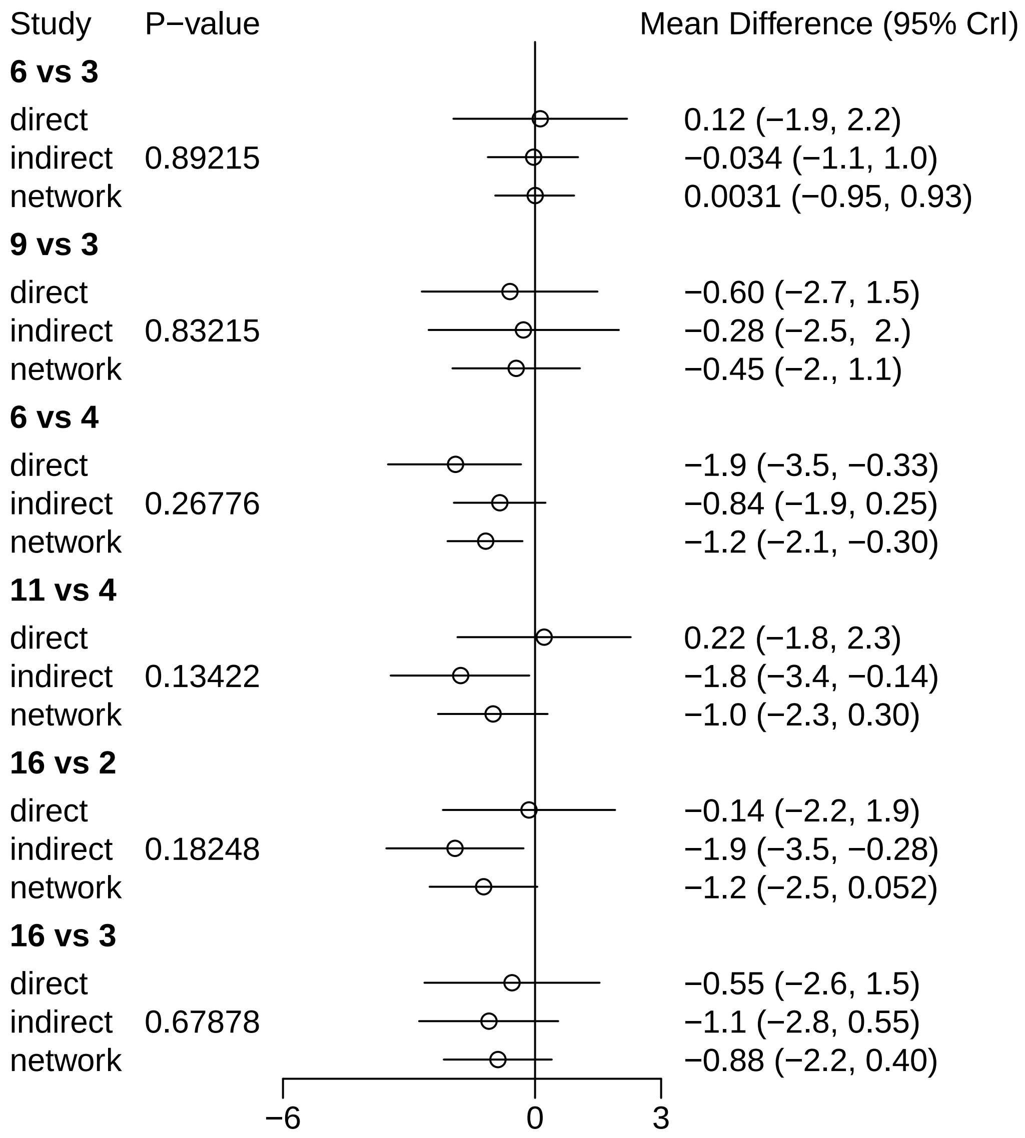
Study
P−value
Mean Difference (95% CrI)
6 vs 3
direct
0.12 (−1.9, 2.2)
indirect
0.89215
−0.034 (−1.1, 1.0)
network
0.0031 (−0.95, 0.93)
9 vs 3
direct
−0.60 (−2.7, 1.5)
indirect
0.83215
−0.28 (−2.5, 2.)
network
−0.45 (−2., 1.1)
6 vs 4
direct
−1.9 (−3.5, −0.33)
indirect
0.26776
−0.84 (−1.9, 0.25)
network
−1.2 (−2.1, −0.30)
11 vs 4
direct
0.22 (−1.8, 2.3)
indirect
0.13422
−1.8 (−3.4, −0.14)
network
−1.0 (−2.3, 0.30)
16 vs 2
direct
−0.14 (−2.2, 1.9)
indirect
0.18248
−1.9 (−3.5, −0.28)
network
−1.2 (−2.5, 0.052)
16 vs 3
direct
−0.55 (−2.6, 1.5)
indirect
0.67878
−1.1 (−2.8, 0.55)
network
−0.88 (−2.2, 0.40)
−6
0
3

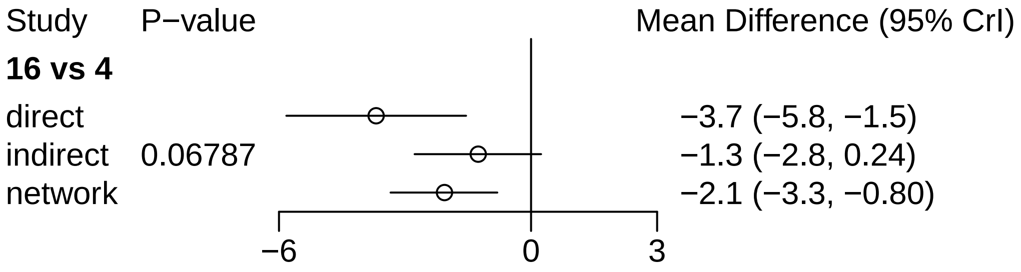


**Supplementary Figure 4** Forest plots of local inconsistency for the pain relief outcome in Bayesian network meta-analysis.

Note: This figure presents the direct, indirect, and network meta-analysis estimates for each comparison in the pain relief outcome, expressed as mean difference (MD) with 95% credible intervals (95% CrI). A *P*-value < 0.05 indicates significant local inconsistency between direct and indirect evidence. The correspondence between intervention codes/abbreviations and their full names is provided in Table S5.
