# Supplementary material for: Comparison of efficacy and safety of non-oral therapeutic interventions for zoster-associated pain: a systematic review and network meta-analysis
Source: Front Neurol. 2026 Jan 27;17:1711536. doi: 10.3389/fneur.2026.1711536 (PMC12886049; doi:10.3389/fneur.2026.1711536)
Supplement: Supplementary file 1 [file Data_Sheet_1.zip › Supplementary_Material_Complete/Data Sheet 5.pdf]

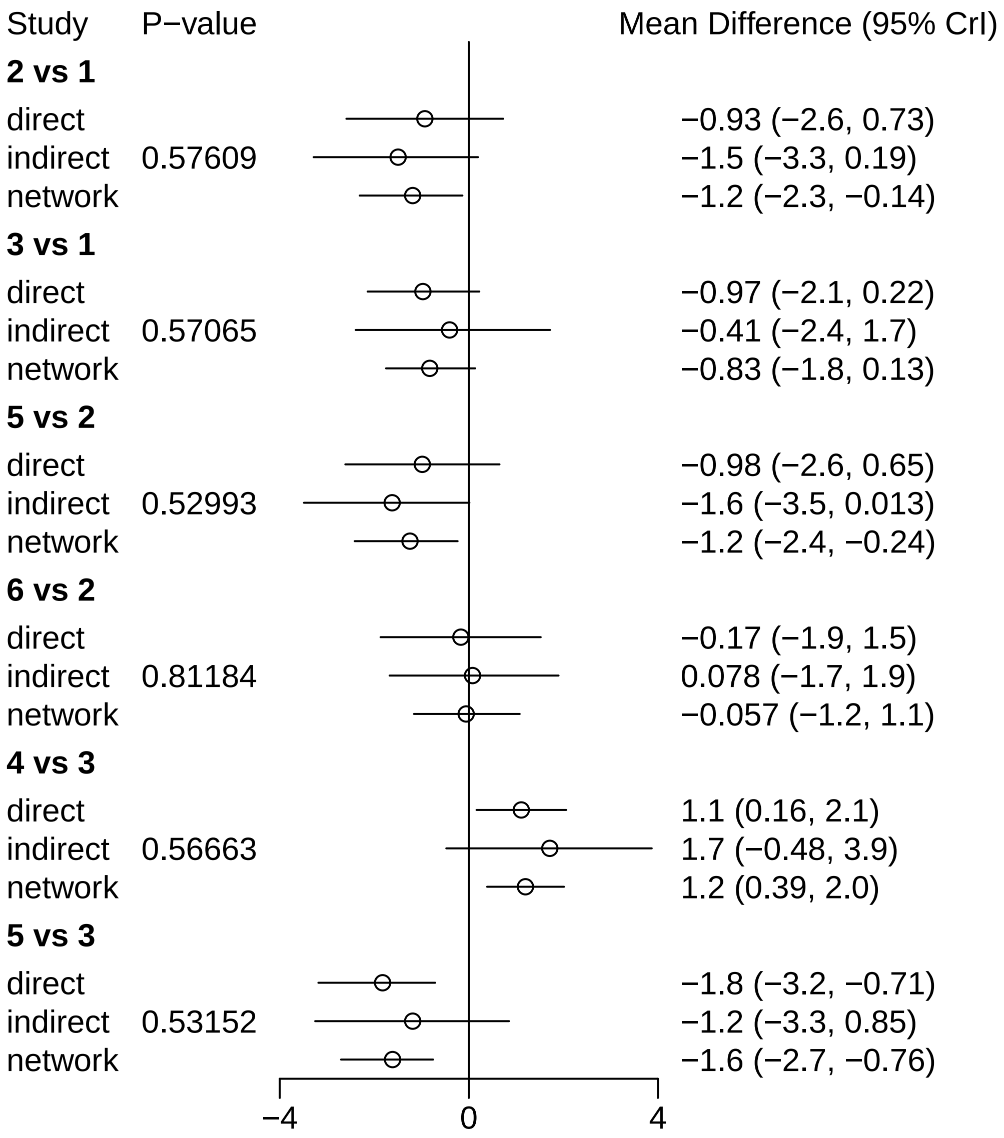
Study
P−value
Mean Difference (95% CrI)
2 vs 1
direct
−0.93 (−2.6, 0.73)
indirect
0.57609
−1.5 (−3.3, 0.19)
network
−1.2 (−2.3, −0.14)
3 vs 1
direct
−0.97 (−2.1, 0.22)
indirect
0.57065
−0.41 (−2.4, 1.7)
network
−0.83 (−1.8, 0.13)
5 vs 2
direct
−0.98 (−2.6, 0.65)
indirect
0.52993
−1.6 (−3.5, 0.013)
network
−1.2 (−2.4, −0.24)
6 vs 2
direct
−0.17 (−1.9, 1.5)
indirect
0.81184
0.078 (−1.7, 1.9)
network
−0.057 (−1.2, 1.1)
4 vs 3
direct
1.1 (0.16, 2.1)
indirect
0.56663
1.7 (−0.48, 3.9)
network
1.2 (0.39, 2.0)
5 vs 3
direct
−1.8 (−3.2, −0.71)
indirect
0.53152
−1.2 (−3.3, 0.85)
network
−1.6 (−2.7, −0.76)
−4
0
4

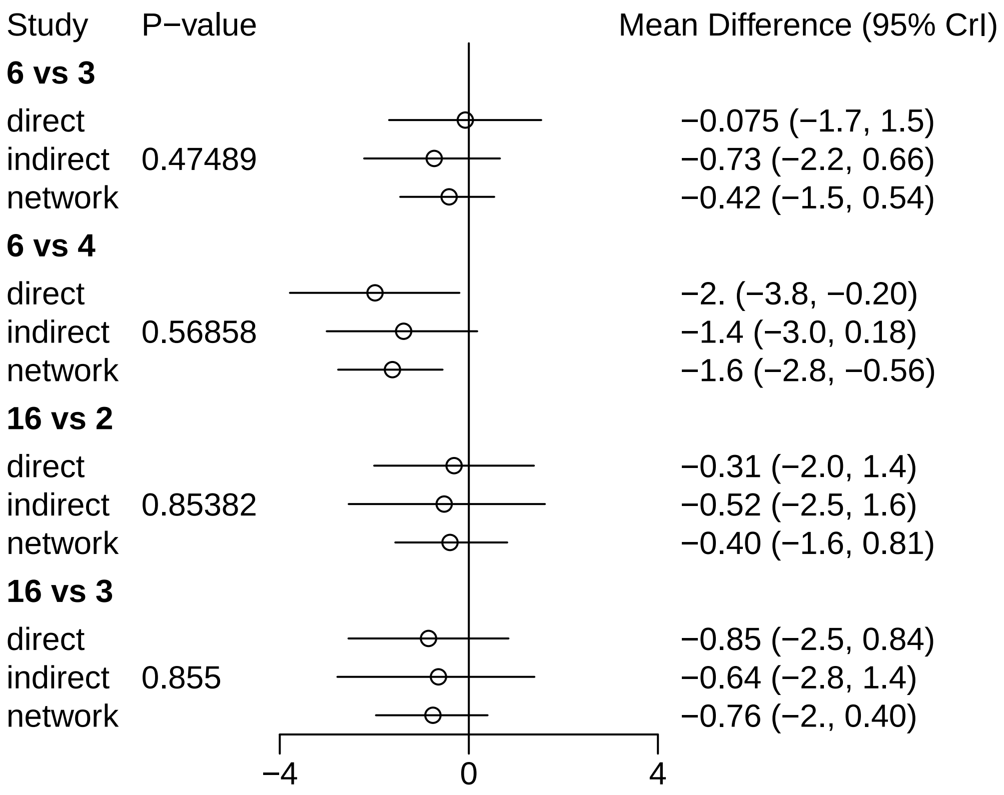

Supplementary Figure 5 Forest plots of local inconsistency for the sleep quality outcome in Bayesian network meta-analysis.

Note: This figure presents the direct, indirect, and network meta-analysis estimates for each comparison in the sleep quality outcome, expressed as mean difference (MD) with 95% credible intervals (95% CrI). A *P*-value < 0.05 indicates significant local inconsistency between direct and indirect evidence. The correspondence between intervention codes/abbreviations and their full names is provided in Table S5.
