# Supplementary material for: Comparison of efficacy and safety of non-oral therapeutic interventions for zoster-associated pain: a systematic review and network meta-analysis
Source: Front Neurol. 2026 Jan 27;17:1711536. doi: 10.3389/fneur.2026.1711536 (PMC12886049; doi:10.3389/fneur.2026.1711536)
Supplement: Supplementary file 1 [file Data_Sheet_1.zip › Supplementary_Material_Complete/Data Sheet 6.pdf]

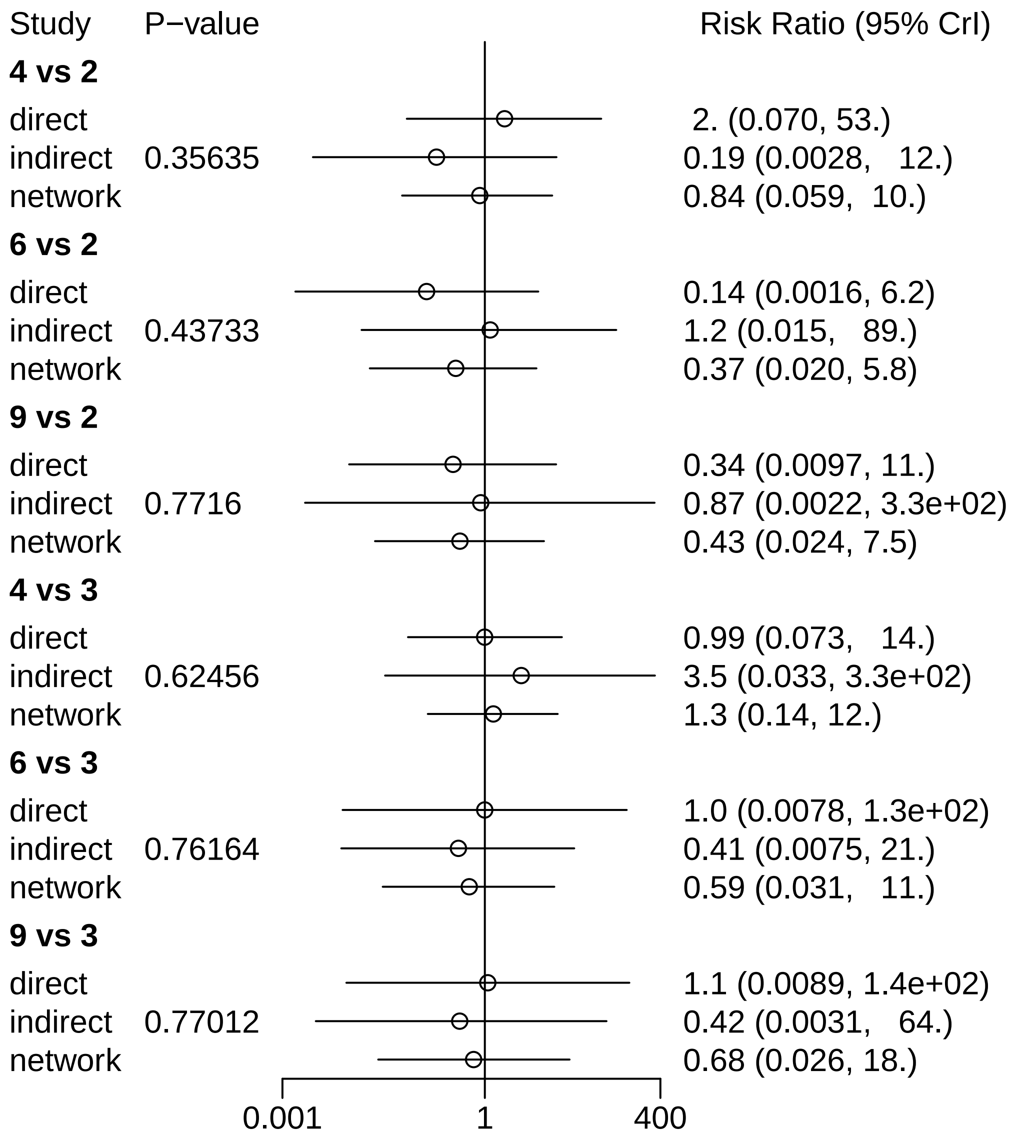
Study
P−value
Risk Ratio (95% CrI)
4 vs 2
direct
2. (0.070, 53.)
indirect
0.35635
0.19 (0.0028, 12.)
network
0.84 (0.059, 10.)
6 vs 2
direct
0.14 (0.0016, 6.2)
indirect
0.43733
1.2 (0.015, 89.)
network
0.37 (0.020, 5.8)
9 vs 2
direct
0.34 (0.0097, 11.)
indirect
0.7716
0.87 (0.0022, 3.3e+02)
network
0.43 (0.024, 7.5)
4 vs 3
direct
0.99 (0.073, 14.)
indirect
0.62456
3.5 (0.033, 3.3e+02)
network
1.3 (0.14, 12.)
6 vs 3
direct
1.0 (0.0078, 1.3e+02)
indirect
0.76164
0.41 (0.0075, 21.)
network
0.59 (0.031, 11.)
9 vs 3
direct
1.1 (0.0089, 1.4e+02)
indirect
0.77012
0.42 (0.0031, 64.)
network
0.68 (0.026, 18.)
0.001
1
400

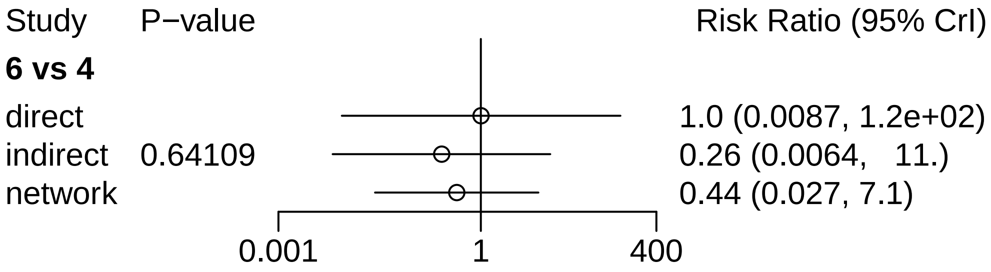

Supplementary Figure 6 Forest plots of local inconsistency for the adverse events outcome in Bayesian network meta-analysis.

Note: This figure presents the direct, indirect, and network meta-analysis estimates for each comparison in the adverse events outcome, expressed as risk ratio (RR) with 95% credible intervals (95% CrI). A *P*-value < 0.05 indicates significant local inconsistency between direct and indirect evidence. The correspondence between intervention codes/abbreviations and their full names is provided in Table S5.
