# Supplementary material for: Comparison of efficacy and safety of non-oral therapeutic interventions for zoster-associated pain: a systematic review and network meta-analysis
Source: Front Neurol. 2026 Jan 27;17:1711536. doi: 10.3389/fneur.2026.1711536 (PMC12886049; doi:10.3389/fneur.2026.1711536)
Supplement: Supplementary file 1 [file Data_Sheet_1.zip › Supplementary_Material_Complete/Data Sheet 7.pdf]

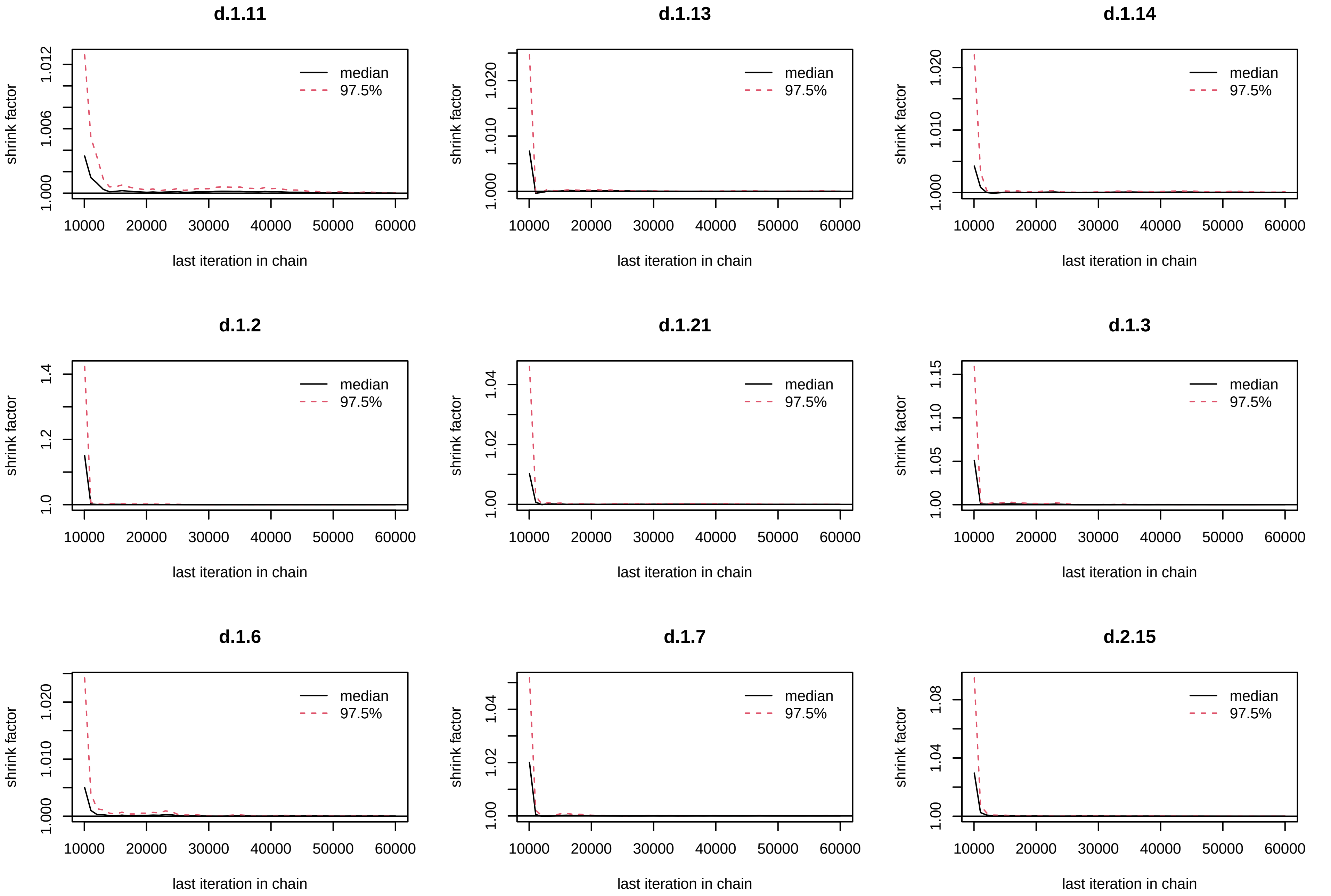
d.1.11
shrink factor
1.000
1.006
1.012
median
97.5%
10000
20000
30000
40000
50000
60000
last iteration in chain
d.1.13
shrink factor
1.000
1.010
1.020
median
97.5%
10000
20000
30000
40000
50000
60000
last iteration in chain
d.1.14
shrink factor
1.000
1.010
1.020
median
97.5%
10000
20000
30000
40000
50000
60000
last iteration in chain
d.1.2
shrink factor
1.0
1.2
1.4
median
97.5%
10000
20000
30000
40000
50000
60000
last iteration in chain
d.1.21
shrink factor
1.00
1.02
1.04
median
97.5%
10000
20000
30000
40000
50000
60000
last iteration in chain
d.1.3
shrink factor
1.00
1.05
1.10
1.15
median
97.5%
10000
20000
30000
40000
50000
60000
last iteration in chain
d.1.6
shrink factor
1.000
1.010
1.020
median
97.5%
10000
20000
30000
40000
50000
60000
last iteration in chain
d.1.7
shrink factor
1.00
1.02
1.04
median
97.5%
10000
20000
30000
40000
50000
60000
last iteration in chain
d.2.15
shrink factor
1.00
1.04
1.08
median
97.5%
10000
20000
30000
40000
50000
60000
last iteration in chain

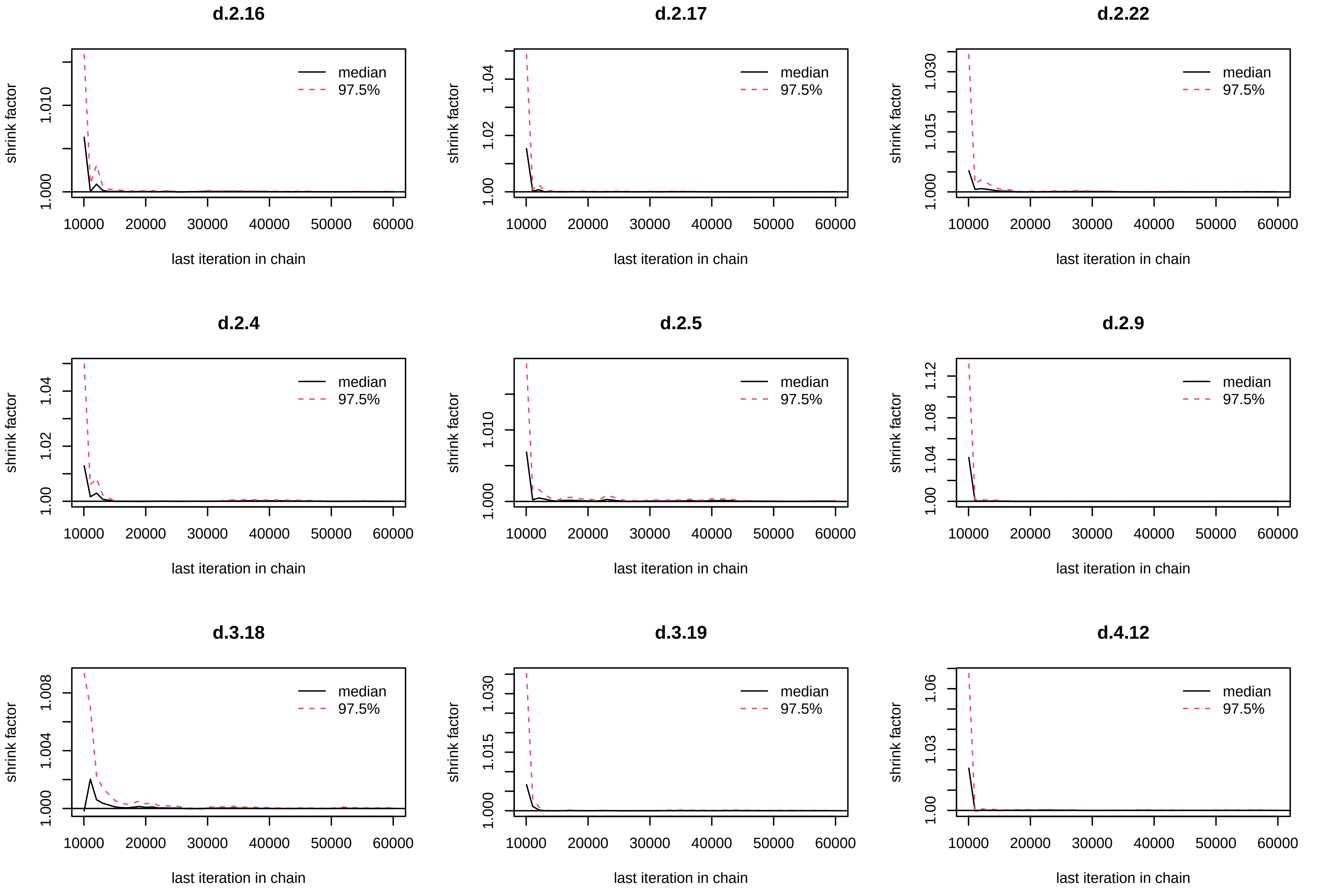
d.2.16
shrink factor
1.000
1.010
10000 20000 30000 40000 50000 60000
last iteration in chain
median
97.5%
d.2.17
shrink factor
1.00
1.02
1.04
10000 20000 30000 40000 50000 60000
last iteration in chain
median
97.5%
d.2.22
shrink factor
1.000
1.015
1.030
10000 20000 30000 40000 50000 60000
last iteration in chain
median
97.5%
d.2.4
shrink factor
1.00
1.02
1.04
10000 20000 30000 40000 50000 60000
last iteration in chain
median
97.5%
d.2.5
shrink factor
1.000
1.010
10000 20000 30000 40000 50000 60000
last iteration in chain
median
97.5%
d.2.9
shrink factor
1.00
1.04
1.08
1.12
10000 20000 30000 40000 50000 60000
last iteration in chain
median
97.5%
d.3.18
shrink factor
1.000
1.004
1.008
10000 20000 30000 40000 50000 60000
last iteration in chain
median
97.5%
d.3.19
shrink factor
1.000
1.015
1.030
10000 20000 30000 40000 50000 60000
last iteration in chain
median
97.5%
d.4.12
shrink factor
1.00
1.03
1.06
10000 20000 30000 40000 50000 60000
last iteration in chain
median
97.5%

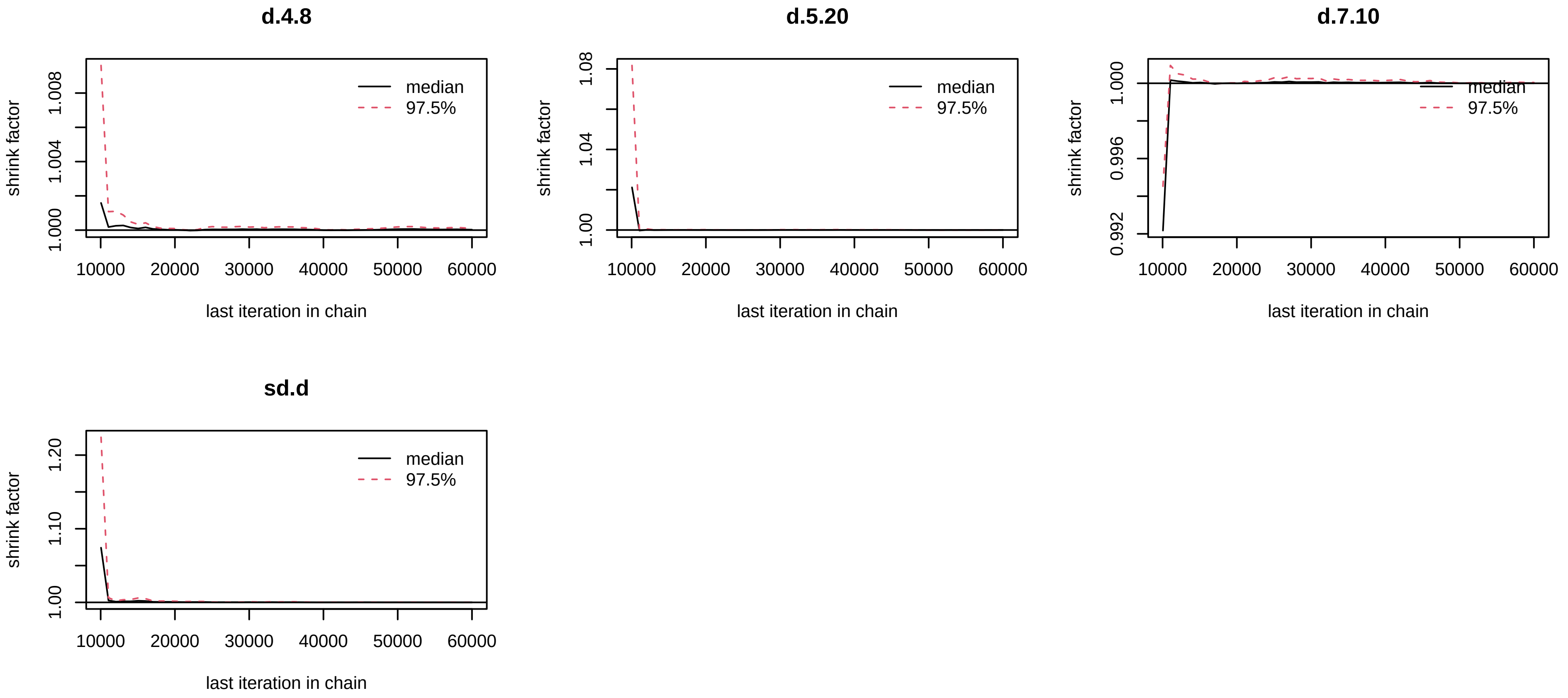

Supplementary Figure 7 Potential scale reduction factor (PSRF) plots for the pain relief outcome.
Note: This figure presents convergence diagnostic results using the potential scale reduction factor (PSRF) for key parameters in the pain relief network. A PSRF value approaching 1 (ideally < 1.05) indicates successful convergence of the Markov chains. The correspondence between intervention codes/abbreviations and their full names is provided in Table S5.
