# Supplementary material for: Comparison of efficacy and safety of non-oral therapeutic interventions for zoster-associated pain: a systematic review and network meta-analysis
Source: Front Neurol. 2026 Jan 27;17:1711536. doi: 10.3389/fneur.2026.1711536 (PMC12886049; doi:10.3389/fneur.2026.1711536)
Supplement: Supplementary file 1 [file Data_Sheet_1.zip › Supplementary_Material_Complete/Data Sheet 8.pdf]

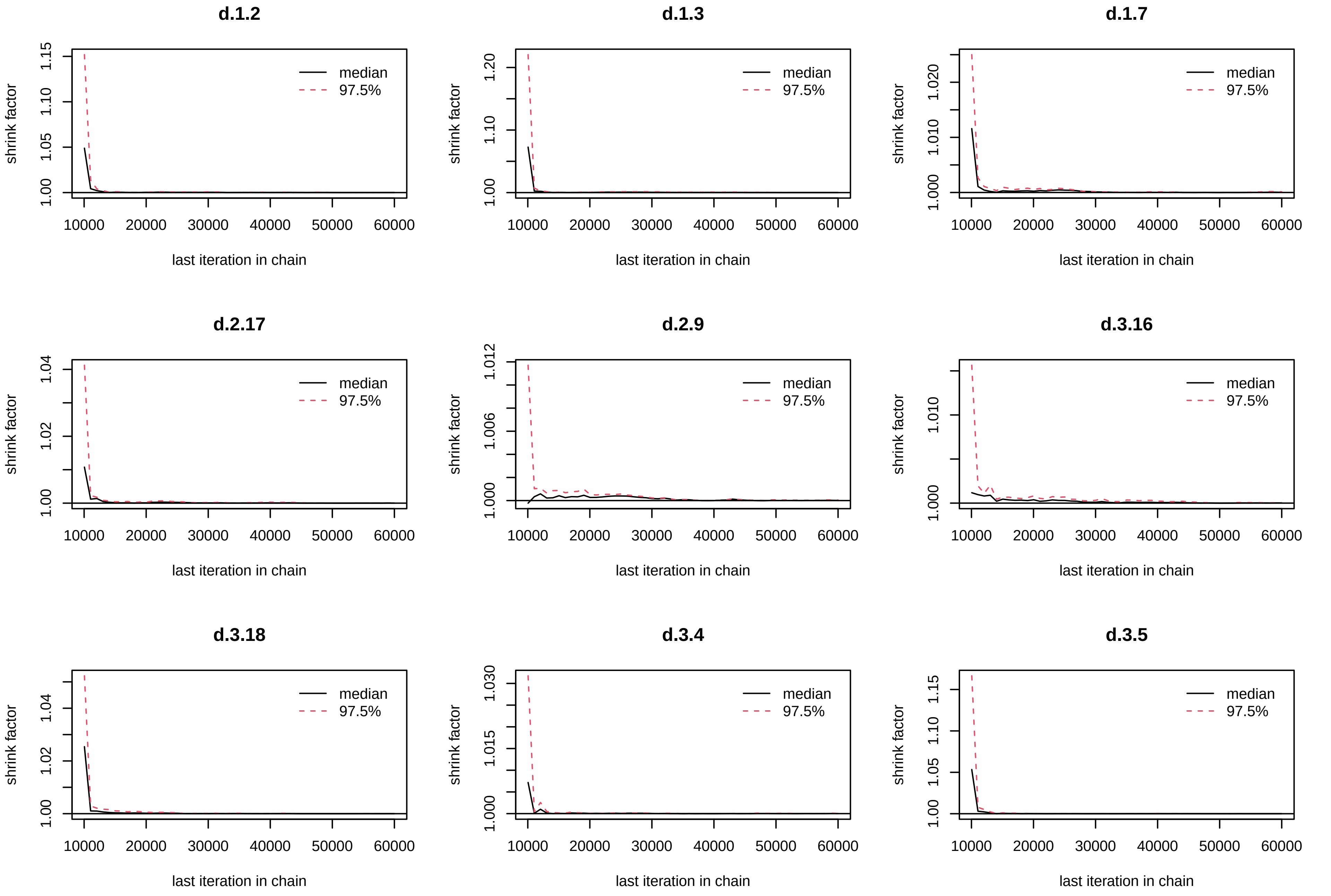
d.1.2
d.1.3
d.1.7
d.2.17
d.2.9
d.3.16
d.3.18
d.3.4
d.3.5
shrink factor
last iteration in chain
median
97.5%
10000
20000
30000
40000
50000
60000
1.00
1.05
1.10
1.15
1.20
1.000
1.010
1.020
1.02
1.04
1.006
1.012
1.015
1.030

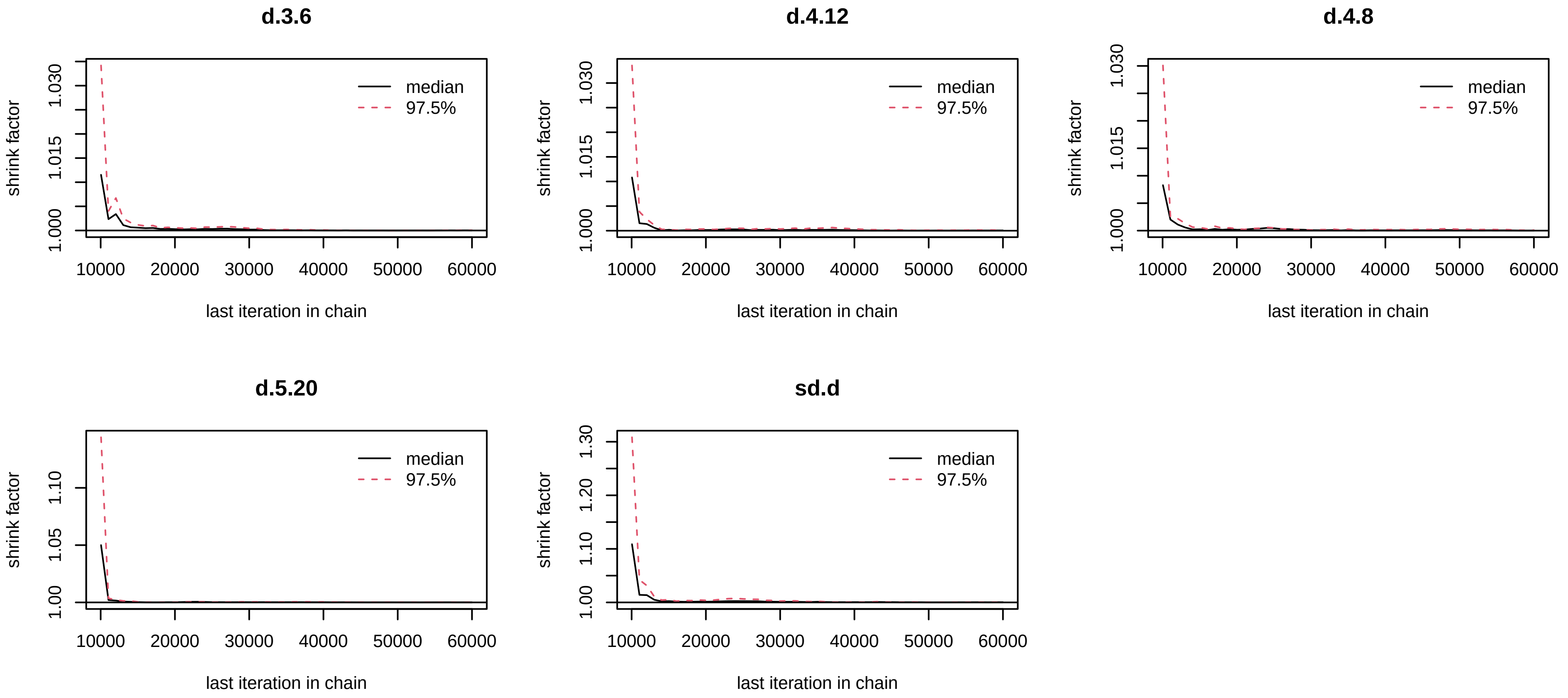

Supplementary Figure 8 Potential scale reduction factor (PSRF) plots for the sleep quality outcome.
Note: This figure presents convergence diagnostic results using the potential scale reduction factor (PSRF) for key parameters in the sleep quality network. A PSRF value approaching 1 (ideally < 1.05) indicates successful convergence of the Markov chains. The correspondence between intervention codes/abbreviations and their full names is provided in Table S5.
