# Supplementary material for: Comparison of efficacy and safety of non-oral therapeutic interventions for zoster-associated pain: a systematic review and network meta-analysis
Source: Front Neurol. 2026 Jan 27;17:1711536. doi: 10.3389/fneur.2026.1711536 (PMC12886049; doi:10.3389/fneur.2026.1711536)
Supplement: Supplementary file 1 [file Data_Sheet_1.zip › Supplementary_Material_Complete/Data Sheet 9.pdf]

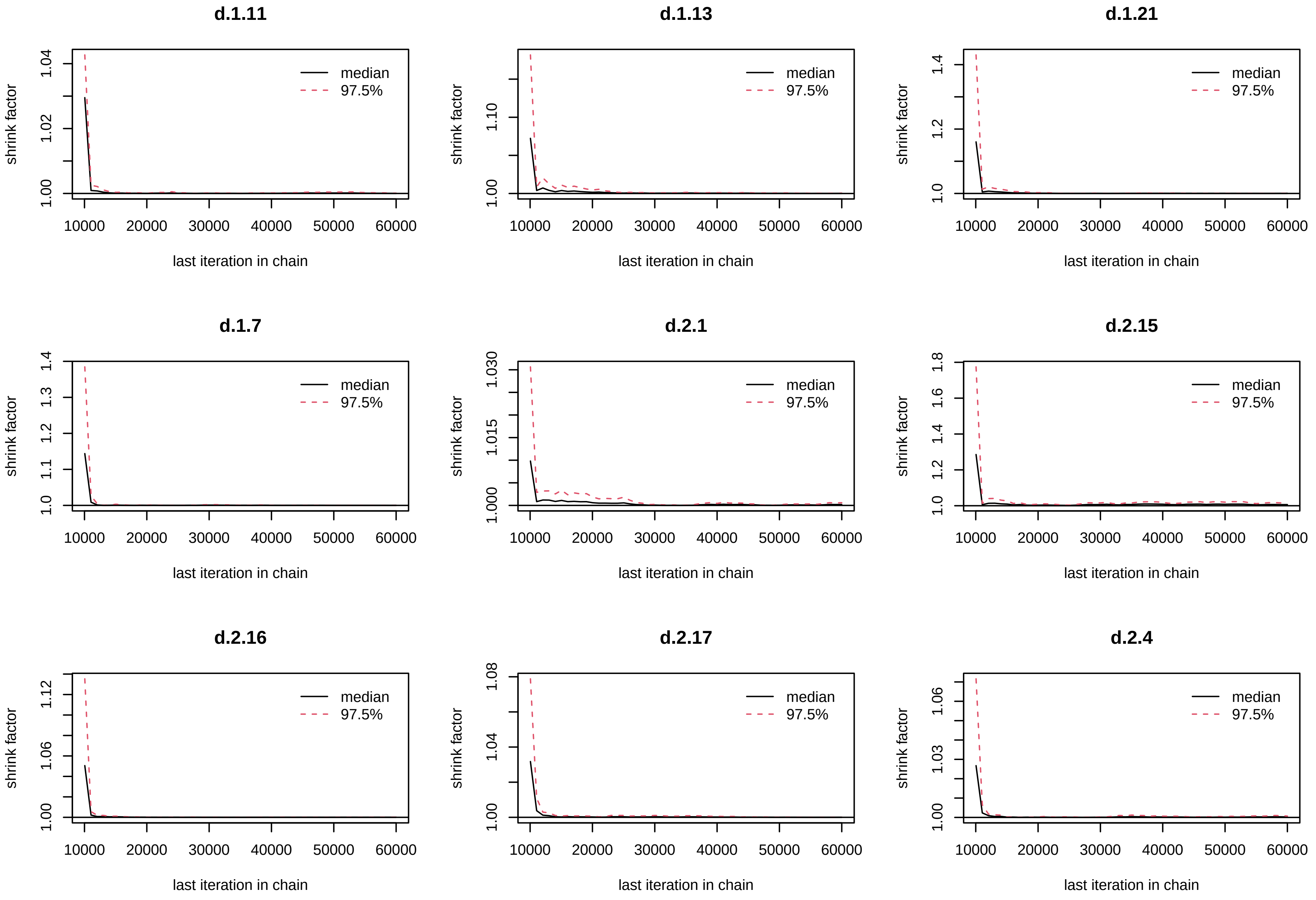

d.1.11
d.1.13
d.1.21
d.1.7
d.2.1
d.2.15
d.2.16
d.2.17
d.2.4
median
97.5%
shrink factor
last iteration in chain
10000
20000
30000
40000
50000
60000
1.00
1.02
1.04
1.10
1.0
1.2
1.4
1.1
1.3
1.000
1.015
1.030
1.6
1.8
1.06
1.12
1.04
1.08
1.03

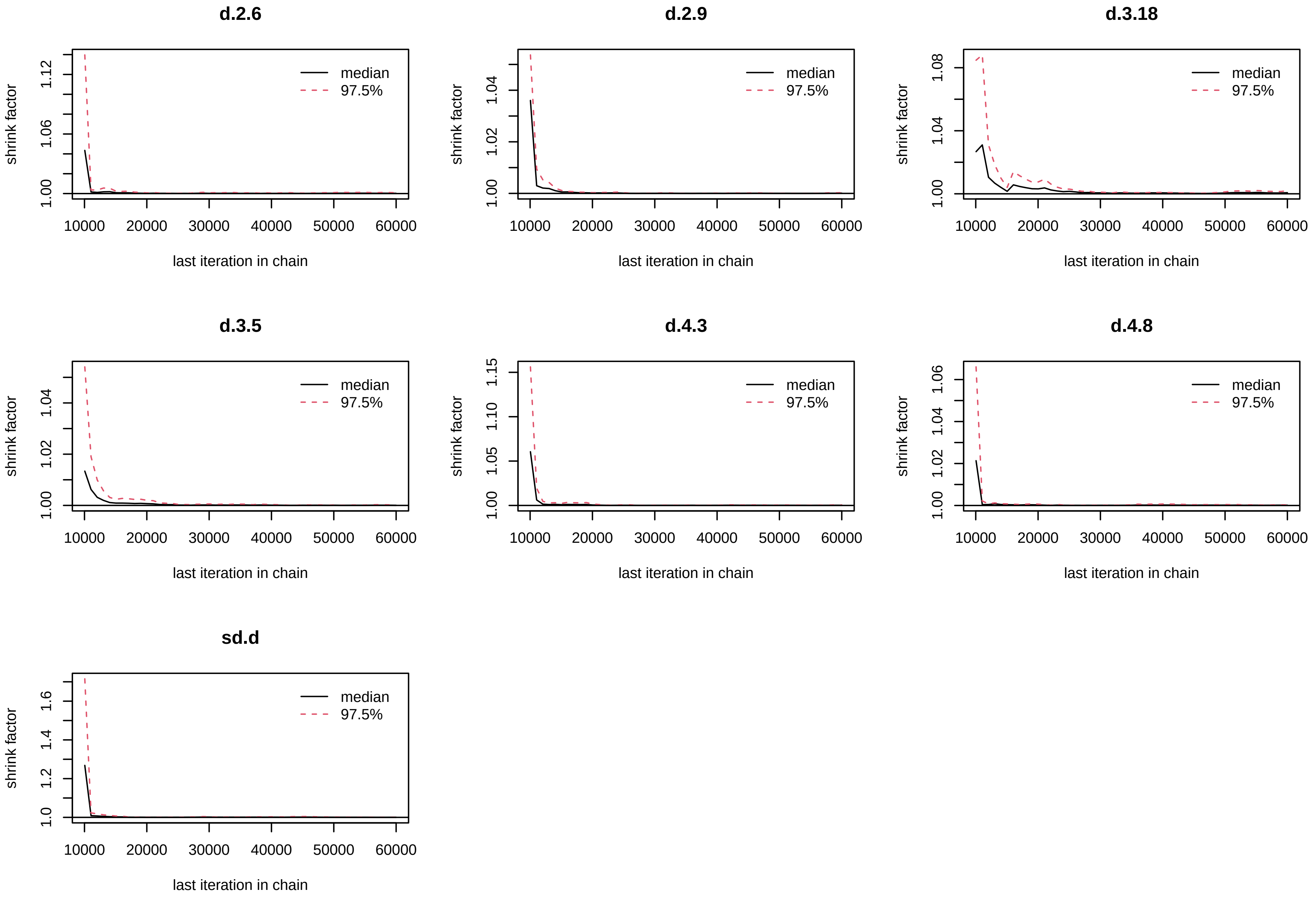
d.2.6
d.2.9
d.3.18
d.3.5
d.4.3
d.4.8
sd.d
median
97.5%
shrink factor
last iteration in chain
10000
20000
30000
40000
50000
60000

Supplementary Figure 9 Potential scale reduction factor (PSRF) plots for the adverse events outcome. Note: This figure presents convergence diagnostic results using the potential scale reduction factor (PSRF) for key parameters in the adverse events network. A PSRF value approaching 1 (ideally < 1.05) indicates successful convergence of the Markov chains. The correspondence between intervention codes/ abbreviations and their full names is provided in Table S5.
