# Supplementary material for: Comparison of efficacy and safety of non-oral therapeutic interventions for zoster-associated pain: a systematic review and network meta-analysis
Source: Front Neurol. 2026 Jan 27;17:1711536. doi: 10.3389/fneur.2026.1711536 (PMC12886049; doi:10.3389/fneur.2026.1711536)
Supplement: Supplementary file 1 [file Data_Sheet_1.zip › Supplementary_Material_Complete/Table 1.docx]

|  | Dbar | pD | DIC | | ΔDIC | I^2^ | |
| --- | --- | --- | --- | --- | --- | --- | --- |
|  |  |  | consistency | ume |  | consistency | ume |
| Pain relief | 65.211 | 59.755 | 124.966 | 124.630 | -0.336 | 5% | 3% |
| Sleep quality | 23.766 | 20.830 | 44.597 | 44.201 | -0.396 | 12% | 6% |
| Adverse events | 55.368 | 53.028 | 108.396 | 112.786 | 4.390 | 0.7% | 4% |

**Table S1** Model fit comparison between consistency and inconsistency models in network meta-analysis

Abbreviations: DIC: Deviance information criterion; ΔDIC: DIC_inconsistency − DIC_consistency; I²: heterogeneity statistic.

Interpretation criteria: ΔDIC: A value > 5 suggests meaningful inconsistency in the network; a value < 5 supports the consistency assumption.
I²: < 25%: low heterogeneity; 25%–75% = moderate heterogeneity; > 75% = high heterogeneity.
Note: The "ume" designation refers to the unrelated mean effects model, which is termed the "inconsistency model" in this report.
