# Supplementary material for: Comparison of efficacy and safety of non-oral therapeutic interventions for zoster-associated pain: a systematic review and network meta-analysis
Source: Front Neurol. 2026 Jan 27;17:1711536. doi: 10.3389/fneur.2026.1711536 (PMC12886049; doi:10.3389/fneur.2026.1711536)
Supplement: Supplementary file 1 [file Data_Sheet_1.zip › Supplementary_Material_Complete/Table 2.docx]

**Table S2** Statistically significant effect modifiers identified in bayesian meta-regression

| **Outcome** | **Covariate** | **β (95% CrI)** | **Variable Type** |
| --- | --- | --- | --- |
| Pain relief | Sample size | -2.059 (-4.177, -0.002) | Continuous |
| Pain relief | Sample size | -4.321 (-6.619, -2.019) | Continuous |
| Pain relief | Baseline pain score | 4.011 (0.441, 7.645) | Continuous |
| Pain relief | Disease stage | 2.593 (0.199, 4.991) | Categorical |
| Pain relief | Follow-up duration | -2.538 (-4.418, -0.588) | Continuous |
| Pain relief | Follow-up duration | -4.533 (-7.598, -1.425) | Continuous |
| Pain relief | Age | 5.563 (0.938, 10.364) | Continuous |
| Pain relief | Female proportion | 3.706 (0.345, 7.292) | Continuous |
| Pain relief | Female proportion | 12.162 (6.328, 17.517) | Continuous |
| Adverse events | Baseline pain score | -2.709 (-4.714, -0.565) | Continuous |
| Adverse events | Sample size | 1.811 (0.658, 3.093) | Continuous |

**Abbreviations**: 95% CrI, 95% credible interval; β, regression coefficient. A modifying effect was significant if 95% CrI for β excluded the null value (0 for SMD and log(RR)); variable type is defined as Continuous (numerical scale, e.g., age, pain score) or Categorical (distinct groups, e.g., disease stage), and this table presents only significant covariates, with complete results available from the corresponding author upon request.
