# Supplementary material for: Comparison of efficacy and safety of non-oral therapeutic interventions for zoster-associated pain: a systematic review and network meta-analysis
Source: Front Neurol. 2026 Jan 27;17:1711536. doi: 10.3389/fneur.2026.1711536 (PMC12886049; doi:10.3389/fneur.2026.1711536)
Supplement: Supplementary file 1 [file Data_Sheet_1.zip › Supplementary_Material_Complete/Table 3.docx]

**Table S3** Results of Egger’s regression test for publication bias

| **Outcome** | **Beta (β)** | **SE** | **t-value** | ***P*-value** | **Evidence of asymmetry** |
| --- | --- | --- | --- | --- | --- |
| Pain relief | 0.53 | 1.51 | 0.36 | 0.72 | no |
| Sleep quality | -4.85 | 3.31 | -1.47 | 0.158 | no |
| Adverse events | -0.09 | 0.53 | -0.16 | 0.87 | no |

Evidence of asymmetry is considered statistically significant if *P* < 0.05.
