# Supplementary material for: Comparison of efficacy and safety of non-oral therapeutic interventions for zoster-associated pain: a systematic review and network meta-analysis
Source: Front Neurol. 2026 Jan 27;17:1711536. doi: 10.3389/fneur.2026.1711536 (PMC12886049; doi:10.3389/fneur.2026.1711536)
Supplement: Supplementary file 1 [file Data_Sheet_1.zip › Supplementary_Material_Complete/Table 4.docx]

**Table S4** Detailed CINeMA assessments for all pairwise comparisons of the three primary outcomes

**A. Pain Relief**

| **Comparison** | **Number of studies** | **Within-study bias** | **Reporting bias** | **Indirectness** | **Imprecision** | **Heterogeneity** | **Incoherence** | **Confidence rating** |
| --- | --- | --- | --- | --- | --- | --- | --- | --- |
| **Mixed evidence** | | | | | | | | |
| BioTx:ST | 2 | Some concerns | Low risk | No concerns | Major concerns | No concerns | No concerns | Low |
| BioTx:Sham | 1 | Some concerns | Low risk | No concerns | Major concerns | No concerns | No concerns | Low |
| CAM:ST | 1 | Some concerns | Low risk | No concerns | Major concerns | No concerns | No concerns | Low |
| CSN:NB | 1 | Some concerns | Low risk | No concerns | No concerns | No concerns | No concerns | Moderate |
| MI-CNS-NM:MI-CNS-NM + TPCI | 1 | Some concerns | Low risk | No concerns | Major concerns | No concerns | No concerns | Low |
| MI-CNS-NM:MI-PNM | 5 | No concerns | Low risk | No concerns | No concerns | Major concerns | No concerns | Low |
| MI-CNS-NM:NB | 1 | Some concerns | Low risk | No concerns | No concerns | Major concerns | No concerns | Low |
| MI-PNM:MI-PNM + MOT | 1 | Some concerns | Low risk | No concerns | Major concerns | No concerns | No concerns | Low |
| MI-PNM:MI-PNM + NB | 1 | No concerns | Low risk | No concerns | Major concerns | No concerns | No concerns | Low |
| MI-PNM:MI-PNM + SPA | 1 | Some concerns | Low risk | No concerns | No concerns | Major concerns | No concerns | Low |
| MI-PNM:ST | 2 | Some concerns | Low risk | No concerns | No concerns | Major concerns | No concerns | Low |
| MI-PNM:Sham | 7 | Some concerns | Low risk | No concerns | No concerns | Major concerns | No concerns | Low |
| MI-PNM:T-PNES | 1 | No concerns | Low risk | No concerns | Major concerns | No concerns | No concerns | Low |
| MI-PNM:TPCI | 1 | Some concerns | Low risk | No concerns | Major concerns | No concerns | No concerns | Low |
| MI-PNM + NB:NB | 1 | Some concerns | Low risk | No concerns | Major concerns | No concerns | No concerns | Low |
| MI-PNM + NB:Sham | 1 | Some concerns | Low risk | No concerns | No concerns | No concerns | No concerns | Moderate |
| MOT:ST | 2 | Some concerns | Low risk | No concerns | Major concerns | No concerns | No concerns | Low |
| MOT + NB:ST | 1 | Some concerns | Low risk | No concerns | Major concerns | No concerns | No concerns | Low |
| NB:PAN + NB | 1 | Some concerns | Low risk | No concerns | Major concerns | No concerns | No concerns | Low |
| NB:PTEM | 1 | Some concerns | Low risk | No concerns | Major concerns | No concerns | No concerns | Low |
| NB:PTEM + NB | 1 | Some concerns | Low risk | No concerns | Major concerns | No concerns | No concerns | Low |
| NB:ST | 8 | Some concerns | Low risk | No concerns | No concerns | Major concerns | No concerns | Low |
| NB:Sham | 6 | Some concerns | Low risk | No concerns | No concerns | Major concerns | No concerns | Low |
| NB:T-PNES | 1 | Some concerns | Low risk | No concerns | Major concerns | No concerns | No concerns | Low |
| NB:TPCI | 1 | Some concerns | Low risk | No concerns | Major concerns | No concerns | No concerns | Low |
| NI-CNS-NM:Sham | 2 | No concerns | Low risk | No concerns | No concerns | No concerns | No concerns | High |
| PTEM:SEN | 1 | No concerns | Low risk | No concerns | Major concerns | No concerns | No concerns | Low |
| PTEM:ST | 3 | No concerns | Low risk | No concerns | No concerns | Major concerns | No concerns | Low |
| Sham:SPA | 1 | Some concerns | Low risk | No concerns | Major concerns | No concerns | No concerns | Low |
| ST:TPCI | 4 | Some concerns | Low risk | No concerns | No concerns | Major concerns | No concerns | Low |
| Sham:TPCI | 2 | Some concerns | Low risk | No concerns | No concerns | Major concerns | No concerns | Low |
| **Indirect evidence** | | | | | | | | |
| BioTx:CAM | 0 | Some concerns | Low risk | No concerns | Major concerns | No concerns | No concerns | Low |
| BioTx:CSN | 0 | Some concerns | Low risk | No concerns | No concerns | No concerns | No concerns | Moderate |
| BioTx:MI-CNS-NM | 0 | Some concerns | Low risk | No concerns | No concerns | No concerns | No concerns | Moderate |
| BioTx:MI-CNS-NM + TPCI | 0 | Some concerns | Low risk | No concerns | No concerns | No concerns | No concerns | Moderate |
| BioTx:MI-PNM | 0 | Some concerns | Low risk | No concerns | Major concerns | No concerns | No concerns | Low |
| BioTx:MI-PNM + MOT | 0 | Some concerns | Low risk | No concerns | Major concerns | No concerns | No concerns | Low |
| BioTx:MI-PNM + NB | 0 | Some concerns | Low risk | No concerns | No concerns | Major concerns | No concerns | Low |
| BioTx:MI-PNM + SPA | 0 | Some concerns | Low risk | No concerns | No concerns | No concerns | No concerns | Moderate |
| BioTx:MOT | 0 | Some concerns | Low risk | No concerns | Major concerns | No concerns | No concerns | Low |
| BioTx:MOT + NB | 0 | Some concerns | Low risk | No concerns | Major concerns | No concerns | No concerns | Low |
| BioTx:NB | 0 | Some concerns | Low risk | No concerns | Major concerns | No concerns | No concerns | Low |
| BioTx:NI-CNS-NM | 0 | No concerns | Low risk | No concerns | Major concerns | No concerns | No concerns | Low |
| BioTx:PAN + NB | 0 | Some concerns | Low risk | No concerns | No concerns | Major concerns | No concerns | Low |
| BioTx:PTEM | 0 | Some concerns | Low risk | No concerns | Major concerns | No concerns | No concerns | Low |
| BioTx:PTEM + NB | 0 | Some concerns | Low risk | No concerns | Major concerns | No concerns | No concerns | Low |
| BioTx:SEN | 0 | No concerns | Low risk | No concerns | Major concerns | No concerns | No concerns | Low |
| BioTx:SPA | 0 | Some concerns | Low risk | No concerns | Major concerns | No concerns | No concerns | Low |
| BioTx:T-PNES | 0 | Some concerns | Low risk | No concerns | No concerns | Major concerns | No concerns | Low |
| BioTx:TPCI | 0 | Some concerns | Low risk | No concerns | Major concerns | No concerns | No concerns | Low |
| CAM:CSN | 0 | Some concerns | Low risk | No concerns | No concerns | No concerns | No concerns | Moderate |
| CAM:MI-CNS-NM | 0 | Some concerns | Low risk | No concerns | Major concerns | No concerns | No concerns | Low |
| CAM:MI-CNS-NM + TPCI | 0 | Some concerns | Low risk | No concerns | No concerns | No concerns | No concerns | Moderate |
| CAM:MI-PNM | 0 | Some concerns | Low risk | No concerns | Major concerns | No concerns | No concerns | Low |
| CAM:MI-PNM + MOT | 0 | Some concerns | Low risk | No concerns | Major concerns | No concerns | No concerns | Low |
| CAM:MI-PNM + NB | 0 | Some concerns | Low risk | No concerns | Major concerns | No concerns | No concerns | Low |
| CAM:MI-PNM + SPA | 0 | Some concerns | Low risk | No concerns | Major concerns | No concerns | No concerns | Low |
| CAM:MOT | 0 | Some concerns | Low risk | No concerns | Major concerns | No concerns | No concerns | Low |
| CAM:MOT + NB | 0 | Some concerns | Low risk | No concerns | Major concerns | No concerns | No concerns | Low |
| CAM:NB | 0 | Some concerns | Low risk | No concerns | Major concerns | No concerns | No concerns | Low |
| CAM:NI-CNS-NM | 0 | Some concerns | Low risk | No concerns | Major concerns | No concerns | No concerns | Low |
| CAM:PAN + NB | 0 | Some concerns | Low risk | No concerns | Major concerns | No concerns | No concerns | Low |
| CAM:PTEM | 0 | Some concerns | Low risk | No concerns | Major concerns | No concerns | No concerns | Low |
| CAM:PTEM + NB | 0 | Some concerns | Low risk | No concerns | Major concerns | No concerns | No concerns | Low |
| CAM:SEN | 0 | Some concerns | Low risk | No concerns | Major concerns | No concerns | No concerns | Low |
| CAM:SPA | 0 | Some concerns | Low risk | No concerns | Major concerns | No concerns | No concerns | Low |
| CAM:Sham | 0 | Some concerns | Low risk | No concerns | Major concerns | No concerns | No concerns | Low |
| CAM:T-PNES | 0 | Some concerns | Low risk | No concerns | Major concerns | No concerns | No concerns | Low |
| CAM:TPCI | 0 | Some concerns | Low risk | No concerns | Major concerns | No concerns | No concerns | Low |
| CSN:MI-CNS-NM | 0 | Some concerns | Low risk | No concerns | No concerns | No concerns | No concerns | Moderate |
| CSN:MI-CNS-NM + TPCI | 0 | Some concerns | Low risk | No concerns | No concerns | No concerns | No concerns | Moderate |
| CSN:MI-PNM | 0 | Some concerns | Low risk | No concerns | No concerns | No concerns | No concerns | Moderate |
| CSN:MI-PNM + MOT | 0 | Some concerns | Low risk | No concerns | No concerns | No concerns | No concerns | Moderate |
| CSN:MI-PNM + NB | 0 | Some concerns | Low risk | No concerns | No concerns | No concerns | No concerns | Moderate |
| CSN:MI-PNM + SPA | 0 | Some concerns | Low risk | No concerns | No concerns | No concerns | No concerns | Moderate |
| CSN:MOT | 0 | Some concerns | Low risk | No concerns | No concerns | No concerns | No concerns | Moderate |
| CSN:MOT + NB | 0 | Some concerns | Low risk | No concerns | No concerns | No concerns | No concerns | Moderate |
| CSN:NI-CNS-NM | 0 | Some concerns | Low risk | No concerns | No concerns | No concerns | No concerns | Moderate |
| CSN:PAN + NB | 0 | Some concerns | Low risk | No concerns | No concerns | No concerns | No concerns | Moderate |
| CSN:PTEM | 0 | Some concerns | Low risk | No concerns | No concerns | No concerns | No concerns | Moderate |
| CSN:PTEM + NB | 0 | Some concerns | Low risk | No concerns | No concerns | No concerns | No concerns | Moderate |
| CSN:SEN | 0 | Some concerns | Low risk | No concerns | No concerns | No concerns | No concerns | Moderate |
| CSN:SPA | 0 | Some concerns | Low risk | No concerns | No concerns | No concerns | No concerns | Moderate |
| CSN:ST | 0 | Some concerns | Low risk | No concerns | No concerns | No concerns | No concerns | Moderate |
| CSN:Sham | 0 | Some concerns | Low risk | No concerns | No concerns | No concerns | No concerns | Moderate |
| CSN:T-PNES | 0 | Some concerns | Low risk | No concerns | No concerns | No concerns | No concerns | Moderate |
| CSN:TPCI | 0 | Some concerns | Low risk | No concerns | No concerns | No concerns | No concerns | Moderate |
| MI-CNS-NM:MI-PNM + MOT | 0 | Some concerns | Low risk | No concerns | Major concerns | No concerns | No concerns | Low |
| MI-CNS-NM:MI-PNM + NB | 0 | No concerns | Low risk | No concerns | Major concerns | No concerns | No concerns | Low |
| MI-CNS-NM:MI-PNM + SPA | 0 | Some concerns | Low risk | No concerns | Major concerns | No concerns | No concerns | Low |
| MI-CNS-NM:MOT | 0 | Some concerns | Low risk | No concerns | No concerns | Major concerns | No concerns | Low |
| MI-CNS-NM:MOT + NB | 0 | Some concerns | Low risk | No concerns | Major concerns | No concerns | No concerns | Low |
| MI-CNS-NM:NI-CNS-NM | 0 | No concerns | Low risk | No concerns | Major concerns | No concerns | No concerns | Low |
| MI-CNS-NM:PAN + NB | 0 | Some concerns | Low risk | No concerns | Major concerns | No concerns | No concerns | Low |
| MI-CNS-NM:PTEM | 0 | Some concerns | Low risk | No concerns | No concerns | Major concerns | No concerns | Low |
| MI-CNS-NM:PTEM + NB | 0 | Some concerns | Low risk | No concerns | Major concerns | No concerns | No concerns | Low |
| MI-CNS-NM:SEN | 0 | Some concerns | Low risk | No concerns | Major concerns | No concerns | No concerns | Low |
| MI-CNS-NM:SPA | 0 | Some concerns | Low risk | No concerns | Major concerns | No concerns | No concerns | Low |
| MI-CNS-NM:ST | 0 | Some concerns | Low risk | No concerns | No concerns | No concerns | No concerns | Moderate |
| MI-CNS-NM:Sham | 0 | No concerns | Low risk | No concerns | No concerns | No concerns | No concerns | High |
| MI-CNS-NM:T-PNES | 0 | No concerns | Low risk | No concerns | Major concerns | No concerns | No concerns | Low |
| MI-CNS-NM:TPCI | 0 | Some concerns | Low risk | No concerns | No concerns | Major concerns | No concerns | Low |
| MI-CNS-NM + TPCI:MI-PNM | 0 | Some concerns | Low risk | No concerns | No concerns | No concerns | No concerns | Moderate |
| MI-CNS-NM + TPCI:MI-PNM + MOT | 0 | Some concerns | Low risk | No concerns | Major concerns | No concerns | No concerns | Low |
| MI-CNS-NM + TPCI:MI-PNM + NB | 0 | Some concerns | Low risk | No concerns | Major concerns | No concerns | No concerns | Low |
| MI-CNS-NM + TPCI:MI-PNM + SPA | 0 | Some concerns | Low risk | No concerns | Major concerns | No concerns | No concerns | Low |
| MI-CNS-NM + TPCI:MOT | 0 | Some concerns | Low risk | No concerns | No concerns | No concerns | No concerns | Moderate |
| MI-CNS-NM + TPCI:MOT + NB | 0 | Some concerns | Low risk | No concerns | Major concerns | No concerns | No concerns | Low |
| MI-CNS-NM + TPCI:NB | 0 | Some concerns | Low risk | No concerns | No concerns | No concerns | No concerns | Moderate |
| MI-CNS-NM + TPCI:NI-CNS-NM | 0 | No concerns | Low risk | No concerns | Major concerns | No concerns | No concerns | Low |
| MI-CNS-NM + TPCI:PAN + NB | 0 | Some concerns | Low risk | No concerns | Major concerns | No concerns | No concerns | Low |
| MI-CNS-NM + TPCI:PTEM | 0 | Some concerns | Low risk | No concerns | No concerns | No concerns | No concerns | Moderate |
| MI-CNS-NM + TPCI:PTEM + NB | 0 | Some concerns | Low risk | No concerns | Major concerns | No concerns | No concerns | Low |
| MI-CNS-NM + TPCI:SEN | 0 | Some concerns | Low risk | No concerns | Major concerns | No concerns | No concerns | Low |
| MI-CNS-NM + TPCI:SPA | 0 | Some concerns | Low risk | No concerns | No concerns | Major concerns | No concerns | Low |
| MI-CNS-NM + TPCI:ST | 0 | Some concerns | Low risk | No concerns | No concerns | No concerns | No concerns | Moderate |
| MI-CNS-NM + TPCI:Sham | 0 | Some concerns | Low risk | No concerns | No concerns | No concerns | No concerns | Moderate |
| MI-CNS-NM + TPCI:T-PNES | 0 | Some concerns | Low risk | No concerns | Major concerns | No concerns | No concerns | Low |
| MI-CNS-NM + TPCI:TPCI | 0 | Some concerns | Low risk | No concerns | No concerns | Major concerns | No concerns | Low |
| MI-PNM:MOT | 0 | Some concerns | Low risk | No concerns | Major concerns | No concerns | No concerns | Low |
| MI-PNM:MOT + NB | 0 | Some concerns | Low risk | No concerns | Major concerns | No concerns | No concerns | Low |
| MI-PNM:NB | 0 | Some concerns | Low risk | No concerns | Major concerns | No concerns | No concerns | Low |
| MI-PNM:NI-CNS-NM | 0 | No concerns | Low risk | No concerns | Major concerns | No concerns | No concerns | Low |
| MI-PNM:PAN + NB | 0 | Some concerns | Low risk | No concerns | Major concerns | No concerns | No concerns | Low |
| MI-PNM:PTEM | 0 | Some concerns | Low risk | No concerns | Major concerns | No concerns | No concerns | Low |
| MI-PNM:PTEM + NB | 0 | Some concerns | Low risk | No concerns | Major concerns | No concerns | No concerns | Low |
| MI-PNM:SEN | 0 | Some concerns | Low risk | No concerns | Major concerns | No concerns | No concerns | Low |
| MI-PNM:SPA | 0 | Some concerns | Low risk | No concerns | Major concerns | No concerns | No concerns | Low |
| MI-PNM + MOT:MI-PNM + NB | 0 | Some concerns | Low risk | No concerns | Major concerns | No concerns | No concerns | Low |
| MI-PNM + MOT:MI-PNM + SPA | 0 | Some concerns | Low risk | No concerns | Major concerns | No concerns | No concerns | Low |
| MI-PNM + MOT:MOT | 0 | Some concerns | Low risk | No concerns | Major concerns | No concerns | No concerns | Low |
| MI-PNM + MOT:MOT + NB | 0 | Some concerns | Low risk | No concerns | Major concerns | No concerns | No concerns | Low |
| MI-PNM + MOT:NB | 0 | Some concerns | Low risk | No concerns | Major concerns | No concerns | No concerns | Low |
| MI-PNM + MOT:NI-CNS-NM | 0 | Some concerns | Low risk | No concerns | Major concerns | No concerns | No concerns | Low |
| MI-PNM + MOT:PAN + NB | 0 | Some concerns | Low risk | No concerns | Major concerns | No concerns | No concerns | Low |
| MI-PNM + MOT:PTEM | 0 | Some concerns | Low risk | No concerns | Major concerns | No concerns | No concerns | Low |
| MI-PNM + MOT:PTEM + NB | 0 | Some concerns | Low risk | No concerns | Major concerns | No concerns | No concerns | Low |
| MI-PNM + MOT:SEN | 0 | Some concerns | Low risk | No concerns | Major concerns | No concerns | No concerns | Low |
| MI-PNM + MOT:SPA | 0 | Some concerns | Low risk | No concerns | Major concerns | No concerns | No concerns | Low |
| MI-PNM + MOT:ST | 0 | Some concerns | Low risk | No concerns | No concerns | Major concerns | No concerns | Low |
| MI-PNM + MOT:Sham | 0 | Some concerns | Low risk | No concerns | No concerns | Major concerns | No concerns | Low |
| MI-PNM + MOT:T-PNES | 0 | Some concerns | Low risk | No concerns | Major concerns | No concerns | No concerns | Low |
| MI-PNM + MOT:TPCI | 0 | Some concerns | Low risk | No concerns | Major concerns | No concerns | No concerns | Low |
| MI-PNM + NB:MI-PNM + SPA | 0 | Some concerns | Low risk | No concerns | Major concerns | No concerns | No concerns | Low |
| MI-PNM + NB:MOT | 0 | Some concerns | Low risk | No concerns | Major concerns | No concerns | No concerns | Low |
| MI-PNM + NB:MOT + NB | 0 | Some concerns | Low risk | No concerns | Major concerns | No concerns | No concerns | Low |
| MI-PNM + NB:NI-CNS-NM | 0 | No concerns | Low risk | No concerns | Major concerns | No concerns | No concerns | Low |
| MI-PNM + NB:PAN + NB | 0 | Some concerns | Low risk | No concerns | Major concerns | No concerns | No concerns | Low |
| MI-PNM + NB:PTEM | 0 | Some concerns | Low risk | No concerns | Major concerns | No concerns | No concerns | Low |
| MI-PNM + NB:PTEM + NB | 0 | Some concerns | Low risk | No concerns | Major concerns | No concerns | No concerns | Low |
| MI-PNM + NB:SEN | 0 | Some concerns | Low risk | No concerns | Major concerns | No concerns | No concerns | Low |
| MI-PNM + NB:SPA | 0 | Some concerns | Low risk | No concerns | Major concerns | No concerns | No concerns | Low |
| MI-PNM + NB:ST | 0 | Some concerns | Low risk | No concerns | No concerns | No concerns | No concerns | Moderate |
| MI-PNM + NB:T-PNES | 0 | Some concerns | Low risk | No concerns | Major concerns | No concerns | No concerns | Low |
| MI-PNM + NB:TPCI | 0 | Some concerns | Low risk | No concerns | Major concerns | No concerns | No concerns | Low |
| MI-PNM + SPA:MOT | 0 | Some concerns | Low risk | No concerns | No concerns | Major concerns | No concerns | Low |
| MI-PNM + SPA:MOT + NB | 0 | Some concerns | Low risk | No concerns | Major concerns | No concerns | No concerns | Low |
| MI-PNM + SPA:NB | 0 | Some concerns | Low risk | No concerns | No concerns | Major concerns | No concerns | Low |
| MI-PNM + SPA:NI-CNS-NM | 0 | Some concerns | Low risk | No concerns | Major concerns | No concerns | No concerns | Low |
| MI-PNM + SPA:PAN + NB | 0 | Some concerns | Low risk | No concerns | Major concerns | No concerns | No concerns | Low |
| MI-PNM + SPA:PTEM | 0 | Some concerns | Low risk | No concerns | Major concerns | No concerns | No concerns | Low |
| MI-PNM + SPA:PTEM + NB | 0 | Some concerns | Low risk | No concerns | Major concerns | No concerns | No concerns | Low |
| MI-PNM + SPA:SEN | 0 | Some concerns | Low risk | No concerns | Major concerns | No concerns | No concerns | Low |
| MI-PNM + SPA:SPA | 0 | Some concerns | Low risk | No concerns | Major concerns | No concerns | No concerns | Low |
| MI-PNM + SPA:ST | 0 | Some concerns | Low risk | No concerns | No concerns | No concerns | No concerns | Moderate |
| MI-PNM + SPA:Sham | 0 | Some concerns | Low risk | No concerns | No concerns | No concerns | No concerns | Moderate |
| MI-PNM + SPA:T-PNES | 0 | Some concerns | Low risk | No concerns | Major concerns | No concerns | No concerns | Low |
| MI-PNM + SPA:TPCI | 0 | Some concerns | Low risk | No concerns | Major concerns | No concerns | No concerns | Low |
| MOT:MOT + NB | 0 | Some concerns | Low risk | No concerns | Major concerns | No concerns | No concerns | Low |
| MOT:NB | 0 | Some concerns | Low risk | No concerns | Major concerns | No concerns | No concerns | Low |
| MOT:NI-CNS-NM | 0 | Some concerns | Low risk | No concerns | Major concerns | No concerns | No concerns | Low |
| MOT:PAN + NB | 0 | Some concerns | Low risk | No concerns | Major concerns | No concerns | No concerns | Low |
| MOT:PTEM | 0 | Some concerns | Low risk | No concerns | Major concerns | No concerns | No concerns | Low |
| MOT:PTEM + NB | 0 | Some concerns | Low risk | No concerns | Major concerns | No concerns | No concerns | Low |
| MOT:SEN | 0 | Some concerns | Low risk | No concerns | Major concerns | No concerns | No concerns | Low |
| MOT:SPA | 0 | Some concerns | Low risk | No concerns | Major concerns | No concerns | No concerns | Low |
| MOT:Sham | 0 | Some concerns | Low risk | No concerns | Major concerns | No concerns | No concerns | Low |
| MOT:T-PNES | 0 | Some concerns | Low risk | No concerns | Major concerns | No concerns | No concerns | Low |
| MOT:TPCI | 0 | Some concerns | Low risk | No concerns | Major concerns | No concerns | No concerns | Low |
| MOT + NB:NB | 0 | Some concerns | Low risk | No concerns | Major concerns | No concerns | No concerns | Low |
| MOT + NB:NI-CNS-NM | 0 | Some concerns | Low risk | No concerns | Major concerns | No concerns | No concerns | Low |
| MOT + NB:PAN + NB | 0 | Some concerns | Low risk | No concerns | Major concerns | No concerns | No concerns | Low |
| MOT + NB:PTEM | 0 | Some concerns | Low risk | No concerns | Major concerns | No concerns | No concerns | Low |
| MOT + NB:PTEM + NB | 0 | Some concerns | Low risk | No concerns | Major concerns | No concerns | No concerns | Low |
| MOT + NB:SEN | 0 | Some concerns | Low risk | No concerns | Major concerns | No concerns | No concerns | Low |
| MOT + NB:SPA | 0 | Some concerns | Low risk | No concerns | Major concerns | No concerns | No concerns | Low |
| MOT + NB:Sham | 0 | Some concerns | Low risk | No concerns | Major concerns | No concerns | No concerns | Low |
| MOT + NB:T-PNES | 0 | Some concerns | Low risk | No concerns | Major concerns | No concerns | No concerns | Low |
| MOT + NB:TPCI | 0 | Some concerns | Low risk | No concerns | Major concerns | No concerns | No concerns | Low |
| NB:NI-CNS-NM | 0 | No concerns | Low risk | No concerns | Major concerns | No concerns | No concerns | Low |
| NB:SEN | 0 | No concerns | Low risk | No concerns | Major concerns | No concerns | No concerns | Low |
| NB:SPA | 0 | Some concerns | Low risk | No concerns | Major concerns | No concerns | No concerns | Low |
| NI-CNS-NM:PAN + NB | 0 | Some concerns | Low risk | No concerns | Major concerns | No concerns | No concerns | Low |
| NI-CNS-NM:PTEM | 0 | Some concerns | Low risk | No concerns | Major concerns | No concerns | No concerns | Low |
| NI-CNS-NM:PTEM + NB | 0 | Some concerns | Low risk | No concerns | Major concerns | No concerns | No concerns | Low |
| NI-CNS-NM:SEN | 0 | No concerns | Low risk | No concerns | Major concerns | No concerns | No concerns | Low |
| NI-CNS-NM:SPA | 0 | Some concerns | Low risk | No concerns | Major concerns | No concerns | No concerns | Low |
| NI-CNS-NM:ST | 0 | Some concerns | Low risk | No concerns | No concerns | Major concerns | No concerns | Low |
| NI-CNS-NM:T-PNES | 0 | No concerns | Low risk | No concerns | Major concerns | No concerns | No concerns | Low |
| NI-CNS-NM:TPCI | 0 | No concerns | Low risk | No concerns | Major concerns | No concerns | No concerns | Low |
| PAN + NB:PTEM | 0 | Some concerns | Low risk | No concerns | Major concerns | No concerns | No concerns | Low |
| PAN + NB:PTEM + NB | 0 | Some concerns | Low risk | No concerns | Major concerns | No concerns | No concerns | Low |
| PAN + NB:SEN | 0 | Some concerns | Low risk | No concerns | Major concerns | No concerns | No concerns | Low |
| PAN + NB:SPA | 0 | Some concerns | Low risk | No concerns | Major concerns | No concerns | No concerns | Low |
| PAN + NB:ST | 0 | Some concerns | Low risk | No concerns | No concerns | No concerns | No concerns | Moderate |
| PAN + NB:Sham | 0 | Some concerns | Low risk | No concerns | No concerns | No concerns | No concerns | Moderate |
| PAN + NB:T-PNES | 0 | Some concerns | Low risk | No concerns | Major concerns | No concerns | No concerns | Low |
| PAN + NB:TPCI | 0 | Some concerns | Low risk | No concerns | Major concerns | No concerns | No concerns | Low |
| PTEM:PTEM + NB | 0 | Some concerns | Low risk | No concerns | Major concerns | No concerns | No concerns | Low |
| PTEM:SPA | 0 | Some concerns | Low risk | No concerns | Major concerns | No concerns | No concerns | Low |
| PTEM:Sham | 0 | Some concerns | Low risk | No concerns | No concerns | Major concerns | No concerns | Low |
| PTEM:T-PNES | 0 | Some concerns | Low risk | No concerns | Major concerns | No concerns | No concerns | Low |
| PTEM:TPCI | 0 | Some concerns | Low risk | No concerns | Major concerns | No concerns | No concerns | Low |
| PTEM + NB:SEN | 0 | Some concerns | Low risk | No concerns | Major concerns | No concerns | No concerns | Low |
| PTEM + NB:SPA | 0 | Some concerns | Low risk | No concerns | Major concerns | No concerns | No concerns | Low |
| PTEM + NB:ST | 0 | Some concerns | Low risk | No concerns | No concerns | Major concerns | No concerns | Low |
| PTEM + NB:Sham | 0 | Some concerns | Low risk | No concerns | No concerns | Major concerns | No concerns | Low |
| PTEM + NB:T-PNES | 0 | Some concerns | Low risk | No concerns | Major concerns | No concerns | No concerns | Low |
| PTEM + NB:TPCI | 0 | Some concerns | Low risk | No concerns | Major concerns | No concerns | No concerns | Low |
| SEN:SPA | 0 | Some concerns | Low risk | No concerns | Major concerns | No concerns | No concerns | Low |
| SEN:ST | 0 | No concerns | Low risk | No concerns | Major concerns | No concerns | No concerns | Low |
| SEN:Sham | 0 | Some concerns | Low risk | No concerns | Major concerns | No concerns | No concerns | Low |
| SEN:T-PNES | 0 | Some concerns | Low risk | No concerns | Major concerns | No concerns | No concerns | Low |
| SEN:TPCI | 0 | No concerns | Low risk | No concerns | Major concerns | No concerns | No concerns | Low |
| SPA:ST | 0 | Some concerns | Low risk | No concerns | Major concerns | No concerns | No concerns | Low |
| SPA:T-PNES | 0 | Some concerns | Low risk | No concerns | Major concerns | No concerns | No concerns | Low |
| SPA:TPCI | 0 | Some concerns | Low risk | No concerns | Major concerns | No concerns | No concerns | Low |
| Sham:ST | 0 | Some concerns | Low risk | No concerns | Major concerns | No concerns | No concerns | Low |
| ST:T-PNES | 0 | Some concerns | Low risk | No concerns | No concerns | No concerns | No concerns | Moderate |
| Sham:T-PNES | 0 | Some concerns | Low risk | No concerns | No concerns | No concerns | No concerns | Moderate |
| TPCI:T-PNES | 0 | Some concerns | Low risk | No concerns | Major concerns | No concerns | No concerns | Low |

1. **Sleep quality**

| **Comparison** | **Number of studies** | **Within-study bias** | **Reporting bias** | **Indirectness** | **Imprecision** | **Heterogeneity** | **Incoherence** | **Confidence rating** |
| --- | --- | --- | --- | --- | --- | --- | --- | --- |
| **Mixed evidence** | | | | | | | | |
| MI-CNS-NM:MI-CNS-NM + TPCI | 1 | Some concerns | Low risk | No concerns | No concerns | Major concerns | No concerns | Low |
| MI-CNS-NM:MI-PNM | 2 | No concerns | Low risk | No concerns | No concerns | No concerns | No concerns | High |
| MI-CNS-NM:NB | 1 | Some concerns | Low risk | No concerns | No concerns | Major concerns | No concerns | Low |
| MI-PNM:MI-PNM + NB | 1 | No concerns | Low risk | No concerns | No concerns | Major concerns | No concerns | Low |
| MI-PNM:MI-PNM + SPA | 1 | Some concerns | Low risk | No concerns | Major concerns | No concerns | No concerns | Low |
| MI-PNM:ST | 2 | Some concerns | Low risk | No concerns | No concerns | Major concerns | No concerns | Low |
| MI-PNM:Sham | 3 | No concerns | Low risk | No concerns | No concerns | No concerns | No concerns | High |
| MI-PNM:TPCI | 1 | No concerns | Low risk | No concerns | Major concerns | No concerns | No concerns | Low |
| MI-PNM + NB:NB | 1 | Some concerns | Low risk | No concerns | No concerns | Major concerns | No concerns | Low |
| NB:PAN + NB | 1 | Some concerns | Low risk | No concerns | No concerns | Major concerns | No concerns | Low |
| NB:ST | 1 | Some concerns | Low risk | No concerns | No concerns | Major concerns | No concerns | Low |
| NB:T-PNES | 1 | Major concerns | Low risk | No concerns | Major concerns | No concerns | No concerns | Very low |
| NB:TPCI | 1 | No concerns | Low risk | No concerns | Major concerns | No concerns | No concerns | Low |
| NI-CNS-NM:Sham | 2 | No concerns | Low risk | No concerns | Major concerns | No concerns | No concerns | Low |
| PTEM:ST | 1 | No concerns | Low risk | No concerns | Major concerns | No concerns | No concerns | Low |
| Sham:SPA | 1 | Some concerns | Low risk | No concerns | No concerns | No concerns | No concerns | Moderate |
| Sham:TPCI | 1 | No concerns | Low risk | No concerns | No concerns | No concerns | No concerns | High |
| **Indirect evidence** | | | | | | | | |
| MI-CNS-NM:MI-PNM + NB | 0 | No concerns | Low risk | No concerns | Major concerns | No concerns | No concerns | Low |
| MI-CNS-NM:MI-PNM + SPA | 0 | Some concerns | Low risk | No concerns | Major concerns | No concerns | No concerns | Low |
| MI-CNS-NM:NI-CNS-NM | 0 | No concerns | Low risk | No concerns | No concerns | No concerns | No concerns | High |
| MI-CNS-NM:PAN + NB | 0 | Some concerns | Low risk | No concerns | Major concerns | No concerns | No concerns | Low |
| MI-CNS-NM:PTEM | 0 | Some concerns | Low risk | No concerns | Major concerns | No concerns | No concerns | Low |
| MI-CNS-NM:SPA | 0 | No concerns | Low risk | No concerns | No concerns | Major concerns | No concerns | Low |
| MI-CNS-NM:ST | 0 | Some concerns | Low risk | No concerns | No concerns | No concerns | No concerns | Moderate |
| MI-CNS-NM:Sham | 0 | No concerns | Low risk | No concerns | No concerns | No concerns | No concerns | High |
| MI-CNS-NM:T-PNES | 0 | Some concerns | Low risk | No concerns | Major concerns | No concerns | No concerns | Low |
| MI-CNS-NM:TPCI | 0 | No concerns | Low risk | No concerns | No concerns | Major concerns | No concerns | Low |
| MI-CNS-NM + TPCI:MI-PNM | 0 | Some concerns | Low risk | No concerns | No concerns | No concerns | No concerns | Moderate |
| MI-CNS-NM + TPCI:MI-PNM + NB | 0 | Some concerns | Low risk | No concerns | No concerns | Major concerns | No concerns | Low |
| MI-CNS-NM + TPCI:MI-PNM + SPA | 0 | Some concerns | Low risk | No concerns | No concerns | No concerns | No concerns | Moderate |
| MI-CNS-NM + TPCI:NB | 0 | Some concerns | Low risk | No concerns | No concerns | No concerns | No concerns | Moderate |
| MI-CNS-NM + TPCI:NI-CNS-NM | 0 | No concerns | Low risk | No concerns | No concerns | No concerns | No concerns | High |
| MI-CNS-NM + TPCI:PAN + NB | 0 | Some concerns | Low risk | No concerns | Major concerns | No concerns | No concerns | Low |
| MI-CNS-NM + TPCI:PTEM | 0 | Some concerns | Low risk | No concerns | Major concerns | No concerns | No concerns | Low |
| MI-CNS-NM + TPCI:SPA | 0 | Some concerns | Low risk | No concerns | No concerns | No concerns | No concerns | Moderate |
| MI-CNS-NM + TPCI:ST | 0 | Some concerns | Low risk | No concerns | No concerns | No concerns | No concerns | Moderate |
| MI-CNS-NM + TPCI:Sham | 0 | No concerns | Low risk | No concerns | No concerns | No concerns | No concerns | High |
| MI-CNS-NM + TPCI:T-PNES | 0 | Some concerns | Low risk | No concerns | Major concerns | No concerns | No concerns | Low |
| MI-CNS-NM + TPCI:TPCI | 0 | No concerns | Low risk | No concerns | No concerns | No concerns | No concerns | High |
| MI-PNM:NB | 0 | Some concerns | Low risk | No concerns | Major concerns | No concerns | No concerns | Low |
| MI-PNM:NI-CNS-NM | 0 | No concerns | Low risk | No concerns | No concerns | Major concerns | No concerns | Low |
| MI-PNM:PAN + NB | 0 | Some concerns | Low risk | No concerns | No concerns | No concerns | No concerns | Moderate |
| MI-PNM:PTEM | 0 | Some concerns | Low risk | No concerns | Major concerns | No concerns | No concerns | Low |
| MI-PNM:SPA | 0 | Some concerns | Low risk | No concerns | Major concerns | No concerns | No concerns | Low |
| MI-PNM:T-PNES | 0 | Some concerns | Low risk | No concerns | No concerns | Major concerns | No concerns | Low |
| MI-PNM + NB:MI-PNM + SPA | 0 | Some concerns | Low risk | No concerns | Major concerns | No concerns | No concerns | Low |
| MI-PNM + NB:NI-CNS-NM | 0 | No concerns | Low risk | No concerns | No concerns | No concerns | No concerns | High |
| MI-PNM + NB:PAN + NB | 0 | Some concerns | Low risk | No concerns | Major concerns | No concerns | No concerns | Low |
| MI-PNM + NB:PTEM | 0 | Some concerns | Low risk | No concerns | Major concerns | No concerns | No concerns | Low |
| MI-PNM + NB:SPA | 0 | Some concerns | Low risk | No concerns | Major concerns | No concerns | No concerns | Low |
| MI-PNM + NB:ST | 0 | Some concerns | Low risk | No concerns | No concerns | No concerns | No concerns | Moderate |
| MI-PNM + NB:Sham | 0 | No concerns | Low risk | No concerns | No concerns | No concerns | No concerns | High |
| MI-PNM + NB:T-PNES | 0 | Some concerns | Low risk | No concerns | Major concerns | No concerns | No concerns | Low |
| MI-PNM + NB:TPCI | 0 | No concerns | Low risk | No concerns | Major concerns | No concerns | No concerns | Low |
| MI-PNM + SPA:NB | 0 | Some concerns | Low risk | No concerns | Major concerns | No concerns | No concerns | Low |
| MI-PNM + SPA:NI-CNS-NM | 0 | No concerns | Low risk | No concerns | No concerns | Major concerns | No concerns | Low |
| MI-PNM + SPA:PAN + NB | 0 | Some concerns | Low risk | No concerns | Major concerns | No concerns | No concerns | Low |
| MI-PNM + SPA:PTEM | 0 | Some concerns | Low risk | No concerns | Major concerns | No concerns | No concerns | Low |
| MI-PNM + SPA:SPA | 0 | Some concerns | Low risk | No concerns | Major concerns | No concerns | No concerns | Low |
| MI-PNM + SPA:ST | 0 | Some concerns | Low risk | No concerns | No concerns | Major concerns | No concerns | Low |
| MI-PNM + SPA:Sham | 0 | Some concerns | Low risk | No concerns | No concerns | No concerns | No concerns | Moderate |
| MI-PNM + SPA:T-PNES | 0 | Some concerns | Low risk | No concerns | Major concerns | No concerns | No concerns | Low |
| MI-PNM + SPA:TPCI | 0 | Some concerns | Low risk | No concerns | Major concerns | No concerns | No concerns | Low |
| NB:NI-CNS-NM | 0 | No concerns | Low risk | No concerns | No concerns | Major concerns | No concerns | Low |
| NB:PTEM | 0 | Some concerns | Low risk | No concerns | Major concerns | No concerns | No concerns | Low |
| NB:SPA | 0 | Some concerns | Low risk | No concerns | Major concerns | No concerns | No concerns | Low |
| NB:Sham | 0 | No concerns | Low risk | No concerns | No concerns | No concerns | No concerns | High |
| NI-CNS-NM:PAN + NB | 0 | No concerns | Low risk | No concerns | No concerns | No concerns | No concerns | High |
| NI-CNS-NM:PTEM | 0 | No concerns | Low risk | No concerns | Major concerns | No concerns | No concerns | Low |
| NI-CNS-NM:SPA | 0 | Some concerns | Low risk | No concerns | No concerns | Major concerns | No concerns | Low |
| NI-CNS-NM:ST | 0 | Some concerns | Low risk | No concerns | Major concerns | No concerns | No concerns | Low |
| NI-CNS-NM:T-PNES | 0 | Some concerns | Low risk | No concerns | No concerns | No concerns | No concerns | Moderate |
| NI-CNS-NM:TPCI | 0 | No concerns | Low risk | No concerns | No concerns | Major concerns | No concerns | Low |
| PAN + NB:PTEM | 0 | Some concerns | Low risk | No concerns | Major concerns | No concerns | No concerns | Low |
| PAN + NB:SPA | 0 | Some concerns | Low risk | No concerns | No concerns | Major concerns | No concerns | Low |
| PAN + NB:ST | 0 | Some concerns | Low risk | No concerns | No concerns | No concerns | No concerns | Moderate |
| PAN + NB:Sham | 0 | Some concerns | Low risk | No concerns | No concerns | No concerns | No concerns | Moderate |
| PAN + NB:T-PNES | 0 | Major concerns | Low risk | No concerns | Major concerns | No concerns | No concerns | Very low |
| PAN + NB:TPCI | 0 | Some concerns | Low risk | No concerns | No concerns | Major concerns | No concerns | Low |
| PTEM:SPA | 0 | Some concerns | Low risk | No concerns | Major concerns | No concerns | No concerns | Low |
| PTEM:Sham | 0 | Some concerns | Low risk | No concerns | Major concerns | No concerns | No concerns | Low |
| PTEM:T-PNES | 0 | Some concerns | Low risk | No concerns | Major concerns | No concerns | No concerns | Low |
| PTEM:TPCI | 0 | No concerns | Low risk | No concerns | Major concerns | No concerns | No concerns | Low |
| SPA:ST | 0 | Some concerns | Low risk | No concerns | Major concerns | No concerns | No concerns | Low |
| SPA:T-PNES | 0 | Some concerns | Low risk | No concerns | Major concerns | No concerns | No concerns | Low |
| SPA:TPCI | 0 | No concerns | Low risk | No concerns | Major concerns | No concerns | No concerns | Low |
| Sham:ST | 0 | Some concerns | Low risk | No concerns | No concerns | Major concerns | No concerns | Low |
| ST:T-PNES | 0 | Some concerns | Low risk | No concerns | No concerns | No concerns | No concerns | Moderate |
| ST:TPCI | 0 | Some concerns | Low risk | No concerns | No concerns | Major concerns | No concerns | Low |
| Sham:T-PNES | 0 | Some concerns | Low risk | No concerns | No concerns | No concerns | No concerns | Moderate |
| TPCI:T-PNES | 0 | Some concerns | Low risk | No concerns | Major concerns | No concerns | No concerns | Low |

1. **Adverse events**

| **Comparison** | **Number of studies** | **Within-study bias** | **Reporting bias** | **Indirectness** | **Imprecision** | **Heterogeneity** | **Incoherence** | **Confidence rating** |
| --- | --- | --- | --- | --- | --- | --- | --- | --- |
| **Mixed evidence** | | | | | | | | |
| BioTx:ST | 1 | Some concerns | Low risk | No concerns | Major concerns | No concerns | No concerns | Low |
| CSN:NB | 1 | Some concerns | Low risk | No concerns | No concerns | Major concerns | No concerns | Low |
| MI-CNS-NM:MI-PNM | 3 | No concerns | Low risk | No concerns | Major concerns | No concerns | No concerns | Low |
| MI-PNM:MI-PNM + SPA | 1 | Some concerns | Low risk | No concerns | Major concerns | No concerns | No concerns | Low |
| MI-PNM:Sham | 3 | Some concerns | Low risk | No concerns | Major concerns | No concerns | No concerns | Low |
| MI-PNM:T-PNES | 1 | No concerns | Low risk | No concerns | Major concerns | No concerns | No concerns | Low |
| MI-PNM:TPCI | 1 | No concerns | Low risk | No concerns | Major concerns | No concerns | No concerns | Low |
| MI-PNM + NB:NB | 1 | Some concerns | Low risk | No concerns | Major concerns | No concerns | No concerns | Low |
| MOT:ST | 1 | Some concerns | Low risk | No concerns | Major concerns | No concerns | No concerns | Low |
| MOT + NB:ST | 1 | Some concerns | Low risk | No concerns | Major concerns | No concerns | No concerns | Low |
| NB:PAN + NB | 1 | Some concerns | Low risk | No concerns | Major concerns | No concerns | No concerns | Low |
| NB:ST | 6 | Some concerns | Low risk | No concerns | Major concerns | No concerns | No concerns | Low |
| NB:Sham | 1 | No concerns | Low risk | No concerns | Major concerns | No concerns | No concerns | Low |
| NB:T-PNES | 1 | Major concerns | Low risk | No concerns | Major concerns | No concerns | No concerns | Very low |
| NB:TPCI | 1 | No concerns | Low risk | No concerns | Major concerns | No concerns | No concerns | Low |
| PTEM:ST | 2 | No concerns | Low risk | No concerns | Major concerns | No concerns | No concerns | Low |
| Sham:SPA | 1 | Some concerns | Low risk | No concerns | Major concerns | No concerns | No concerns | Low |
| Sham:TPCI | 1 | No concerns | Low risk | No concerns | Major concerns | No concerns | No concerns | Low |
| **Indirect evidence** | | | | | | | | |
| BioTx:CSN | 0 | Some concerns | Low risk | No concerns | Major concerns | No concerns | No concerns | Low |
| BioTx:MI-CNS-NM | 0 | Some concerns | Low risk | No concerns | Major concerns | No concerns | No concerns | Low |
| BioTx:MI-PNM | 0 | Some concerns | Low risk | No concerns | Major concerns | No concerns | No concerns | Low |
| BioTx:MI-PNM + NB | 0 | Some concerns | Low risk | No concerns | Major concerns | No concerns | No concerns | Low |
| BioTx:MI-PNM + SPA | 0 | Some concerns | Low risk | No concerns | Major concerns | No concerns | No concerns | Low |
| BioTx:MOT | 0 | Some concerns | Low risk | No concerns | Major concerns | No concerns | No concerns | Low |
| BioTx:MOT + NB | 0 | Some concerns | Low risk | No concerns | Major concerns | No concerns | No concerns | Low |
| BioTx:NB | 0 | Some concerns | Low risk | No concerns | Major concerns | No concerns | No concerns | Low |
| BioTx:PAN + NB | 0 | Some concerns | Low risk | No concerns | Major concerns | No concerns | No concerns | Low |
| BioTx:PTEM | 0 | Some concerns | Low risk | No concerns | Major concerns | No concerns | No concerns | Low |
| BioTx:SPA | 0 | Some concerns | Low risk | No concerns | Major concerns | No concerns | No concerns | Low |
| BioTx:Sham | 0 | Some concerns | Low risk | No concerns | Major concerns | No concerns | No concerns | Low |
| BioTx:T-PNES | 0 | Some concerns | Low risk | No concerns | Major concerns | No concerns | No concerns | Low |
| BioTx:TPCI | 0 | Some concerns | Low risk | No concerns | Major concerns | No concerns | No concerns | Low |
| CSN:MI-CNS-NM | 0 | Some concerns | Low risk | No concerns | Major concerns | No concerns | No concerns | Low |
| CSN:MI-PNM | 0 | Some concerns | Low risk | No concerns | Major concerns | No concerns | No concerns | Low |
| CSN:MI-PNM + NB | 0 | Some concerns | Low risk | No concerns | Major concerns | No concerns | No concerns | Low |
| CSN:MI-PNM + SPA | 0 | Some concerns | Low risk | No concerns | Major concerns | No concerns | No concerns | Low |
| CSN:MOT | 0 | Some concerns | Low risk | No concerns | Major concerns | No concerns | No concerns | Low |
| CSN:MOT + NB | 0 | Some concerns | Low risk | No concerns | Major concerns | No concerns | No concerns | Low |
| CSN:PAN + NB | 0 | Some concerns | Low risk | No concerns | Major concerns | No concerns | No concerns | Low |
| CSN:PTEM | 0 | Some concerns | Low risk | No concerns | Major concerns | No concerns | No concerns | Low |
| CSN:SPA | 0 | Some concerns | Low risk | No concerns | No concerns | Major concerns | No concerns | Low |
| CSN:ST | 0 | Some concerns | Low risk | No concerns | No concerns | Major concerns | No concerns | Low |
| CSN:Sham | 0 | Some concerns | Low risk | No concerns | Major concerns | No concerns | No concerns | Low |
| CSN:T-PNES | 0 | Some concerns | Low risk | No concerns | No concerns | Major concerns | No concerns | Low |
| CSN:TPCI | 0 | No concerns | Low risk | No concerns | No concerns | Major concerns | No concerns | Low |
| MI-CNS-NM:MI-PNM + NB | 0 | Some concerns | Low risk | No concerns | Major concerns | No concerns | No concerns | Low |
| MI-CNS-NM:MI-PNM + SPA | 0 | Some concerns | Low risk | No concerns | Major concerns | No concerns | No concerns | Low |
| MI-CNS-NM:MOT | 0 | Some concerns | Low risk | No concerns | Major concerns | No concerns | No concerns | Low |
| MI-CNS-NM:MOT + NB | 0 | Some concerns | Low risk | No concerns | Major concerns | No concerns | No concerns | Low |
| MI-CNS-NM:NB | 0 | No concerns | Low risk | No concerns | Major concerns | No concerns | No concerns | Low |
| MI-CNS-NM:PAN + NB | 0 | Some concerns | Low risk | No concerns | Major concerns | No concerns | No concerns | Low |
| MI-CNS-NM:PTEM | 0 | No concerns | Low risk | No concerns | Major concerns | No concerns | No concerns | Low |
| MI-CNS-NM:SPA | 0 | Some concerns | Low risk | No concerns | Major concerns | No concerns | No concerns | Low |
| MI-CNS-NM:ST | 0 | Some concerns | Low risk | No concerns | Major concerns | No concerns | No concerns | Low |
| MI-CNS-NM:Sham | 0 | No concerns | Low risk | No concerns | Major concerns | No concerns | No concerns | Low |
| MI-CNS-NM:T-PNES | 0 | No concerns | Low risk | No concerns | Major concerns | No concerns | No concerns | Low |
| MI-CNS-NM:TPCI | 0 | No concerns | Low risk | No concerns | Major concerns | No concerns | No concerns | Low |
| MI-PNM:MI-PNM + NB | 0 | Some concerns | Low risk | No concerns | Major concerns | No concerns | No concerns | Low |
| MI-PNM:MOT | 0 | Some concerns | Low risk | No concerns | Major concerns | No concerns | No concerns | Low |
| MI-PNM:MOT + NB | 0 | Some concerns | Low risk | No concerns | Major concerns | No concerns | No concerns | Low |
| MI-PNM:NB | 0 | No concerns | Low risk | No concerns | Major concerns | No concerns | No concerns | Low |
| MI-PNM:PAN + NB | 0 | Some concerns | Low risk | No concerns | Major concerns | No concerns | No concerns | Low |
| MI-PNM:PTEM | 0 | No concerns | Low risk | No concerns | Major concerns | No concerns | No concerns | Low |
| MI-PNM:SPA | 0 | Some concerns | Low risk | No concerns | Major concerns | No concerns | No concerns | Low |
| MI-PNM:ST | 0 | Some concerns | Low risk | No concerns | Major concerns | No concerns | No concerns | Low |
| MI-PNM + NB:MI-PNM + SPA | 0 | Some concerns | Low risk | No concerns | Major concerns | No concerns | No concerns | Low |
| MI-PNM + NB:MOT | 0 | Some concerns | Low risk | No concerns | Major concerns | No concerns | No concerns | Low |
| MI-PNM + NB:MOT + NB | 0 | Some concerns | Low risk | No concerns | Major concerns | No concerns | No concerns | Low |
| MI-PNM + NB:PAN + NB | 0 | Some concerns | Low risk | No concerns | Major concerns | No concerns | No concerns | Low |
| MI-PNM + NB:PTEM | 0 | Some concerns | Low risk | No concerns | Major concerns | No concerns | No concerns | Low |
| MI-PNM + NB:SPA | 0 | Some concerns | Low risk | No concerns | Major concerns | No concerns | No concerns | Low |
| MI-PNM + NB:ST | 0 | Some concerns | Low risk | No concerns | Major concerns | No concerns | No concerns | Low |
| MI-PNM + NB:Sham | 0 | Some concerns | Low risk | No concerns | Major concerns | No concerns | No concerns | Low |
| MI-PNM + NB:T-PNES | 0 | Some concerns | Low risk | No concerns | Major concerns | No concerns | No concerns | Low |
| MI-PNM + NB:TPCI | 0 | No concerns | Low risk | No concerns | Major concerns | No concerns | No concerns | Low |
| MI-PNM + SPA:MOT | 0 | Some concerns | Low risk | No concerns | Major concerns | No concerns | No concerns | Low |
| MI-PNM + SPA:MOT + NB | 0 | Some concerns | Low risk | No concerns | Major concerns | No concerns | No concerns | Low |
| MI-PNM + SPA:NB | 0 | Some concerns | Low risk | No concerns | Major concerns | No concerns | No concerns | Low |
| MI-PNM + SPA:PAN + NB | 0 | Some concerns | Low risk | No concerns | Major concerns | No concerns | No concerns | Low |
| MI-PNM + SPA:PTEM | 0 | Some concerns | Low risk | No concerns | Major concerns | No concerns | No concerns | Low |
| MI-PNM + SPA:SPA | 0 | Some concerns | Low risk | No concerns | Major concerns | No concerns | No concerns | Low |
| MI-PNM + SPA:ST | 0 | Some concerns | Low risk | No concerns | Major concerns | No concerns | No concerns | Low |
| MI-PNM + SPA:Sham | 0 | Some concerns | Low risk | No concerns | Major concerns | No concerns | No concerns | Low |
| MI-PNM + SPA:T-PNES | 0 | Some concerns | Low risk | No concerns | Major concerns | No concerns | No concerns | Low |
| MI-PNM + SPA:TPCI | 0 | Some concerns | Low risk | No concerns | Major concerns | No concerns | No concerns | Low |
| MOT:MOT + NB | 0 | Some concerns | Low risk | No concerns | Major concerns | No concerns | No concerns | Low |
| MOT:NB | 0 | Some concerns | Low risk | No concerns | Major concerns | No concerns | No concerns | Low |
| MOT:PAN + NB | 0 | Some concerns | Low risk | No concerns | Major concerns | No concerns | No concerns | Low |
| MOT:PTEM | 0 | Some concerns | Low risk | No concerns | Major concerns | No concerns | No concerns | Low |
| MOT:SPA | 0 | Some concerns | Low risk | No concerns | Major concerns | No concerns | No concerns | Low |
| MOT:Sham | 0 | Some concerns | Low risk | No concerns | Major concerns | No concerns | No concerns | Low |
| MOT:T-PNES | 0 | Some concerns | Low risk | No concerns | Major concerns | No concerns | No concerns | Low |
| MOT:TPCI | 0 | Some concerns | Low risk | No concerns | Major concerns | No concerns | No concerns | Low |
| MOT + NB:NB | 0 | Some concerns | Low risk | No concerns | Major concerns | No concerns | No concerns | Low |
| MOT + NB:PAN + NB | 0 | Some concerns | Low risk | No concerns | Major concerns | No concerns | No concerns | Low |
| MOT + NB:PTEM | 0 | Some concerns | Low risk | No concerns | Major concerns | No concerns | No concerns | Low |
| MOT + NB:SPA | 0 | Some concerns | Low risk | No concerns | Major concerns | No concerns | No concerns | Low |
| MOT + NB:Sham | 0 | Some concerns | Low risk | No concerns | Major concerns | No concerns | No concerns | Low |
| MOT + NB:T-PNES | 0 | Some concerns | Low risk | No concerns | Major concerns | No concerns | No concerns | Low |
| MOT + NB:TPCI | 0 | Some concerns | Low risk | No concerns | Major concerns | No concerns | No concerns | Low |
| NB:PTEM | 0 | Some concerns | Low risk | No concerns | Major concerns | No concerns | No concerns | Low |
| NB:SPA | 0 | Some concerns | Low risk | No concerns | Major concerns | No concerns | No concerns | Low |
| PAN + NB:PTEM | 0 | Some concerns | Low risk | No concerns | Major concerns | No concerns | No concerns | Low |
| PAN + NB:SPA | 0 | Some concerns | Low risk | No concerns | Major concerns | No concerns | No concerns | Low |
| PAN + NB:ST | 0 | Some concerns | Low risk | No concerns | Major concerns | No concerns | No concerns | Low |
| PAN + NB:Sham | 0 | Some concerns | Low risk | No concerns | Major concerns | No concerns | No concerns | Low |
| PAN + NB:T-PNES | 0 | Some concerns | Low risk | No concerns | Major concerns | No concerns | No concerns | Low |
| PAN + NB:TPCI | 0 | No concerns | Low risk | No concerns | Major concerns | No concerns | No concerns | Low |
| PTEM:SPA | 0 | Some concerns | Low risk | No concerns | Major concerns | No concerns | No concerns | Low |
| PTEM:Sham | 0 | No concerns | Low risk | No concerns | Major concerns | No concerns | No concerns | Low |
| PTEM:T-PNES | 0 | Some concerns | Low risk | No concerns | Major concerns | No concerns | No concerns | Low |
| PTEM:TPCI | 0 | No concerns | Low risk | No concerns | Major concerns | No concerns | No concerns | Low |
| SPA:ST | 0 | Some concerns | Low risk | No concerns | Major concerns | No concerns | No concerns | Low |
| SPA:T-PNES | 0 | Some concerns | Low risk | No concerns | Major concerns | No concerns | No concerns | Low |
| SPA:TPCI | 0 | No concerns | Low risk | No concerns | Major concerns | No concerns | No concerns | Low |
| Sham:ST | 0 | Some concerns | Low risk | No concerns | Major concerns | No concerns | No concerns | Low |
| ST:T-PNES | 0 | Some concerns | Low risk | No concerns | Major concerns | No concerns | No concerns | Low |
| ST:TPCI | 0 | Some concerns | Low risk | No concerns | Major concerns | No concerns | No concerns | Low |
| Sham:T-PNES | 0 | Some concerns | Low risk | No concerns | Major concerns | No concerns | No concerns | Low |
| TPCI:T-PNES | 0 | Some concerns | Low risk | No concerns | Major concerns | No concerns | No concerns | Low |

**The overall confidence rating (High, Moderate, Low, Very Low) for each comparison was determined using the CINeMA framework, which algorithmically assesses concerns across six key domains (e.g., bias, imprecision, indirectness).** Full intervention names corresponding to the abbreviations used can be found in Supplementary Table S3.
