# Supplementary material for: Comparison of efficacy and safety of non-oral therapeutic interventions for zoster-associated pain: a systematic review and network meta-analysis
Source: Front Neurol. 2026 Jan 27;17:1711536. doi: 10.3389/fneur.2026.1711536 (PMC12886049; doi:10.3389/fneur.2026.1711536)
Supplement: Supplementary file 1 [file Data_Sheet_1.zip › Supplementary_Material_Complete/Table 5.docx]

**Table S5** List of Interventions with Codes and Abbreviations

| **Serial No.** | **Abbreviation** | **Full Intervention Name** |
| --- | --- | --- |
| 1 | ST | Standard treatment |
| 2 | NB | Nerve block |
| 3 | MI-PNM | Minimally invasive peripheral nerve modulation |
| 4 | Sham | Sham treatment |
| 5 | MI-CNS-NM | Minimally invasive central nervous system neuromodulation |
| 6 | TPCI | Topical and peripheral chemical interventions |
| 7 | PTEM | Physical therapy and energy medicine |
| 8 | SPA | Systemic pharmacological analgesia |
| 9 | T-PNES | Targeted peripheral nerve electrical stimulation |
| 10 | SEN | Superficial electrical neuromodulation |
| 11 | BioTx | Biological therapy |
| 12 | NI-CNS-NM | Non-invasive central nervous system neuromodulation |
| 13 | MOT | Medical oxidant therapy |
| 14 | CAM | Complementary and alternative medicine |
| 15 | CSN | Chemical selective neurolysis |
| 16 | MI-PNM + NB | Minimally invasive peripheral nerve modulation + Nerve block |
| 17 | PAN + NB | Physically-ablative neuromodulation + Nerve block |
| 18 | MI-PNM + SPA | Minimally invasive peripheral nerve modulation + Systemic pharmacological analgesia |
| 19 | MI-PNM + MOT | Minimally invasive peripheral nerve modulation + Medical oxidant therapy |
| 20 | MI-CNS-NM + TPCI | Minimally invasive central nervous system neuromodulation + Topical and peripheral chemical interventions |
| 21 | MOT + NB | Medical oxidant therapy + Nerve block |
| 22 | PTEM + NB | Physical therapy and energy medicine + Nerve block |

**The codes and abbreviations defined in this table are used in figures and tables to ensure conciseness.**
